# A Telemedicine App for Nonrigid Facial Rehabilitation Training Enhanced by Efficient Fully Convolutional Neural Network With Residual Network (EffiFCNN-ResNet) to Improve Accessibility for Patients With Nasopharyngeal Carcinoma Cancer: Randomized Controlled Trial

**DOI:** 10.2196/72560

**Published:** 2026-03-10

**Authors:** Tong Wu, Ting Han, Xiaoju Zhang, Yumei Dai, Xiaoyan Meng

**Affiliations:** 1 School of Design Shanghai Jiao Tong University Shanghai China; 2 Institute of Medical Robotics Shanghai Jiao Tong University Shanghai China; 3 Innovation Center of Yangtze River Delta Zhejiang University Jiaxing China; 4 Department of Nursing Fudan University Shanghai Cancer Center Shanghai China; 5 Department of Oncology Shanghai Medical College Fudan University Shanghai China

**Keywords:** nonrigid facial action tracking, fully convolutional neural network, telemedicine app, behavioral intervention

## Abstract

**Background:**

Resource limitations in public hospitals may hinder timely monitoring and management of rehabilitation in patients with nasopharyngeal carcinoma (NPC) after radiotherapy.

**Objective:**

This study developed and evaluated the telemedicine app “Open Care,” which integrates the Efficient Fully Convolutional Neural Network with Residual Network (EffiFCNN-ResNet) model and computer vision to monitor facial training exercises and provide real-time feedback, aiming to improve outcomes in patients with restricted mouth opening.

**Methods:**

Initially, the EffiFCNN-ResNet model underwent 5-fold cross-validation, expert validation, and robustness testing to assess its reliability and clinical applicability in complex real-world environments. Subsequently, to evaluate the telemedicine app, a parallel-group, 2-arm randomized controlled trial was conducted with 109 patients, who were randomly assigned to either the intervention group (n=55) or the control group (n=54). The intervention group performed mouth-opening exercises under the supervision and guidance of the telemedicine app, whereas the control group followed traditional video-based instructions. Primary outcome measures included maximum mouth opening, mouth-opening symmetry, exercise frequency, and rehabilitation-related health beliefs. Secondary outcomes included fatigue (Brief Fatigue Inventory), health-related quality of life (Assessment of Quality of Life—6 Dimensions), and system usability scores. Data were analyzed using 2-tailed (unpaired) independent-samples *t* tests and chi-square tests, and the Mann-Whitney U test was used to assess intra- and inter-group differences before and after the intervention.

**Results:**

The “Open Care” system leverages a lightweight fully convolutional neural network (FCNN) depth model integrated with network communication to enable real-time capture, recognition, and correction of nonrigid facial training movements. It also provides visual feedback and supports automated rehabilitation assessment. The model demonstrated strong generalization ability (macro-averaged F1-score, mean 0.96, SD 0.01) and clinical-grade stability (performance degradation: mean 5.2%, SD 0.6%, under lighting disturbances and challenging pathological cases; n=160 video segments). Compared with the control group, the intervention group showed significant improvements in maximum mouth opening (*P*=.04), exercise frequency (*P*=.001), perceived severity (*P*=.007), perceived benefits (*P*=.04), perceived barriers (*P*=.001), self-efficacy (*P*=.04), cues to action (*P*=.001), health behavior (*P*=.03), and fatigue (*P*=.04). Participants also reported favorable training experiences, with a mean system usability score of 74.3 out of 100.

**Conclusions:**

This telemedicine approach was more effective than traditional methods, improving patient engagement and rehabilitation outcomes while providing a more objective and precise monitoring tool. Future apps may benefit patients with NPC and other head and neck cancers.

**Trial Registration:**

Chinese Clinical Trial Registry ChiCTR2400090305; https://www.chictr.org.cn/showprojEN.html?proj=235073

## Introduction

### Background

Nasopharyngeal carcinoma (NPC) is a common malignant tumor of the head and neck, with a high prevalence in Eastern and Southeast Asian countries. Comprehensive treatment, primarily based on radiotherapy, is the preferred therapeutic approach for NPC [1]. Trismus is a common complication of radiotherapy [2,3], with an incidence ranging from 12.0% to 58.5% [4]. This complication is primarily caused by radiotherapy-induced fibrosis of the masticatory muscles and temporomandibular joint, muscle atrophy, and neural injury. It may also act synergistically with dysphagia, posing long-term challenges to patients’ nutritional intake and psychosocial well-being [5] and significantly affecting patients’ quality of life [6]. Current research on mouth-opening training and intervention for patients with NPC primarily focuses on the content, timing, methods, and tools of such exercises. Traditional clinical interventions commonly employ cork resistance training; however, patients often report significant pain perception [7]. To address this issue, a recent literature review [8] proposed a novel therapy combining hardware-assisted passive mouth-opening traction with transcutaneous neuromuscular electrical stimulation. However, contact-based training may exacerbate symptoms in patients with radiation-induced dermatitis or skin fibrosis [9]. Therefore, there is an urgent need to explore alternative approaches, particularly noncontact, visually interactive supportive interventions for patients with NPC after radiotherapy, to improve prognosis. Studies have demonstrated that early implementation of active mouth-opening exercises can effectively prevent the onset of trismus [10,11]. Moreover, Samarah et al [12] confirmed that targeted mouth-opening training interventions play a significant role in reducing the incidence of trismus, improving patient adherence, and enhancing quality of life in patients with NPC undergoing radiotherapy and chemotherapy [13]. Despite the proven efficacy of mouth-opening exercises, long-term treatment outcomes remain inconsistent [14], likely due to the high health care burden associated with training, movement correction, and supervision [15].

Digital health interventions can provide scalable health education and self-management support for patients with NPC through apps or web-based platforms [16,17], offering high coverage, low cost, and easy accessibility to mitigate constraints related to time and resources [18-21]. However, the effectiveness of some digital interventions varies. For instance, although physiotherapy programs and online apps have demonstrated efficacy in improving physical function [22], their long-term effectiveness declines due to a lack of precise, individualized support [23]. Additionally, a physical activity counseling program based on wearable devices reported no prognostic benefits, as it lacked accurate monitoring and feedback on training movements [24,25]. This limitation reduces the effectiveness of telemedicine apps in facilitating patient rehabilitation behaviors. A meta-analysis further highlighted that precise movement monitoring and targeted feedback are critical factors in enhancing the effectiveness of digital health interventions for patient health management. Therefore, future digital health interventions should prioritize advancements in personalized, intelligent, real-time monitoring and feedback technologies to enhance intervention efficacy [26,27].

In clinical practice, evaluation of jaw training in patients with limited mouth opening mainly relies on direct observation and manual measurements [28], which are limited in objectivity and real-time applicability. Recently, machine learning has shown promise in intelligent assessment and quantitative rehabilitation [29], integrating neural networks with medical imaging, treatment planning, patient simulation, quality assurance, and radiation dose delivery to provide objective monitoring in clinical care [30]. However, patients with NPC undergoing radiotherapy may experience skin fibrosis, inflammation, swelling, and mandibular deformities, which complicate facial motion analysis [31]. Additionally, factors such as head movement and lighting variations exacerbate nonrigid facial deformations, making accurate motion tracking difficult [32]. Existing methods that rely on contact-based markers are prone to causing facial trauma and patient discomfort and provide insufficient real-time tracking accuracy. Thus, developing a deep learning–based, noncontact, real-time monitoring system is crucial for enhancing NPC rehabilitation [33,34]. Recent advancements in artificial intelligence have enabled machines to automatically analyze and interpret complex data, supporting more personalized treatment strategies. For instance, He et al [35] explored dense facial tracking sequences using convolutional neural network (CNN) networks combined with nonrigid iterative closest point algorithms to address frame-to-frame relationships in 3D facial analysis. Durga et al [36] employed a hybrid deep learning algorithm, including MtCNN (multitask cascaded convolutional neural network) and DeepFace, to overcome nonrigid facial changes, such as variations in size, shape, and color. The authors proposed the Tiefes fully convolutional neural network (FCNN) model, achieving high accuracy in microexpression recognition for nonrigid facial movements, demonstrating the feasibility of CNN-based deep networks. Relatedly, Le et al [37] applied deep learning for human segmentation and tracking, whereas Singh et al [38] used a deep network to reduce the joint image–motion parameter search to a search limited to rigid motion parameters.

### Objectives

**Figure 1:**
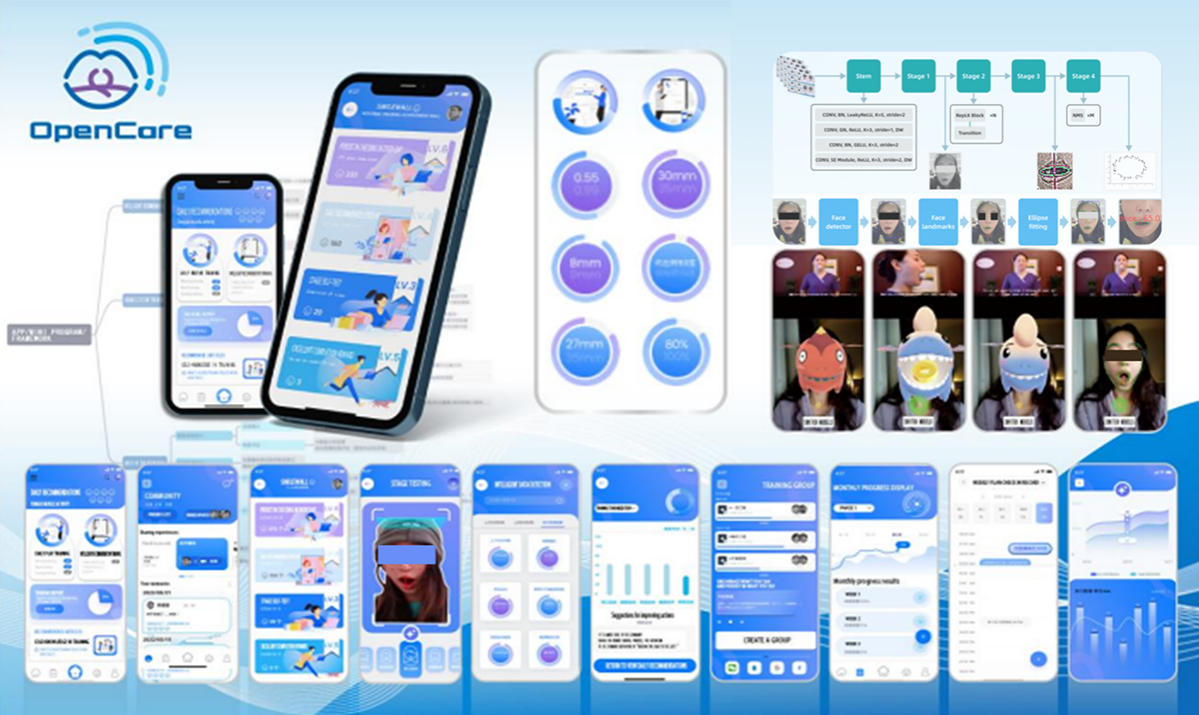
The overall design of Open Care.

To address the challenges in rehabilitation training for patients with NPC, we developed Open Care, a remote health care app designed specifically for NPC jaw rehabilitation (Figure 1). By utilizing an Efficient Fully Convolutional Neural Network with Residual Network (EffiFCNN-ResNet) model, Open Care enables noncontact, real-time facial motion tracking, measurement, and automated feedback. This system offers a novel solution for personalized and efficient NPC rehabilitation. A randomized controlled trial was conducted to evaluate its effectiveness in improving patient adherence and rehabilitation outcomes.

## Methods

### Tasks and Process: Involvement

All participants, regardless of group assignment, received initial standardized, equally timed exercise instruction from researchers to ensure consistent foundational guidance. Researchers provided detailed instructions on the key components of the training video and informed participants of the benefits of regular mouth-opening exercises.

The core component of standard care for the control group consisted of routine mouth-opening exercise guidance delivered through a 15-minute educational video, meticulously designed by radiation oncology professionals and validated by a multidisciplinary expert team. The video provided step-by-step demonstrations of active mouth-opening exercises, and participants were instructed to perform them twice daily, consistently within a pain-free range. An ancillary app was provided to the control group solely for video playback and for recording daily maximum mouth opening, symmetry, and training frequency.

Conversely, the experimental group used the OpenCare health app, which, while integrating the identical instructional videos, was augmented with an EffiFCNN-ResNet model. This advanced functionality enabled real-time facial movement recognition, monitoring, and automated feedback, providing users with intuitive visualizations of exercise completion, rehabilitation progress, and maximum mouth-opening values through graphical representations. The OpenCare app further incorporated structured, gamified guidance, featuring an interactive module that displayed both standard demonstrations and the participant’s live facial video for comparison. It provided immediate feedback, verbal cues for insufficient movement (eg, “Please try to increase the range of motion, hold longer”), and visual reinforcement, such as completion percentages and gamified animations. Beyond these advanced functionalities, OpenCare’s video materials and data-recording features were strictly identical to those used by the control group.

To clearly delineate the intervention’s impact, participants in both groups received weekly phone follow-ups of consistent content and duration to assess progress and address questions. Crucially, participants in both groups were strictly prohibited from engaging in any additional training beyond the prescribed regimen, thereby minimizing confounding factors.

### Procedure

#### Overview of the EffiFCNN-ResNet-Based System for Precise Training Monitoring and Assessment

**Figure 2:**
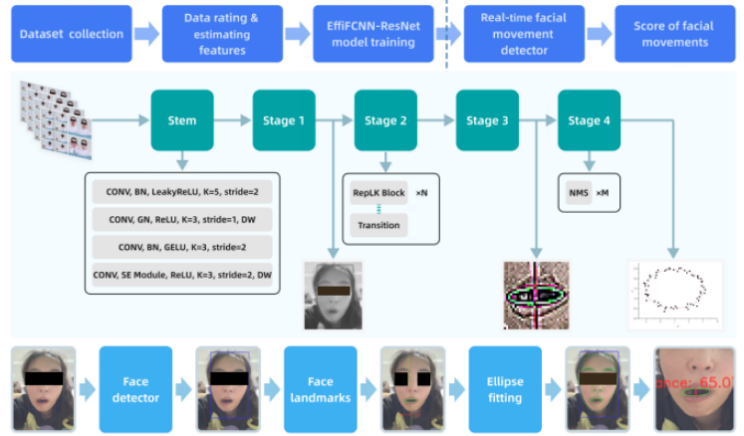
Architecture overview of the EffiFCNN-ResNet model for real-time facial landmark detection. BN: batch normalization; DW: depthwise; GELU: Gaussian error linear unit; EffiFCNN-ResNet: Efficient Fully Convolutional Neural Network with Residual Network; GN: group normalization; LK: large kernel; NMS: nonmaximum suppression; ReLU: rectified linear unit.

We developed the EffiFCNN-ResNet architecture to address the complex task of recognizing a large number of nonrigid facial landmarks [39-41]. Typically, increasing network depth or input image tensors is a common approach to overcoming challenges posed by strong environmental conditions and substantial facial structural changes during facial landmark monitoring [36]. However, this strategy often increases computational complexity and execution time. Our backbone network leverages the inherent advantages of FCNN in spatial data processing and incorporates a lightweight EffiResNet backbone designed for real-time applications requiring rapid and accurate facial detection and keypoint localization [42]. The underlying principle is rooted in a single-stage object detection framework, integrating a carefully designed detector with a lightweight backbone network to achieve fast and accurate facial recognition [40], as shown in Figure 2. The advantages and contributions of our model are summarized as follows:

- Custom training on the large-scale public movie dataset Acted Facial Expressions in the Wild (AFEW) using FCNN to detect facial landmarks in complex scene settings [43].
- Replacing the FCNN backbone with a rescaled EffiResNet backbone and utilizing large-kernel depthwise convolutions to expand the receptive field of the output feature map [44].
- Integrating the backbone network with channel separation and multinetwork landmark detection modules, followed by a linear spatial channel attention module combined with nonmaximum suppression to further optimize and filter features [45].

Specifically, our model consists of the components listed in the following subsections.

#### Backbone Network

A lightweight backbone network based on ResNet was used to extract salient features from input images. Its core component is the residual block, which addresses the vanishing gradient problem in deep networks by introducing skip connections, thereby improving training efficiency and overall model performance. The ResNet architecture demonstrates strong hierarchical feature extraction capability, enabling efficient capture of multiscale features from low to high levels. Designed according to the structural reparameterization principle, the backbone network consists of multiple residual blocks, each incorporating convolutional layers, batch normalization, and rectified linear unit activation functions. Skip connections allow each layer to directly receive input features from the preceding layer, enhancing training stability. The primary objective is to decouple the multibranch topology used during training from the simplified structure applied during inference. This backbone design achieves a balance between computational efficiency and feature expressiveness by separating the multibranch topology at training time from the streamlined architecture used at inference time.

#### Neck Network

The model integrates the classic spatial pyramid pooling (SPP) module and an improved path aggregation network (PAN), enhancing feature fusion capability and multiscale perception. The SPP layer performs multiscale pooling operations to integrate features across different scales, overcoming the limitation of fixed input image sizes and enabling the capture of multiscale contextual information to improve target region perception. After the SPP module, the feature map is transformed into a fixed-length output, providing richer contextual information for the subsequent PAN. The improved PAN uses a bottom-up routing fusion strategy, effectively reducing information loss during the transfer of deep-layer features to shallow layers. Adaptive feature pooling dynamically integrates feature maps, ensuring that information from different levels is fused across all feature scales. As a result, the features passed to the detection head contain comprehensive and semantically rich information, contributing to improved target detection accuracy.

#### Anchor Generation Module

This module generates dense anchors (proposed bounding boxes) at different scales and aspect ratios to detect nonrigid facial structures of varying sizes and shapes within the image. It is responsible for merging semantic features from deep layers with texture features from shallow layers and relies on an efficient decoupled head with an appropriate loss function.

#### Face Detection Head and Keypoint Localization Head

The detection head performs classification and regression of candidate boxes to optimize their positions and sizes. Meanwhile, the keypoint localization head accurately identifies facial landmarks, enhancing the algorithm’s applicability in tasks requiring detailed facial analysis while maintaining a balance between inference time and accuracy.

#### Dilated Convolution and Basic Block

These components are used to expand the receptive field during feature extraction while preserving spatial resolution.

#### Multiscale Detection

A key feature of the model is its multiscale detection strategy, which utilizes anchors generated on different feature maps to accommodate facial structures of varying sizes.

#### Concatenate (C) and Upsampling (U)

By combining low- and high-level features through feature fusion and upsampling, the model integrates features from different resolutions, ensuring robustness across a wide range of facial scales. This capability is critical for real-world deployment scenarios.

#### Joint Loss Function and FCOS Head

As the final detection head, the fully convolutional one-stage object detection (FCOS) head combines focal loss and generalized intersection over union (GIoU) loss to achieve efficient object classification and bounding box regression. The objective of the joint loss function is to simultaneously minimize classification error (facial region classification) and regression error (bounding box regression and keypoint localization), thereby optimizing network learning and improving the accuracy of both facial detection and keypoint localization. During deployment, the predicted probability labels for each sample are input to compute the focal loss, which is then averaged across all samples as the classification loss. GIoU is calculated to evaluate the intersection over union (IoU) between the predicted and ground truth bounding boxes. Subsequently, keypoint bounding box errors are removed, squared errors between predicted and true coordinates are calculated, and the results are averaged to obtain the keypoint localization loss. Self-monitoring of both losses constitutes a self-distillation technique, in which knowledge is transferred from the teacher model to the student model during training. The overall loss can be explained as follows:



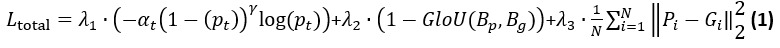



where –*α_t_*(1 – *p_t_*)*^γ^*log(*p_t_*) represents the action recognition loss, 1 – GloU(*B_p_*, *B_g_*) represents the facial region localization loss, and 
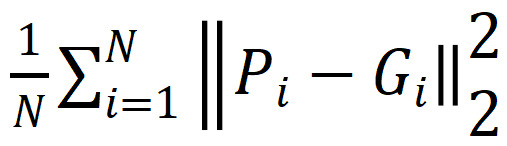
 represents the anatomical keypoint precision loss. \*λ*_1_, \*λ*_2_, and \*λ*_3_ are the corresponding adjustable weight coefficients for each term, used to balance the contribution of different tasks to the total loss, thereby adapting to varying optimization priorities during the rehabilitation training process. The term *λ*_1_ is designed based on an improved focal loss, primarily addressing the inherent challenge of severe class imbalance in rehabilitation training data, specifically between positive and negative samples. The modulating factor *γ* strengthens the weight assigned to hard-to-classify samples. Specifically, the (1 – *p_t_*)*^γ^* in the formula markedly amplifies the loss contribution of low-confidence samples (ie, when the predicted probability *p_t_* for the correct class approaches 0), while conversely reducing the contribution of easily classified examples (where *p_t_* approaches 1). Furthermore, *α_t_* serving as a class-balancing factor mitigates training bias arising from the disparity in the number of mouth-opening action frames versus nonaction frames. The term *λ*_2_ employs the GIoU loss, replacing the traditional IoU loss, with the aim of significantly enhancing the bounding box regression accuracy for dynamic facial regions, particularly the perioral area. The calculation of GIoU requires the simultaneous input of the coordinate parameters for both the predicted bounding box *B_p_* and the ground truth bounding box *B_g_*, as detailed in equation 2:

GLoU = LoU – [|*C*\(*B_p_*∪*B_g_*)|]/|*C*| **(2)**

Even in cases where the predicted and ground-truth bounding boxes are nonoverlapping, the GIoU loss can provide informative gradients by penalizing the difference between the smallest enclosing convex object (C) and the *B_p_*∪*B_g_*. For instance, when minor rotations of a patient’s head or variations in camera angle cause the predicted bounding box for the oral region to deviate from the ground-truth box without intersecting, GIoU still yields a directional gradient. This characteristic enhances the model’s robustness to changes in camera pose, distance, and subtle patient head movements. The term *λ*_3_ directly constrains the spatial deviation of facial anatomical keypoints through the mean squared error (MSE) formulation, ensuring high-precision landmark localization. Specifically, for the *i*th keypoint, the squared Euclidean distance between the predicted coordinate vector *P_i_* and the expert-annotated ground truth coordinate vector *G_i_* is computed, with the squared Euclidean distance calculated as 
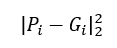
 for each keypoint. Subsequently, the error is averaged over N keypoints using the formula 
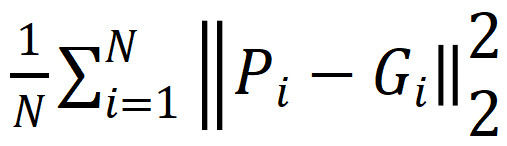
. This process ensures the model’s simultaneous and consistent optimization of all annotated points, including the upper lip, lower lip, and other crucial landmarks. This loss term directly prompts the model to precisely optimize the spatial localization consistency of all annotated points, including the upper lip, lower lip, and mouth corners.

#### Real-Time Capture

**Figure 3:**
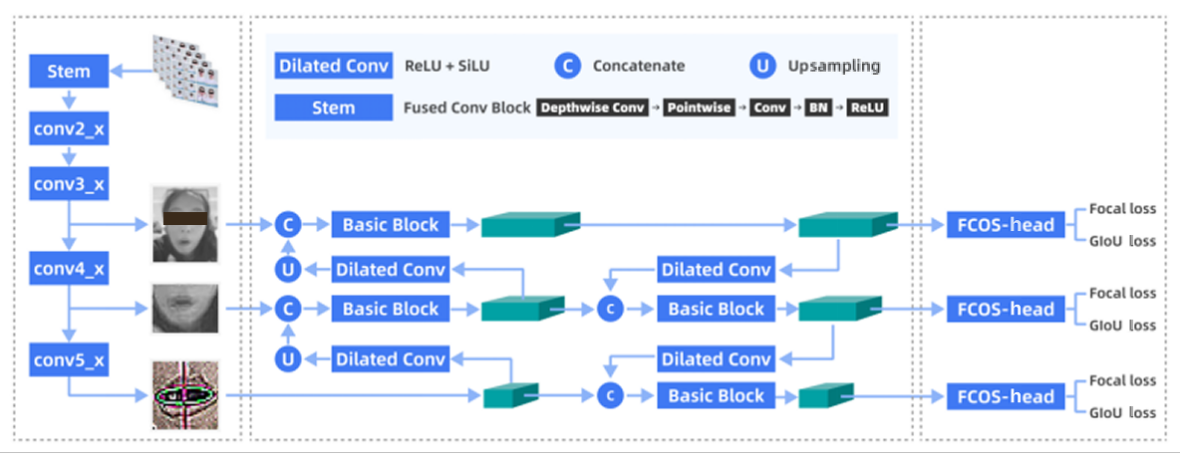
Real-time facial landmark detection with multiscale preprocessing and nonmaximum suppression optimization. BN: batch normalization; FCOS: fully convolutional one-stage object detection; GIoU: generalized intersection over union; ReLU: rectified linear unit; SiLU: sigmoid linear unit.

To enable rapid inference, the model employs multiscale preprocessing and multiscale detection, supplemented by optimization techniques such as nonmaximum suppression, making it suitable for real-time applications. Through the architecture described above, FCNN preserves spatial relationships across the entire network, enabling the generation of dense prediction maps. The lightweight ResNet extends convolutional layers throughout the network, facilitating direct correspondence between input pixel positions and their feature representations. Compared with traditional landmark detection methods, this approach captures a greater number of facial landmarks with higher accuracy while mitigating disturbances in texture and shape caused by nonrigid facial deformations, as illustrated in Figure 3. For rapid inference, the model adopts a lightweight ResNet-based object detection network enhanced with dilated convolutions. The core architecture uses multilevel dilated convolutions to expand receptive fields and capture contextual information, while integrating a stem module with lightweight pointwise convolutions and hybrid sigmoid linear unit/rectified linear unit activations to reduce computational complexity. Cross-level feature fusion is achieved through concatenation and upsampling operations to enhance detail retention. The FCOS head addresses class imbalance using focal loss and optimizes bounding box regression with GIoU loss, making it suitable for dense scenarios and small-object detection. Overall, the unified framework emphasizes 3 critical aspects: receptive field expansion through hierarchical dilation, multiscale feature interaction, and lightweight deployment capability, thereby balancing detection accuracy with computational efficiency.

### Training Action Classification and Evaluation

**Figure 4:**
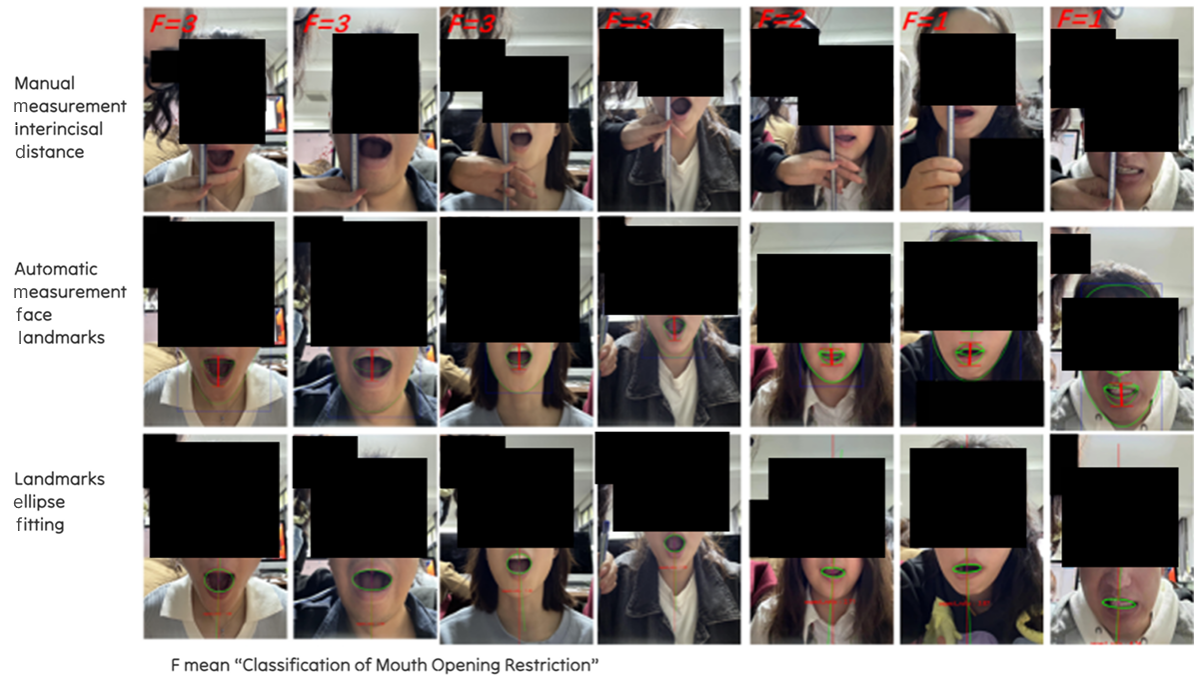
Action evaluation output results for a subset of the patient dataset.

Taking the mouth-opening action as an example, a more precise evaluation of movement is achieved by introducing a 4-level grading system that incorporates both the degree of mouth opening and its symmetry. This grading system simultaneously considers 2 key aspects: maximum mouth opening and mouth shape symmetry. Maximum mouth opening is defined as the vertical distance between the highest point of the upper semicircle and the lowest point of the lower semicircle, derived from the elliptical fitting of the mouth region. Symmetry is assessed based on the rotation angle of the fitted ellipse. To compute the elliptical equation of the oral region, we use a facial landmark detection and oral-region keypoint elliptical fitting algorithm based on the aforementioned model, utilizing more than 10,000 keypoints. This algorithm provides intelligent, automated evaluation of mouth-opening performance for each patient, as illustrated in Figure 4.

Through these steps, the keypoints obtained using the deep learning–based facial landmark localization algorithm can be further analyzed to assess the morphological characteristics of the oral region, thereby quantifying the degree of mouth opening and symmetry. The elliptical equation of the oral region can be expressed in the following general form:

(*x* – *x*_0_)^2^/*a*^2^ + (*y* – *y*_0_)^2^/*b*^2^ **(3)**

where (*x*_0_, *y*_0_) are the coordinates of the center of the ellipse, and *a* and *b* denote the lengths of the major and minor axes, respectively. Subsequently, the vertical distance between the highest point (*u*) of the upper semicircle and the lowest point (*d*) of the lower semicircle is calculated. The grading criteria were defined by oncologic medical experts. F4 indicates the normal range (35-50 mm), F3 represents mild restriction (25-35 mm), F2 represents moderate restriction (15-25 mm), and F1 represents severe restriction (<15 mm).

At the same time, the rotation angle of the ellipse is used to assess mouth symmetry. The rotation angle of the ellipse can be calculated using the following formula.

θ=arctan(bsin[Φ]/acos[Φ]) **(4)**

where Φ represents the inclination angle of the ellipse. The grading criteria for symmetry are defined as follows: F4, rotation angle <5° (good symmetry); F3, rotation angle 5°-10° (mild asymmetry); F2, rotation angle 10°-15° (moderate asymmetry); and F1, rotation angle >15° (severe asymmetry).

To correct for variations in the distance between the user and the camera, the 2 feature points at the inner corners of the eyes are connected to form a reference line. A standard value of 3.5 cm is defined, and a real-time scaling factor is derived by calculating the ratio between this reference distance and the actual measured value to perform distance correction, thereby facilitating subsequent data analysis. The corresponding code is shown below.

“# Input: Facial landmarks detected via EffiFCNN-ResNet

leftEye = (landmarks.part(39).x, landmarks.part(39).y) # Left eye corner (point 39)

rightEye = (landmarks.part(42).x, landmarks.part(42).y) # Right eye corner (point 42)

# Calculate Euclidean distance between eyes

eyeDistance = np.linalg.norm(np.array(leftEye) - np.array(rightEye)) # ||p_left - p_right||₂

# Compute scale factor using reference eye distance

scale = 3.5 / eyeDistance # d_reference = 3.5 (anthropometric mean)”

Intelligent recognition and evaluation of other training actions follow a similar approach. 

### Dataset

#### General Facial Representation Pretraining Phase

This phase used the AFEW dataset for training [46]. The AFEW dataset comprises 1200 video segments from real movies and clips, from which approximately 48,000 representative facial image frames were extracted. These frames include a wide range of individuals displaying diverse expressions under varying lighting conditions, poses, and background environments. The data modality consisted of RGB (red-green-blue) video frames and their corresponding emotional category labels. Optimization was performed using the AdamW optimizer with an initial learning rate of 1 × 10^–4^. A cosine annealing learning rate schedule, coupled with a warm-up phase, was applied. The model was trained for 80 epochs with a batch size of 32, incorporating gradient accumulation and various data augmentation techniques. Hyperparameters were determined through a combination of grid search and Bayesian optimization. This foundational training aimed to enhance the model’s generalization capability and prepare it for subsequent fine-tuning, particularly to achieve robust facial feature extraction in patient-specific scenarios.

#### Dataset Training Phase

The formal training dataset used in this study was derived from video data of real patients with NPC undergoing postradiotherapy rehabilitation training. The dataset comprised 800 video segments, from which approximately 12,000 keyframe images were extracted. Together with their corresponding annotations, this process generated 85,000 image-annotation pairs and 65,000 video-annotation pairs. To ensure robustness and generalizability of the developed model (EffiFCNN) in real-world clinical applications, the dataset was designed with a strong emphasis on diversity. Environmental diversity was achieved through simulated or real recordings of patients performing exercises at home using smartphones or tablets. These recordings encompassed a wide range of lighting conditions (eg, strong light, low light, side light), background complexities, camera angles (eg, frontal, top-down, bottom-up, side view), and shooting distances, thereby enhancing adaptability to complex daily environments. In terms of action content diversity, the dataset focused not only on the core mouth-opening rehabilitation exercise, comprehensively documenting maximal active mouth opening, sustained holding, and relaxation, but also included samples reflecting different degrees of mouth opening classified according to clinical F1-F4 grades and varying levels of symmetry quantified by the elliptical rotation angle *θ*. In addition, auxiliary rehabilitation exercises, such as neck movements, ocular movements, and cheek puffing (quantification methods are detailed in Multimedia Appendix 1), were incorporated to support a comprehensive rehabilitation assessment. Patient state diversity was a central characteristic of the dataset, with particular attention to challenges unique to postradiotherapy patients with NPC. The dataset covered all levels of mouth-opening restriction, ranging from severe restriction (F1, <15 mm) to normal function (F4, 35-50 mm). Special emphasis was placed on collecting samples from patients with moderate to severe restrictions (F1-F3), as this population represents the primary target group for intervention. The dataset also included samples exhibiting common postradiotherapy sequelae, such as skin hyperpigmentation, fibrosis, edema, facial asymmetry, and subtle jaw deformities, all of which pose significant challenges for nonrigid facial deformation analysis. To address class imbalance, the synthetic minority over-sampling technique was applied to augment minority class samples within the feature space, thereby improving recognition performance for rare categories. Within ethical and privacy constraints, efforts were made to include patients across diverse age groups, genders, and body types to enhance model generalizability. During data preprocessing, all patient-identifiable information was deidentified (Figure 4), and strict adherence to ethical guidelines was maintained, with approval obtained from the Institutional Review Board (approval number 2404294-Exp8). Finally, the dataset was partitioned into training, validation, and test sets (70%:15%:15%) using stratified sampling based on key clinical features. This approach ensured sample independence and distributional consistency, providing a robust foundation for model training and evaluation.

Despite concerted efforts to ensure diversity in its design, the dataset remains susceptible to potential biases. These are explained in the following sections.

#### Sampling Bias

This bias may arise from the participant recruitment process, which primarily involved voluntary patients capable of using smart devices. Such an approach may lead to the underrepresentation of patients who are critically ill, severely mobility-impaired, digitally illiterate, or socioeconomically disadvantaged. In addition, the concentration of data sources within specific hospitals or regions may introduce bias related to variations in medical practice patterns.

#### Annotation Bias

Annotation bias may result from the inherent subjectivity in clinical experts’ assessments of mouth-opening grades (F1-F4) and symmetry (rotation angle θ). Moreover, precise keypoint annotation becomes substantially more challenging in patients with severe postradiotherapy facial sequelae, such as pronounced edema or deformity.

#### Temporal Bias

This type of bias reflects variations associated with different postradiotherapy recovery phases (eg, acute, subacute, chronic) or heterogeneous baseline conditions, such as initial mouth-opening capacity.

### Validation of Precision

To ensure rigorous and objective evaluation of the proposed EffiFCNN-ResNet model, we implemented a comprehensive validation strategy combining stratified 5-fold cross-validation with an independent test set. The dataset, comprising 800 video segments from patients with NPC, was first stratified by patient ID and mouth-opening restriction grade (F1-F4) into 560 (70%) segments for training, 120 (15%) segments for validation, and 120 (15%) segments for testing. The cross-validation procedure employed Scikit-learn’s StratifiedGroupKFold method to ensure that all 448 (80% of the training set) segments were used for model training in each fold, while 112 (20%) segments served as validation data. This approach prevented patient-level data leakage across folds and maintained balanced-grade distributions throughout the validation process.

The model demonstrated consistent performance across all validation metrics. In classification tasks, it achieved a mean accuracy of 89.2% (SD 2.4%; 95% CI 86.8%-91.6%) for mouth-opening restriction grades (as detailed in equation 5), with a macro-averaged *F*_1_-score of 0.875 (SD 0.031) and an area under the receiver operating characteristic curve of 0.934 (SD 0.018). For regression tasks, the mean absolute error for mouth-opening distance was 1.23 mm (SD 0.15 mm; 95% CI 1.08-1.38 mm), as detailed in equation 6, while the rotation angle *θ* showed a mean absolute error of 2.15° (SD 0.32°; 95% CI 1.83°-2.47°). Keypoint detection achieved a normalized mean error of 0.043 (SD 0.006), with a failure rate of 3.8% (SD 1.2%; 95% CI 2.6%-5.0%). On the independent test set comprising 120 video segments from 45 patients, the model achieved an accuracy of 98.3% (118/120 correctly classified segments; 95% CI 97.6%-98.8%), with particularly strong performance on severely restricted F1-grade samples (96.4% recall; 27/28 cases correctly identified). In robustness testing involving patients with significant postradiotherapy sequelae, the model maintained 94.4% accuracy (151/160 challenging samples correctly classified). Expert annotations established a reliable gold standard with excellent interrater reliability (intraclass correlation coefficient 0.942; 95% CI 0.925-0.956) among 20 clinical oncologists. The Bland-Altman analysis demonstrated excellent agreement with expert measurements (mean difference −0.14 mm; 95% limits of agreement −1.86 to 1.58 mm), and the intraclass correlation coefficient reached 0.968 (95% CI 0.953-0.979), confirming the model’s clinical reliability and readiness for deployment in diverse rehabilitation settings.

Accuracy = (TP + TN)/(TP + TN + FP + FN) **(5)**

where TP is true positive, which represents the number of actual positive samples correctly predicted as positive; TN is true negative, which represents the number of actual negative samples correctly predicted as negative; FP is false positive, which represents the number of negative samples incorrectly predicted as positive; and FN is false negative, which represents the number of positive samples incorrectly predicted as negative.



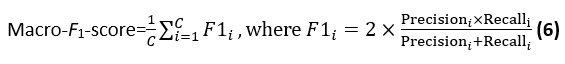



where *C* denotes the number of classes (F1-F4), emphasizing the model’s balanced evaluation of performance across all individual categories.

To rigorously evaluate the clinical applicability of the EffiFCNN-ResNet system, comprehensive robustness testing was conducted under simulated challenging conditions encompassing both environmental and patient-specific variations. Environmental perturbations were introduced through structured image transformations, including +30% or –30% linear and gamma-adjusted illumination shifts in the hue-saturation-value color space and affine transformations simulating +15° or –15° viewpoint deviations in pitch, yaw, and roll angles. A dedicated subset containing 160 video samples (20% of the test set; N=800), consisting exclusively of postradiotherapy patients with NPC exhibiting pronounced sequelae such as skin fibrosis, edema, and facial asymmetry, was used to assess performance under clinically relevant morphologic distortions. The model demonstrated exceptional stability, with an overall performance degradation of 5.2% (SD 0.6%) under combined disturbances. Specifically, there was a 0.007 increase in normalized mean error for landmark detection (SD 0.002), a 0.4 mm increase in mouth-opening distance mean absolute error (SD 0.1), and 94.5% accuracy retention across F1-F3 restriction grades (SD 2.1%). These findings confirm the model’s ability to maintain high accuracy and reliability under real-world variability, underscoring its suitability for deployment in clinical and home-based rehabilitation settings.

**Figure 5:**
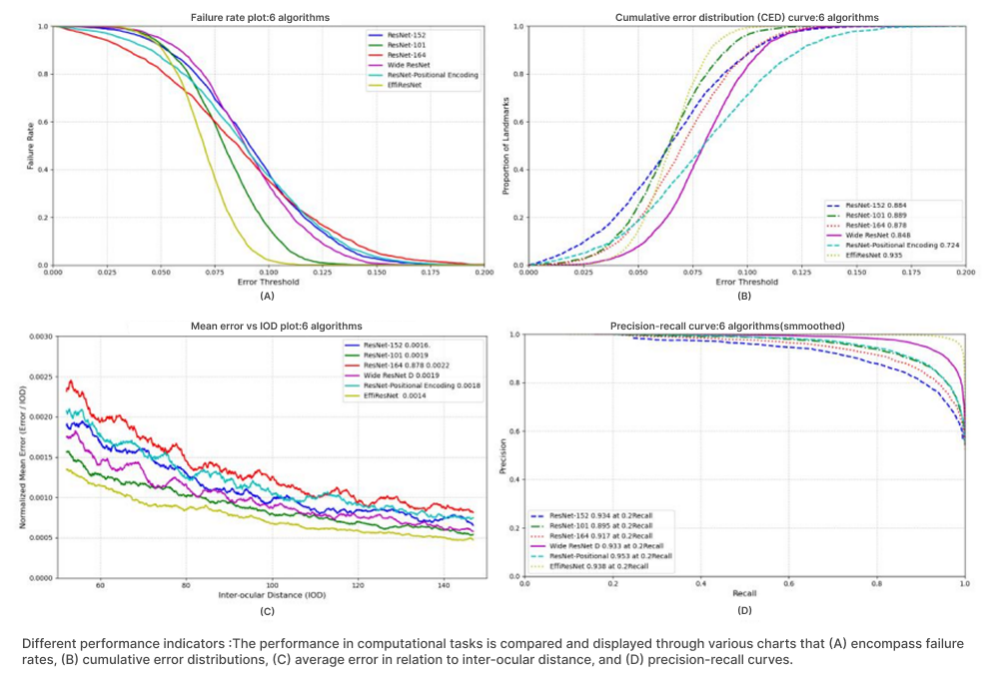
Model performance test results. IOD: interocular distance.

In the final validation stage, the superiority of the proposed method was objectively evaluated through systematic comparative experiments against multiple ResNet architecture variants, including ResNet-101, ResNet-152, ResNet-164, Wide ResNet, and ResNet-Positional Encoding. These comparisons were conducted on both the public AFEW dataset and a self-collected mouth-opening training dataset from patients with NPC, with standardized environments and data preprocessing to ensure fairness. Four evaluation metrics were employed: failure rate, cumulative error distribution, average error relative to interocular distance, and precision-recall curves. All models were assessed using identical hardware configurations and datasets. The performance of the proposed model is presented in Figure 5. The comparison of EffiResNet with other mainstream ResNet variants demonstrates its superior ability to balance accuracy, robustness, and computational efficiency. In terms of failure rate, EffiResNet shows a markedly faster decline as the error threshold increases compared with other models, indicating stronger robustness and adaptability when handling tasks with larger error tolerances. This characteristic enables stable performance in complex and dynamic computational environments. Regarding cumulative error distribution, the EffiResNet curve is smooth and approaches the cumulative error distribution performance of ResNet-152, suggesting stable behavior across varying error magnitudes. Compared with other ResNet variants, EffiResNet more effectively balances the influence of different error scales during accumulation, thereby ensuring overall performance stability. From a spatial awareness perspective, the relationship between average error and interocular distance demonstrates that the EffiResNet curve remains relatively stable, highlighting its advantage in spatial perception. This enables more accurate capture and analysis of spatial information in tasks involving spatial positioning. In classification tasks, EffiResNet also exhibits strong performance. Precision-recall curve comparisons indicate that, relative to ResNet-Positional Encoding, EffiResNet maintains high recall while achieving comparatively high precision. This reflects its ability to accurately identify target categories while minimizing misclassification.

### Personalized Real-Time Feedback

**Figure 6:**
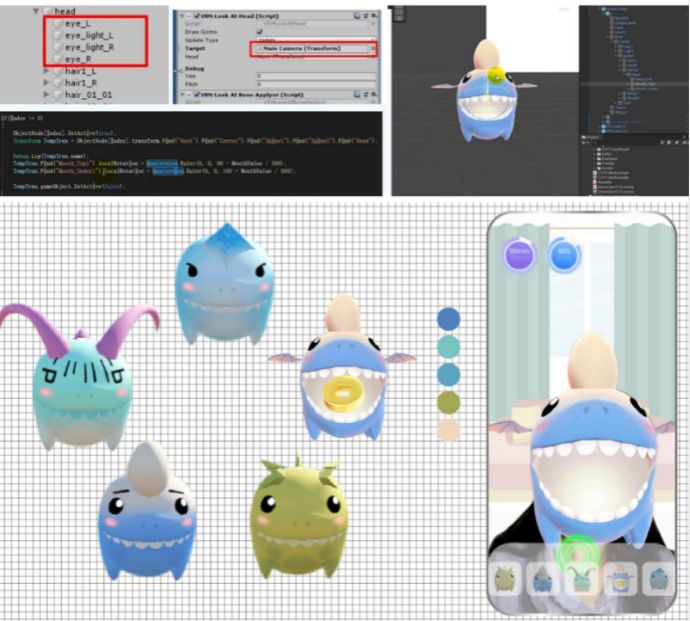
Training virtual headgear and its development program demonstration.

In designing the virtual avatar for patients with nasopharyngeal cancer, we considered potential deficiencies in the sensory integration system while avoiding the uncanny valley effect [47]. A series of brightly colored, soft, rounded dinosaur-themed virtual headgear avatars with simple structures was developed [48]. These avatars are linked to mouth-opening training movements through gamification based on skeletal keypoint capture, providing real-time visual and auditory feedback (eg, swallowing coins) when the required movement is achieved (Figure 6). As training scores accumulate, users can unlock additional virtual avatars as rewards. This design aims to stimulate patients’ intrinsic motivation during training and create an immediate, supportive, and personalized rehabilitation environment. The remote mouth-opening data tracking function was developed using the Unity3D engine (Unity Technologies) and Python (Python Foundation), with communication implemented through SocketTools.cs. Upon user authorization, the mobile device camera is used for facial landmark detection, enabling the system to collect mouth-opening data over a specified period, including maximum mouth opening, opening frequency, and symmetry. These data are transmitted to the backend via transmission control protocol/IP sockets for processing, with serial communication facilitating data exchange between devices and enabling real-time monitoring of patient movements. Data transmission is secured using encryption protocols to ensure privacy protection. The collected data support long-term daily monitoring of mouth-opening rehabilitation progress and provide actionable feedback to both patients and health care professionals regarding training patterns and behavioral habits.

Furthermore, personalized feedback is provided according to each patient’s functional level. Before each training session, an initial mouth-opening test is conducted to categorize patients into specific levels. Based on this classification, the performance thresholds required to complete the prescribed training tasks are individualized and progressively adjusted according to the severity of the condition, enabling a more flexible and targeted rehabilitation approach. This strategy ensures that the training protocol is tailored to the unique needs of each patient, thereby facilitating more effective recovery and sustained progress.

### Individuals Involved

The study participants comprised patients with nasopharyngeal cancer who had completed radiotherapy and were preparing for discharge from the oncology department. Participants were recruited through posters and questionnaire screening.

The inclusion criteria were as follows: (1) a pathology or histologic diagnosis of nasopharyngeal cancer with a prescribed course of radiotherapy; (2) age 18-65 years; (3) awareness of their medical condition; (4) provision of informed consent and willingness to participate in the study; (5) access to a smartphone and related apps; and (6) Functional Status Score (Karnofsky performance status) of 80-100 [49] and Eastern Cooperative Oncology Group performance status of 0-5 [50].

The exclusion criteria were as follows: (1) a history of prior radiotherapy; (2) inability to participate in physical activity due to underlying cardiac, neurological, muscular, or joint disorders; and (3) previous engagement in relevant mouth-opening exercises (eg, as part of routine daily care, follow-up video guidance, or other structured rehabilitation programs).

The withdrawal criteria were defined as follows: (1) voluntary decision to discontinue participation in the trial; (2) development of a severe illness that precluded continuation of the study; (3) occurrence of an adverse event related to mouth-opening exercises or routine physical activity; and (4) requirement for additional surgical interventions, such as mandibular resection.

### Following Testing

After the 4-week intervention period, all participants underwent a comprehensive assessment, including measurement of maximal mouth opening as well as other predefined outcome measures and relevant clinical indicators. All data were collected using the same measurement procedures as those employed during the baseline assessment, ensuring methodological consistency. This approach enabled reliable quantitative analysis for both within- and between-group comparisons.

### Size of Sample

In our previous comparative study, the sample size was calculated based on the change in perceived benefit scores measured 1 week after the intervention. Pilot data demonstrated that the intervention group showed a mean increase of 0.8 points (from mean 10.5, SD 2.6 to mean 11.3, SD 2.4), corresponding to a Cohen *d* effect size of 0.33. Using G*Power (Heinrich Heine University Düsseldorf) for sample size estimation, we determined that a total sample of 108 participants (54 per group) would be required to achieve 80% statistical power [51] at a 2-tailed significance level of 5% [52].

### Blind Method, Randomization, and Allocation Concealing

This study rigorously employed a stratified block randomization approach to allocate eligible participants to either the experimental or control group, ensuring balanced distribution of key baseline characteristics while preserving allocation unpredictability. An independent statistician generated the randomization sequence using R (version 4.4.x; R Foundation). Stratification variables included age (<45, 45-60, and >60 years), gender (male or female), and cancer stage (stage II and below, stage III, and stage IV). These factors were selected based on their clinical relevance and prognostic significance, while avoiding excessive stratification that could result in sparse subgroup sizes [53]. To further enhance allocation concealment, varying block sizes (4 or 6) were applied [54]. Each group assignment was placed in an opaque, sealed, and consecutively numbered envelope prepared and safeguarded by the independent statistician. Envelopes were opened sequentially by unblinded study personnel only after completion of participant screening and informed consent procedures. This process ensured that recruiting staff, intervention providers, and outcome assessors remained unaware of group allocation prior to assignment, thereby effectively minimizing selection bias [55]. Additionally, a triple-blind design was implemented, whereby participants, outcome assessors, and statisticians were blinded to group allocation. This strategy reduced the risk of performance and ascertainment biases, substantially enhancing the internal validity, objectivity, and reliability of the study findings.

### Documents of Safety

No adverse events were reported in association with this app during the study period. Participants were instructed that if they experienced significant respiratory distress, palpitations, or any other form of physical discomfort during the intervention, they must immediately discontinue the training session and return to a resting position. If symptoms were severe or persisted, participants were advised to seek prompt medical evaluation. All serious adverse events were reported to the institutional ethics committee in accordance with regulatory requirements, while any unexpected or nonserious adverse events were documented and reported to the research team for further evaluation and follow-up.

### Outcomes Measures

#### Questionnaire

A validated questionnaire, administered either online or in person, was completed by each participant at baseline and again 4 weeks after the intervention. No financial compensation or other incentives were provided for participation in the study. The collection of baseline demographic data and related variables is described in detail later.

#### Primary Outcomes

##### Maximum Mouth Aperture Measurement

To evaluate the effectiveness of the intervention app on mouth-opening function, participants’ maximum mouth aperture was measured and compared before and after the intervention. This comparison enabled assessment of functional improvement attributable to the training program. During measurement, participants were instructed to open their mouths as widely and steadily as possible to reach their individual maximal range. Once the maximum opening position was achieved, a 3-second stabilization period was maintained. Five data points were collected during the middle 1-second interval to minimize transient fluctuations. The average of these 5 values was calculated and recorded as the final maximum mouth-opening measurement.

##### Evaluation of Mouth Aperture Symmetry

To assess the impact of the intervention on oral motor function, the symmetry of participants’ mouth-opening movements was documented and compared before and after the intervention. Symmetry was evaluated by analyzing bilateral movement patterns during mouth opening, allowing identification of deviations or asymmetries in mandibular motion. Changes in symmetry measurements between baseline and postintervention assessments were used to determine the effectiveness of the app in improving coordinated oral function.

##### Number of Exercises Per Week

Adherence to the prescribed training behavior was quantitatively evaluated by recording and comparing the participants’ weekly exercise frequency before and after the intervention. The total number of completed mouth-opening training sessions per week was documented to assess behavioral implementation and engagement. Changes in weekly training frequency served as an indicator of intervention adherence and behavioral improvement.

##### Health Belief Model Questionnaire Inquiry Form

The Health Belief Model Questionnaire is widely used to assess health-related behaviors [56]. The version developed by social psychologists in the United States comprises 7 core constructs of the Health Belief Model: perceived susceptibility (3 items), perceived severity (7 items), perceived benefits (3 items), perceived barriers (7 items), self-efficacy (5 items), cues to action (3 items), and behavior (3 items) [57]. For all subscales except the behavior domain, responses were rated on a 5-point Likert scale ranging from “completely disagree” (0) to “completely agree” (4). Participants were asked to evaluate statements reflecting their health beliefs across the 6 domains. The behavior subscale, designed with strong psychometric properties, assesses the frequency of relevant health behaviors (eg, mouth-opening exercises) using a scale ranging from “never” (0) to “always” (4). Higher scores indicate greater engagement in the targeted health behavior.

#### Secondary Cognitive Outcomes

##### Borg Rating of Perceived Exertion Scale

The Borg Rating of Perceived Exertion Scale [58] is used to quantify self-reported physical exertion during exercise. The scale score ranges from 6 (no exertion at all) to 20 (maximal exertion). Participants rate their perceived intensity of effort during mouth-opening training, providing a subjective measure of exercise load.

##### Assessment of Quality of Life—6 Dimensions

Health-related quality of life was assessed using the Assessment of Quality of Life—6 Dimensions (AQoL-6D) [59] instrument. Scores range from −0.04 to 1.00, with higher scores indicating better quality of life. This measure captures multiple domains of well-being to evaluate the broader impact of the intervention.

##### System Usability Scale

Perceived usability of the app was evaluated using the System Usability Scale (SUS) [60]. After completing the designated training tasks, participants completed the SUS questionnaire to obtain an overall usability score. Higher SUS scores indicate better perceived system usability and user satisfaction.

### Data Analysis

Data were analyzed using SPSS (IBM Corp.). Descriptive statistics were presented as means with SDs for continuous variables, and as frequencies (n) with percentages (%) for categorical variables. Baseline characteristics of participants who completed the study were compared with those who did not using 2-tailed (unpaired) independent-samples *t* tests or chi-square tests, as appropriate. Given the distribution characteristics of the variables, the Mann-Whitney *U* test was used to evaluate differences between groups, and within-group comparisons before and after the intervention were also conducted accordingly. Comparisons were performed between the intervention and control groups to determine the effectiveness of the intervention. For all statistical analyses, inter- and intragroup differences were expressed as means with 95% CIs. The significance level (α) was set at .05, and a *P* value ≤.05 was considered statistically significant.

### Ethical Considerations

This study was conducted in accordance with the ethical principles outlined in the Declaration of Helsinki and received approval from the institutional Human Research Ethics Committee (protocol number: 2404294-Exp8; reference number: H20240007I). All participants provided written informed consent before enrollment after receiving a detailed explanation of the study’s objectives and procedures. They were explicitly informed that participation was entirely voluntary and that they could withdraw from the study at any time without any negative consequences. To ensure privacy and confidentiality, all collected data were fully anonymized before analysis, and no personally identifiable information was collected or retained. Additionally, no financial compensation or other incentives were provided, thereby maintaining a transparent and voluntary recruitment process.

## Results

### Characteristics at Baseline

**Figure 7:**
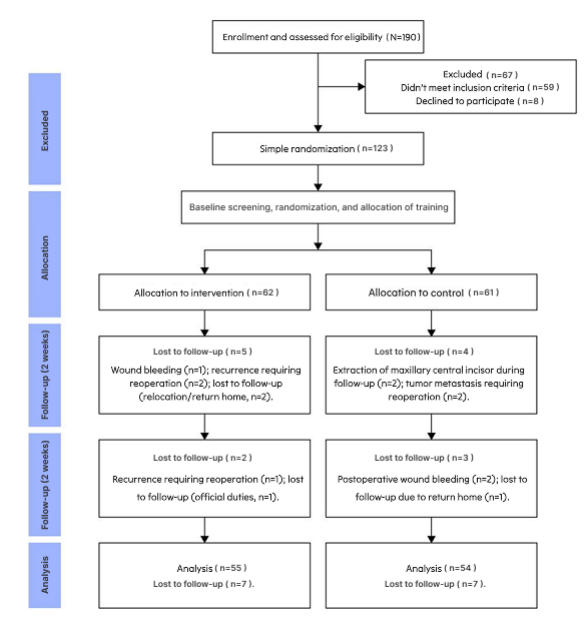
CONSORT (Consolidated Standards of Reporting Trials) flow diagram.

A total of 190 patients were screened for eligibility, of whom 67 did not meet the inclusion criteria and were excluded. Written informed consent was obtained from all participants. During the study, 7 participants in the app-based intervention group were excluded for the following reasons: postoperative wound bleeding (n=1); postoperative recurrence requiring reoperation (n=3); and loss to follow-up due to relocation, official duties, or return to hometown (n=3). In the routine care control group, 7 participants were excluded for the following reasons: extraction of the maxillary central incisor during follow-up (n=2), tumor metastasis requiring reoperation (n=2), postoperative wound bleeding (n=2), and loss to follow-up due to return to hometown (n=1). Ultimately, 109 valid cases were included. In accordance with the CONSORT (Consolidated Standards of Reporting Trials) flow diagram (Figure 7; see also Multimedia Appendix 2) [61], participants in both groups were analyzed according to their original allocation (intervention group: n=55; control group: n=54).

Before beginning the intervention, disease-related data (cancer type, treatment method, course of disease, number of mouth-opening recovery exercises per week, and household residence), as well as name, gender, age, and education level, were collected from all participants. Baseline measurements were conducted 1 week before the commencement of the intervention, focusing on maximum mouth opening and other scores (Table 1). Males comprised the majority of the sample (75/109, 68.8%), with a mean age of 47.31 (SD 8.47) years, which was consistent with the prevalence of NPC. Table 1 presents outcomes related to NPC-related indicators, maximal mouth opening, mouth-opening symmetry, weekly training frequency, gender, age, education level, and the Shapiro-Wilk test. There were no significant differences between groups (see Table 1), indicating comparability and suitability for subsequent experiments and analyses. At baseline, based on the evaluation of participants’ cancer-related function and the potential impact on quality of life after radiotherapy, mouth-opening training was supported.

**Table 1:** Baseline characteristics.

Variables			Intervention group (n=55)		Control group (n=54)		*P* value	
Age			46.55 (7.98)		48.06 (8.94)		.43	
**Gender, n (%)**								.53
	Male	41 (74.5)		34 (63.0)				
	Female	14 (25.5)		20 (37.0)				
**Education level**								.93
	Lower secondary and below	11 (20.0)		9 (16.7)				
	High school	18 (32.7)		18 (33.3)				
	Tertiary and above	26 (47.3)		27 (50.0)				
**Family residence**								.77
	City	32 (58.2)		36 (66.7)				
	Countryside	23 (41.8)		18 (33.3)				
**Marital status**								.69
	Unmarried	7 (12.7)		9 (16.7)				
	Married	48 (87.3)		45 (83.3)				
**Monthly income (Yuan^a^)**								.81
	<3000	7 (12.7)		4 (7.4)				
	3001-5000	25 (45.5)		29 (53.7)				
	>5001	23 (41.8)		21 (38.9)				
**Cancer stage, n (%)**								.74
	Ⅱ and below	7 (12.7)		11 (20.4)				
	Ⅲ	18 (32.7)		16 (29.6)				
	Ⅳ	30 (54.5)		27 (50.0)				
Number of chemotherapy sessions, mean (SD)			3.13 (1.98)		3.33 (1.60)		.75	
Number of radiotherapy sessions, mean (SD)			22.43 (1.60)		22.55 (1.95)		.11	
**Duration of disease (months), n (%)**								.84
	≤5	11 (20.0)		16 (29.6)				
	6-11	18 (32.7)		14 (25.9)				
	12-17	16 (29.1)		18 (33.3)				
	≥18	10 (18.2)		6 (11.1)				
**Number of exercises per week, n (%)**								.81
	≤4	34 (61.8)		38 (70.4)				
	5-8	18 (32.7)		14 (25.9)				
	≥9	3 (5.5)		2 (3.7)				
Maximum value of mouth opening (mm), mean (SD)			36.98 (1.61)		37.05 (1.63)		.88	
Evaluation of mouth opening symmetry, mean (SD)			0.836 (0.086)		0.841 (0.079)		.67	
Karnofsky performance status, mean (SD)			82.16 (10.80)		81.85 (16.45)		.44	
Eastern Cooperative Oncology Group, mean (SD)			2.55 (1.85)		2.65 (1.65)		.48	

^a^1 yuan=US $0.14.

### Outcomes

#### Primary Outcomes

Table 2 shows changes in the Health Belief Model subscales and clinical indicators of improved jaw clenching from baseline to the 4-week trial period after the intervention. For perceived susceptibility, although changes in both the intervention and control groups did not reach statistical significance (*P*=.07), the intervention group score increased from 6.0 at baseline to 7.5, indicating a certain degree of positive change. This contrasted with the small change observed in the control group (from 5.8 to 6.1). For perceived severity, the mean value in the intervention group increased from 23.3 to 27.8, with a difference of 4.5 (95% CI 3.6-5.4; *P*=.007), indicating a significant difference, whereas the control group showed little change, increasing only from 24.6 to 25.4. For perceived benefits, the mean score in the intervention group increased from 11.2 to 11.9 (difference 0.7; 95% CI 0.2-1.2), which was statistically significant (*P*=.04). At the same time, the reduction in perceived impairment was particularly significant (from 15.5 to 8.5; difference −7.0; 95% CI −8.1 to −5.9; *P*=.001). In addition, self-efficacy increased from 5.4 to 6.4 (difference 1.0; 95% CI 0.6-1.4; *P*=.04), and action cues increased from 7.5 to 11.2 (difference 3.7; 95% CI 3.2-4.2; *P*=.001), indicating that the intervention successfully improved individuals’ confidence and motivation to adopt healthy behaviors. The increase in maximum mouth opening (from 36.98 to 39.05; difference 2.07; 95% CI 1.49-2.61; *P*=.04) and improvements in physical indicators related to oral health demonstrated the potential benefits of the intervention in promoting specific health behaviors. The intervention group performed significantly more exercises per week (mean 10.0, SD 3.5) than the control group (mean 6.7, SD 2.3; *P*=.001). However, small changes in mouth-opening symmetry (−0.014; 95% CI −0.024 to 0.005; *P*=.15) suggested that the intervention was not equally effective across all outcomes.

**Table 2:** Primary and secondary intervention results.

Results		Intervention (n=55)				Control (n=54)				*P* value
		Baseline, mean (SD)	Follow-up, mean (SD)	Difference (95% CI)	Baseline, mean (SD)		Follow-up, mean (SD)	Difference (95% CI)		
**Primary outcome**										
	Perceived susceptibility	6.0 (3.4)	7.5 (1.8)	1.5 (0.4 to 2.6)	5.8 (3.4)		6.1 (2.9)	0.3 (–1.2 to 1.8)	.07	
	Perceived severity	23.3 (3.8)	27.8 (2.6)	4.5 (3.6 to 5.4)	24.6 (3.6)		25.4 (2.7)	0.8 (0.3 to 1.9)	.007	
	Perceived benefits	11.2 (1.2)	11.9 (1.4)	0.7 (0.2 to 1.2)	10.9 (1.3)		10.8 (1.6)	–0.1 (–0.7 to 0.5)	.04	
	Perceived barriers	15.5 (5.5)	8.5 (5.2)	–7.0 (–8.1 to –5.9)	15.4 (6.4)		15.5 (5.9)	0.1 (–3.5 to 3.7)	.001	
	Self-efficacy	5.4 (1.1)	6.4 (1.2)	1.0 (0.6 to 1.4)	5.9 (1.1)		5.5 (1.2)	–0.4 (–0.9 to –0.1)	.04	
	Cues to action	7.5 (1.8)	11.2 (2.9)	3.7 (3.2 to 4.2)	7.7 (1.6)		7.2 (1.4)	–0.5 (–1.1 to 0.1)	.001	
	Action (behavior)	6.6 (2.7)	8.1 (2.8)	1.5 (0.9 to 2.1)	6.4 (2.6)		5.9 (2.8)	–0.5 (–1.3 to 0.3)	.03	
	Number of exercises per week	4.2 (2.6)	10.0 (3.5)	5.8 (4.8 to 6.8)	3.4 (2.6)		6.7 (2.3)	3.3 (1.9 to 4.6)	.001	
	Maximum value of mouth opening (mm)	36.98 (1.61)	39.05 (1.38)	2.07 (1.49 to 2.61)	37.05 (1.63)		38.02 (1.54)	0.97 (0.49 to 1.41)	.04	
	Evaluation of mouth opening symmetry	0.836 (0.086)	0.822 (0.048)	–0.014 (–0.024 to 0.005)	0.841 (0.079)		0.838 (0.076)	–0.003 (–0.023 to 0.017)	.15	
**Secondary cognitive outcomes**										
	Borg Rating of Perceived Exertion Scale score	11.85 (1.11)	12.98 (0.76)	1.13 (0.81 to 1.45)	11.65 (0.66)		13.43 (1.13)	1.78 (1.33 to 2.23)	.04	
	Assessment of Quality of Life—6 Dimensions	0.46 (0.19)	0.48 (0.18)	0.02 (–0.04 to 0.08)	0.55 (0.17)		0.52 (0.21)	–0.03 (–0.09 to 0.03)	.18	

#### Secondary Cognitive Outcomes

The control group experienced a significant increase in perceived exertion, with a mean difference in Borg Rating of Perceived Exertion Scale score of 1.78 (95% CI 1.33-2.23; *P*=.04), indicating a notable rise in fatigue levels compared with the intervention group. By contrast, changes in quality of life, as measured by AQoL-6D, were not significant in either group (*P*=.18). System availability was evaluated using the SUS, and the experimental group achieved a mean usability score of 74.3 out of 100. Scores exceeding 70 were classified as good, indicating high system availability.

## Discussion

### Principal Findings

This study evaluated Open Care, a telemedicine app based on a deep learning model integrated with computer vision, for real-time monitoring and feedback of facial training exercises. The app aims to enhance rehabilitation outcomes in patients with nasopharyngeal cancer following radiation therapy. By leveraging the lightweight EffiFCNN-ResNet deep learning architecture and integrated network communication technologies, Open Care effectively captures and corrects nonrigid facial training movements in real time. It also provides intuitive visual feedback and automated rehabilitation assessments through computer vision. The results indicate that, following use of the app, patients with nasopharyngeal cancer showed significant improvements in clinical metrics, including maximum mouth opening, training frequency for mouth opening, perceived severity, perceived benefits, perceived barriers, self-efficacy, action cues, behavior, and perceived fatigue, compared with the control group. These findings suggest that the Open Care app holds substantial potential for replicating successful rehabilitation interventions in patients with nasopharyngeal cancer. Consistent with existing literature, this study reinforces the critical role of structured, feedback-driven rehabilitation in alleviating trismus and improving quality of life for patients with NPC [62,63]. Our findings align with recent remote intervention studies, which highlight that integrating motivational elements and structured feedback significantly enhances patient exercise adherence and functional recovery outcomes [64-66]. This research further validates the positive impact of guided, regular training on symptom amelioration.

However, the “Open Care” methodology and technological pathway employed in this study diverge significantly from existing work. Most current related research predominantly relies on contact sensors or hardware devices (eg, wearable sensors or specialized rehabilitation equipment) coupled with periodic remote health education for intervention [67,68]. Outcome assessment in these approaches is largely based on manual measurements (eg, caliper measurement of mouth opening) and patient self-report questionnaires. While these traditional methods possess certain value, they generally exhibit inherent limitations: self-reported measurements are susceptible to subjective bias [69], real-time continuous monitoring of movement quality is challenging [70], intervention in daily patient training is often delayed [71], and hardware dependence elevates the barrier to entry and cost [72]. By contrast, “Open Care” facilitates entirely contactless, computer vision–based real-time motion analysis and immediate corrective feedback. It leverages high-efficiency deep learning models to automatically extract facial movement features and quantify similarity to standard movements for scoring, thereby mitigating human measurement error, minimizing intervention invasiveness, and significantly enhancing patient engagement and enjoyment [73]. This is likely a key reason why the experimental group in this study demonstrated more pronounced improvements in objective metrics [74]. Thus, unlike previous studies solely focused on remote follow-up education or single-assessment dimensions, the assessment methodology of “Open Care” demonstrates distinct innovation and advantages. It enables multimodal data fusion assessment, integrating objective training data (eg, movement accuracy and adherence) with subjective patient-reported outcomes, providing a more scientific and comprehensive quantification of rehabilitation efficacy [75]. Furthermore, Open Care represents an advancement in technological integration and interaction paradigms, innovatively incorporating real-time computer vision feedback into the clinical practice of remote rehabilitation for NPC. By offering an immersive and gamified training experience, the system effectively enhances patients’ long-term adherence [73], a feat that is challenging to achieve with traditional remote education or simple video guidance [76].

Further in-depth analysis of the intervention data indicates that, in terms of objective clinical indicators, our clinical trial reported significant differences in maximum mouth opening and frequency of mouth-opening exercises, a promising finding that demonstrates better clinical outcomes than those reported in previous remote support studies [77-79]. After the intervention, maximum mouth opening increased from 36.98 to 39.05, corresponding to improvements in physical indicators related to oral health. In the intervention group, the mean increase in maximum mouth opening was 2.07 mm, with a median increase ranging from 1.5 to 2.6 mm. In addition, within 4 weeks after discharge, the number of patients with clenched jaws decreased, and the magnitude of this change was comparable to findings from previous studies involving patients with head and neck cancer [80,81], indicating the potential benefits of the intervention in improving specific health behaviors. Furthermore, treatment adherence is a critical determinant of the success of any preventive exercise program [15,82]. We observed that the intervention group performed significantly more exercises per week (mean 10.0, SD 3.5) than the control group (mean 4.2, SD 2.6), demonstrating the significant impact of our developed app on improving patient adherence to home rehabilitation exercises. However, despite the significant improvement in maximum mouth opening, 11 of 109 (10.1%) participants in the experimental group still exhibited clenched jaws during follow-up, suggesting that a longer intervention period may be required [66]. In addition, our study did not find that the app was associated with improvement in mouth-opening symmetry, which contrasts with previous studies. This discrepancy may be partly attributable to differences in study duration and in the type and level of functional support provided [83]. A comprehensive strengthening of the facial nerve structure and musculature is required for symmetry to improve, and short-term interventions are ineffective [84].

In terms of improvements in rehabilitation training behaviors, the remote health care intervention group in this study, compared with the standard care control group, demonstrated significant enhancements in patients’ perceived benefits and self-efficacy. These findings are consistent with previous studies [85-87], including one by Schlieter et al [88], which examined the impact of remote health care–based educational programs on improving oral health training behaviors in pregnant women. The results of that randomized controlled trial showed significant improvements in oral health beliefs and clinical training indicators, similar to our study, suggesting that remote health care interventions can effectively improve individuals’ health cognition and behavior change [89]. This supports the rational use of such models to improve health behaviors across a wide range of environments and populations [90,91]. The remote health care tool developed in this study provides patients with real-time supervision of training movements, corrective prompts, and periodic visual training score reports. This semisupervised, freely accessible intervention appears to be an effective approach for enhancing perceived benefits and self-efficacy [92]. Compared with traditional educational methods, it is more cost-effective and offers greater penetration [93]. The significant improvements in Perceived Severity and Perceived Benefits further validate the effectiveness of the intervention. Our app, using the EffiFCNN-ResNet deep learning model and computer vision, tracks participants’ mouth-opening movements to facilitate appropriate exercise and training plans while providing personalized feedback for patients at different levels. This may also effectively promote the growth of self-efficacy among patients [94], thereby contributing to clinical evidence for personalized health care design indicators in remote health care, as reported in a meta-analysis [95].

This study yielded more substantial changes in action cues and perceived barriers than previous research. In addition, there was a significant increase in the frequency of weekly mouth-opening training behavior. This confirms the promising potential of health apps that use computer vision for personalized training correction and feedback [96]. These apps overcome the spatial limitations of traditional methods and provide more effective interaction and facilitation for monitoring, feedback, and motivation in rehabilitation training [73]. Integrating the activation of behavioral cues into digital intervention tools aims to guide patients with nasopharyngeal cancer to implement training as a health behavior by prompting and facilitating modifications to their decision-making framework [97]. This finding is consistent with previous studies [98-100]. The implementation of multiple strategies can effectively bridge the gap between patients and professional rehabilitation knowledge, reduce training costs, diminish perceived barriers to mouth training as a health behavior, narrow the gap between training cognition and practice, and encourage patients to demonstrate commitment to health behaviors through mouth training. Consistent with the findings of Tore et al [101], telerehabilitation yields a considerably higher standard of physical therapy than self-management. This may be attributable to the duration and quality of training tasks [101,102]. However, there was no statistically significant increase in perceived susceptibility. This may be attributable to the fact that participants in the sample shared a common disease group, which tends to result in more consistent perceptions of specific health hazards [103]. Nonetheless, perceived susceptibility continued to increase after the intervention, which may indicate the intervention’s potential.

Furthermore, we propose a reliable and objective intelligent assessment method for mouth-opening rehabilitation in clinical practice. A deep learning model, EffiFCNN-ResNet, was developed to address the needs of nonrigid, markerless, real-time facial capture, tracking correction, and automated assessment for patients with nasopharyngeal cancer. By combining the spatial processing strengths of FCNNs with a lightweight EffiResNet backbone [104], our model achieves fast and accurate facial detection, addressing the trade-offs between computational complexity and execution time. Performance analysis shows that EffiResNet demonstrates strong robustness, quickly reducing failure rates as the error threshold increases, in contrast to other ResNet variants [105,106], which are less adaptable in error-prone environments. The model shows stable performance across varying error magnitudes and excels in dynamic spatial changes [107]. In classification tasks, EffiResNet outperforms other ResNet variants in recall without sacrificing precision. Overall, EffiFCNN-ResNet excels in robustness, stability, and spatial awareness, offering an efficient solution for real-time facial landmark detection in complex environments. Its design enhancements, including the rescaled ResNet backbone and large-kernel depthwise convolutions, significantly contribute to its performance, positioning it as a promising tool for advanced facial recognition tasks. However, it is important to note that, due to certain objective factors such as limited data availability, the model may occasionally exhibit errors in specific situations. These errors primarily result from insufficient diversity in the training data or a lack of specific facial action samples, which can affect the model’s accuracy when encountering rare or abnormal actions. Nonetheless, these occasional errors are not substantial enough to notably degrade overall model performance. With continued data collection and model training, these errors are expected to diminish, thereby further enhancing the model’s precision.

It is noteworthy that participants reported positive feedback regarding their training experience with the Open Care app, along with a significant reduction in fatigue levels. This positive user experience not only attests to the app’s utility and user-friendliness but also highlights its important role in promoting sustained patient engagement in rehabilitation training. Because of the potential presence of uncontrolled external factors during the intervention period, such as other treatments received by participants and changes in lifestyle habits, these elements may have interfered with the study outcomes, particularly for indicators closely related to daily quality of life, such as AQoL-6D [108]. Nevertheless, based on responses from the user experience questionnaire, the researchers confirmed that the app successfully achieved its objectives of precise training movement monitoring and targeted feedback. The majority of respondents regarded the electronic exercise intervention as beneficial. They believed that incorporating interactive design and real-time feedback into exercise programs enhances enjoyment while also encouraging individuals to take the initiative to engage in this healthy behavior. Regarding comfort, most participants reported that the electronic exercise intervention was more personalized and better aligned with their individual rehabilitation needs. Thus, patients with NPC who have undergone radiotherapy may benefit from telemedicine apps that use deep learning and computer vision technologies [109], and this potential should not be overlooked.

Furthermore, Open Care offers substantial advantages in terms of comprehensive benefits and clinical utility. Compared with conventional rehabilitation models that primarily rely on outpatient visits and manual supervision, this app, through its automated real-time monitoring and feedback mechanisms [110], significantly reduces dependence on specialized rehabilitation therapists. This, in turn, minimizes long-term human resource investment and associated costs, aligning with current health care policy orientations toward efficient, accessible, and intelligent services [111], and serving as an important decision-making factor for health care providers when considering new therapeutic interventions [112]. At the same time, because training and assessment can be completed remotely, the frequency of patient hospital visits is substantially reduced. This not only saves transportation, escort, and time-related costs for patients but also alleviates patient load on health care institutions [113]. From a broader health system perspective, this standardized and scalable intervention approach is particularly well-suited for resource-limited regions. It enables expanded service coverage and equity while maintaining rehabilitation quality by leveraging digital means to enhance high-quality service delivery and decentralize health care resources, thereby optimizing overall resource allocation efficiency [114]. More importantly, through continuous and objective data collection, Open Care offers the potential for early intervention and dynamic adjustment of rehabilitation plans. This proactively mitigates the risk of secondary health complications and additional medical expenditures that may arise from improper or delayed rehabilitation [115]. From the perspective of health care payers, such preventive, data-driven health management tools help control overall health care expenditures, consistent with the ongoing policy shift from treatment-centric to health-centric approaches [116]. Consequently, Open Care not only provides immediate cost-saving benefits but also, in the long term, strengthens the resilience and value proposition of health care services, addressing the shared objectives of multiple stakeholders, including patients, health care institutions, payers, and governmental bodies.

### Limitations and Future

However, our investigation is not without limitations. First, the sample size was limited and comprised individuals from several nearby tumor departments. This may have resulted in population clustering, which is not ideal for deriving generalizable conclusions. Furthermore, during the pre-experiment phase, some patients with limited digital health proficiency were unable to independently troubleshoot the equipment used in the experimental procedure. In addition, as an intervention app based on an augmented reality environment, the system may be unsuitable for patients who are sensitive to sensory stimulation at the design level. Moreover, although the trial demonstrated short-term user engagement, the long-term sustainability of engagement and activity remains uncertain. The advancement of tools for capturing, monitoring, and evaluating nonrigid facial models must contend with a wide range of human facial expressions and movements, including complex mouth movements and subtle eye and eyebrow motions. The recognition accuracy of the model we developed still has room for improvement. Precisely capturing and tracking these subtle variations is particularly challenging under low-resolution or real-time conditions. Resource limitations may also affect system response time and user experience [117]. The experiment lasted only 4 weeks; moving forward, it will be essential to extend the intervention duration and further refine the app design to optimize outcomes. In conclusion, future research should investigate the applicability of Open Care for patients with NPC living in diverse and complex environments and at different stages of rehabilitation. Furthermore, in light of ongoing technological advances and integration of user feedback, continuous refinement and expansion of app functionalities will be critical to ensuring sustained effectiveness and broad applicability.

### Conclusion

This study successfully developed “Open Care,” a novel telemedicine app using the EffiFCNN-ResNet deep learning model and computer vision for real-time facial motion tracking and assessment in NPC rehabilitation. Our randomized controlled trial demonstrated significant efficacy in improving objective clinical metrics, such as maximum mouth opening and training frequency, as well as a comprehensive range of patient-reported outcomes, including perceived severity, perceived benefits, perceived barriers, self-efficacy, action cues, enhanced training behavior, and reduced perceived fatigue. These findings highlight the substantial potential of “Open Care” to enhance patient adherence, functional recovery, and quality of life in NPC rehabilitation, offering an accessible, cost-effective, and scalable digital solution for clinical practice, particularly in resource-limited settings. Future research will focus on refining model accuracy, extending intervention duration, and exploring applicability to broader head and neck cancer populations, further strengthening the role of artificial intelligence–driven telemedicine in oncological supportive care.

## Appendix

Multimedia Appendix 1 Specific fitting functions.

Multimedia Appendix 2 CONSORT eHEALTH checklist (v 1.6.1).

## Abbreviations

AFEW: Acted Facial Expressions in the Wild
AQoL-6D: Assessment of Quality of Life—6 Dimensions
CNN: convolutional neural network
CONSORT: Consolidated Standards of Reporting Trials
EffiFCNN-ResNet: Efficient Fully Convolutional Neural Network with Residual Network
FCNN: fully convolutional neural network
FCOS: fully convolutional one-stage object detection
FN: false negative
FP: false positive
GIoU: generalized intersection over union
IoU: intersection over union
MtCNN: multitask cascaded convolutional neural network
NPC: nasopharyngeal carcinoma
PAN: path aggregation network
RGB: red-green-blue
SPP: spatial pyramid pooling
SUS: System Usability Scale
TN: true negative
TP: true positive

## Data Availability

The datasets used or analyzed during this study are available from the corresponding author (TH) on reasonable request.
