# Supplementary material for: A Telemedicine App for Nonrigid Facial Rehabilitation Training Enhanced by Efficient Fully Convolutional Neural Network With Residual Network (EffiFCNN-ResNet) to Improve Accessibility for Patients With Nasopharyngeal Carcinoma Cancer: Randomized Controlled Trial
Source: JMIR Mhealth Uhealth. 2026 Mar 10;14:e72560. doi: 10.2196/72560 (PMC13014076; doi:10.2196/72560)
Supplement: Multimedia Appendix 1 [file mhealth_v14i1e72560_app1.docx]

**Multimedia Appendices**

# TABLE

**Table S1. Fitting Calculation of Intelligent Recognition and Evaluation for Other Training Actions**

| **Action Content** | **Action Recognition Modeling** | **Automated Action Evaluation Modeling** | | | |
| --- | --- | --- | --- | --- | --- |
| **Neck Activity**  Keep the head upright and slowly turn it to one side, ensuring the shoulders remain stationary, until a mild stretch is felt in the neck. Hold for 3 seconds, then slowly turn the head to the other side. This exercise helps improve neck flexibility, reduce tension, stiffness, and pain. | By analyzing the changes in the head keypoints at different time points, the rotation angle θ of the head is calculated.  θ=arccos$\left( \frac{P_{1}\cdot P_{2}}{\left\vert P_{1} \right\vert\left\vert P_{2} \right\vert} \right)$ (1)  Here, P_1_ and P_2_ refer to the vectors of the midpoint of the inner eye corner line and the midpoint of the fitted ellipse of the mouth, respectively. θ denotes the head rotation angle. | **Dimension** | **Standard** | **W** | **S** |
|  |  | Rotation Angle | Mild: < 15°  Moderate: 15° - 30°  Severe: > 30° | 50% | 1-10  points |
|  |  | Rotation Speed | Fast: > 30°/s  Moderate: 15° - 30°/s  Smooth: < 15°/s | 50% | 1-10  points |
| **Eye Activity**  Keep the head still and move the eyeballs in a clockwise direction to draw a circle, followed by a counterclockwise direction. This exercise helps alleviate dryness and stiffness symptoms after eye radiotherapy. | By utilizing the outer corner eye landmark, the direction of eyeball movement is determined by calculating the changes in the horizontal and vertical angles of the eye $\theta_{\mathrm{horizontal}}$.  $\theta_{\mathrm{horizontal}}=\arctan\left( \frac{y_{\mathrm{outer}}-y_{\mathrm{inner}}}{x_{\mathrm{outer}}-x_{\mathrm{inner}}} \right)$ (2)  Here, ($x_{\mathrm{inner}}$,$y_{\mathrm{inner}}$) and ($x_{\mathrm{outer}}$,$y_{\mathrm{outer}}$) refer to the coordinates of the inner and outer corners of the eye, respectively. By calculating the change in the angle at each frame, the direction of the eye movement is determined.  $c_{\mathrm{score}}=\frac{\min\left( t_{\mathrm{clockwise}},t_{\mathrm{counterclockwise}} \right)}{\min\left( t_{\mathrm{clockwise}},t_{\mathrm{counterclockwise}} \right)}$ (3)  $c_{\mathrm{score}}$ refers to the completion of the action, $t_{\mathrm{clockwise}}$ is the duration of clockwise motion, and $t_{\mathrm{counterclockwise}}$ is the duration of counterclockwise motion." | Completen-ess | Complete: The duration and angle change of clockwise and counterclockwise movements are nearly the same, with the difference being less than the 10% threshold.  Partially Complete: The duration of either clockwise or counterclockwise movement is shorter, with the difference exceeding the threshold (10%-30%).  Incomplete: The periodic movement is not completed successfully. | 50% | 1-10  points |
|  |  | Symmetry | Fully Symmetric: The angle change, speed, and duration of clockwise and counterclockwise movements are almost identical.  Partially Symmetric: The difference between clockwise and counterclockwise movements is between 10%-20%.  Asymmetric: There is a significant difference in the angle between clockwise and counterclockwise movements, with an imbalanced movement trajectory. | 50% | 1-10  points |
| **Cheek Puffing Exercise**  Fill the cheeks with air evenly, causing the face to expand as much as possible to the sides. Hold for 5 seconds to reduce facial muscle stiffness or functional limitations caused by treatment. | By calculating the horizontal distance change between the left and right corners of the mouth, the degree of expansion on both sides of the face is assessed. In the normal state, the horizontal distance between the corners of the mouth is the baseline value. During puffing, the horizontal distance should increase, and the cheeks will expand outward.  Initially, the horizontal distance between the mouth corners is d _initial_, and after puffing, it is d_expanded._The degree of expansion is then calculated as follows:  f=$\frac{d_{\mathrm{expanded}}-d_{\mathrm{initial}}}{d_{\mathrm{initial}}}\times1$00% (4) | Range of Motion | Fully Expanded: The degree of expansion is greater than the 30% threshold, indicating sufficient expansion.  Partially Expanded: The degree of expansion is between 10%-30%.  Slight Expansion: The degree of expansion is below 10%.  No Expansion: No noticeable facial expansion. | 60% | 1-10  points |
|  |  | Sustainabil-ity | Action Duration:  5 seconds or more  3-4 seconds  1-2 seconds  Less than 1 second: Score 0 | 40% | 1-10  points |
| **Test Action: Mouth Opening Exercise**  The calculation and evaluation method for this action have been detailed in the previous section. | During the measurement, the user is required to progressively apply force to open the mouth as wide as possible, until reaching their maximum mouth opening range, holding for 3 seconds before returning to a neutral position. During the middle second, 5 data points are collected and averaged to calculate the maximum mouth opening value. | | | | |
| Each training action is repeated 5-10 times, with a 5-second pause between actions. After completing each training session, a test action is performed.  Grading Criteria: F4: 7.5-10 points, F3: 5-7.4 points, F2: 2.5-4.9 points, F1: 0-2.4 points (established collaboratively by clinical experts). | | | | | |

“Intelligent recognition and evaluation of other training actions follow a similar approach. The specific fitting functions are shown in Table S1.(Multimedia Appendices).”
