# Supplementary material for: A Telemedicine App for Nonrigid Facial Rehabilitation Training Enhanced by Efficient Fully Convolutional Neural Network With Residual Network (EffiFCNN-ResNet) to Improve Accessibility for Patients With Nasopharyngeal Carcinoma Cancer: Randomized Controlled Trial
Source: JMIR Mhealth Uhealth. 2026 Mar 10;14:e72560. doi: 10.2196/72560 (PMC13014076; doi:10.2196/72560)
Supplement: Multimedia Appendix 2 [file mhealth_v14i1e72560_app2.pdf]

CONSORT-EHEALTH (V 1.6.1) - Submission/Publication Form

The CONSORT-EHEALTH checklist is intended for authors of randomized trials evaluating web-based and Internet-based applications/interventions, including mobile interventions, electronic games (incl multiplayer games), social media, certain telehealth applications, and other interactive and/or networked electronic applications. Some of the items (e.g. all subitems under item 5 - description of the intervention) may also be applicable for other study designs.

The goal of the CONSORT EHEALTH checklist and guideline is to be
a) a guide for reporting for authors of RCTs,
b) to form a basis for appraisal of an ehealth trial (in terms of validity)

CONSORT-EHEALTH items/subitems are MANDATORY reporting items for studies published in the Journal of Medical Internet Research and other journals / scientific societies endorsing the checklist.

Items numbered 1., 2., 3., 4a., 4b etc are original CONSORT or CONSORT-NPT (non-pharmacologic treatment) items.
Items with Roman numerals (i., ii, iii, iv etc.) are CONSORT-EHEALTH extensions/clarifications.

As the CONSORT-EHEALTH checklist is still considered in a formative stage, we would ask that you also RATE ON A SCALE OF 1-5 how important/useful you feel each item is FOR THE PURPOSE OF THE CHECKLIST and reporting guideline (optional).

Mandatory reporting items are marked with a red *.
In the textboxes, either copy & paste the relevant sections from your manuscript into this form - please include any quotes from your manuscript in QUOTATION MARKS,
or answer directly by providing additional information not in the manuscript, or elaborating on why the item was not relevant for this study.

YOUR ANSWERS WILL BE PUBLISHED AS A SUPPLEMENTARY FILE TO YOUR PUBLICATION IN JMIR AND ARE CONSIDERED PART OF YOUR PUBLICATION (IF ACCEPTED).
Please fill in these questions diligently. Information will not be copyedited, so please use proper spelling and grammar, use correct capitalization, and avoid abbreviations.

DO NOT FORGET TO SAVE AS PDF _AND_ CLICK THE SUBMIT BUTTON SO YOUR ANSWERS ARE IN OUR DATABASE !!!

Citation Suggestion (if you append the pdf as Appendix we suggest to cite this paper in the caption):
Eysenbach G, CONSORT-EHEALTH Group
CONSORT-EHEALTH: Improving and Standardizing Evaluation Reports of Web-based and Mobile Health Interventions
J Med Internet Res 2011;13(4):e126

URL: http://www.jmir.org/2011/4/e126/
doi: 10.2196/jmir.1923
PMID: 22209829

登录 Google 即可保存进度。了解详情

* 表示必填

Your name *

First Last

TongWu

Primary Affiliation (short), City, Country *

University of Toronto, Toronto, Canada

Shanghaijiaotong University of Shanghai,China

Your e-mail address *

abc@gmail.com

mumumumu@sjtu.edu.cn

Title of your manuscript *

Provide the (draft) title of your manuscript.

Telemedicine Application for Non-Rigid Facial Rehabilitation Training Enhanced by EffiFCNN-ResNet to Improve Accessibility for Nasopharyngeal Carcinoma Cancer Patients：A Randomized Controlled Trial

Name of your App/Software/Intervention *

If there is a short and a long/alternate name, write the short name first and add the long name in brackets.

Opencare

Evaluated Version (if any)

e.g. "V1", "Release 2017-03-01", "Version 2.0.27913"

您的回答

Language(s) *

What language is the intervention/app in? If multiple languages are available, separate by comma (e.g. "English, French")

Chinese,English

URL of your Intervention Website or App

e.g. a direct link to the mobile app on app in appstore (itunes, Google Play), or URL of the website. If the intervention is a DVD or hardware, you can also link to an Amazon page.

您的回答

URL of an image/screenshot (optional)

您的回答

Accessibility *

Can an enduser access the intervention presently?

- ○ access is free and open
- ◉ access only for special usergroups, not open
- ○ access is open to everyone, but requires payment/subscription/in-app purchases
- ○ app/intervention no longer accessible
- ○ 其他:

Primary Medical Indication/Disease/Condition *

e.g. "Stress", "Diabetes", or define the target group in brackets after the condition, e.g. "Autism (Parents of children with)", "Alzheimers (Informal Caregivers of)"

Nasopharyngeal Carcinoma Cancer Patients

Primary Outcomes measured in trial *

comma-separated list of primary outcomes reported in the trial

Maximum mouth opening, mouth opening sym

Secondary/other outcomes

Are there any other outcomes the intervention is expected to affect?

Fatigue (Brief Fatigue Inventory), health-related quality of life (AQoL-6D), and system usability (SUS).

Recommended "Dose" *

What do the instructions for users say on how often the app should be used?

- (●) Approximately Daily
- ( ) Approximately Weekly
- ( ) Approximately Monthly
- ( ) Approximately Yearly
- ( ) "as needed"
- ( ) 其他:

Approx. Percentage of Users (starters) still using the app as recommended after 3 months *

- ( ) unknown / not evaluated
- ( ) 0-10%
- ( ) 11-20%
- ( ) 21-30%
- ( ) 31-40%
- ( ) 41-50%
- ( ) 51-60%
- ( ) 61-70%
- ( ) 71%-80%
- ( ) 81-90%
- (●) 91-100%
- ( ) 其他:

Overall, was the app/intervention effective? *

- ( ) yes: all primary outcomes were significantly better in intervention group vs control
- (•) partly: SOME primary outcomes were significantly better in intervention group vs control
- ( ) no statistically significant difference between control and intervention
- ( ) potentially harmful: control was significantly better than intervention in one or more outcomes
- ( ) inconclusive: more research is needed
- ( ) 其他:

Article Preparation Status/Stage *

At which stage in your article preparation are you currently (at the time you fill in this form)

- ( ) not submitted yet - in early draft status
- ( ) not submitted yet - in late draft status, just before submission
- (•) submitted to a journal but not reviewed yet
- ( ) submitted to a journal and after receiving initial reviewer comments
- ( ) submitted to a journal and accepted, but not published yet
- ( ) published
- ( ) 其他:

Journal *

If you already know where you will submit this paper (or if it is already submitted), please provide the journal name (if it is not JMIR, provide the journal name under "other")

- ○ not submitted yet / unclear where I will submit this
- ○ Journal of Medical Internet Research (JMIR)
- ◉ JMIR mHealth and UHealth
- ○ JMIR Serious Games
- ○ JMIR Mental Health
- ○ JMIR Public Health
- ○ JMIR Formative Research
- ○ Other JMIR sister journal
- ○ 其他:

Is this a full powered effectiveness trial or a pilot/feasibility trial? *

- ○ Pilot/feasibility
- ◉ Fully powered

Manuscript tracking number *

If this is a JMIR submission, please provide the manuscript tracking number under "other" (The ms tracking number can be found in the submission acknowledgement email, or when you login as author in JMIR. If the paper is already published in JMIR, then the ms tracking number is the four-digit number at the end of the DOI, to be found at the bottom of each published article in JMIR)

- ○ no ms number (yet) / not (yet) submitted to / published in JMIR
- ◉ 其他: 72560

TITLE AND ABSTRACT

1a) TITLE: Identification as a randomized trial in the title

1a) Does your paper address CONSORT item 1a? *

I.e does the title contain the phrase "Randomized Controlled Trial"? (if not, explain the reason under "other")

- (selected) yes
- 其他:

1a-i) Identify the mode of delivery in the title

Identify the mode of delivery. Preferably use “web-based” and/or “mobile” and/or “electronic game” in the title. Avoid ambiguous terms like “online”, “virtual”, “interactive”. Use “Internet-based” only if Intervention includes non-web-based Internet components (e.g. email), use “computer-based” or “electronic” only if offline products are used. Use “virtual” only in the context of “virtual reality” (3-D worlds). Use “online” only in the context of “online support groups”. Complement or substitute product names with broader terms for the class of products (such as “mobile” or “smart phone” instead of “iphone”), especially if the application runs on different platforms.

	1	2	3	4	5	
subitem not at all important	○	○	●	○	○	essential

清除所选内容

Does your paper address subitem 1a-i? *

Copy and paste relevant sections from manuscript title (include quotes in quotation marks "like this" to indicate direct quotes from your manuscript), or elaborate on this item by providing additional information not in the ms, or briefly explain why the item is not applicable/relevant for your study

Telemedicine Application for Non-Rigid Facial Rehabilitation Training Enhanced by EffiFCNN-ResNet to Improve Accessibility for Nasopharyngeal Carcinoma Cancer Patients：A Randomized Controlled Trial，"Telemedicine Application" like this "mobile"

1a-ii) Non-web-based components or important co-interventions in title

Mention non-web-based components or important co-interventions in title, if any (e.g., “with telephone support”).

	1	2	3	4	5	
subitem not at all important	○	○	◉	○	○	essential

清除所选内容

Does your paper address subitem 1a-ii?

Copy and paste relevant sections from manuscript title (include quotes in quotation marks "like this" to indicate direct quotes from your manuscript), or elaborate on this item by providing additional information not in the ms, or briefly explain why the item is not applicable/relevant for your study

您的回答

1a-iii) Primary condition or target group in the title

Mention primary condition or target group in the title, if any (e.g., “for children with Type I Diabetes”) Example: A Web-based and Mobile Intervention with Telephone Support for Children with Type I Diabetes: Randomized Controlled Trial

	1	2	3	4	5	
subitem not at all important	○	○	○	○	◉	essential

清除所选内容

Does your paper address subitem 1a-iii? *

Copy and paste relevant sections from manuscript title (include quotes in quotation marks "like this" to indicate direct quotes from your manuscript), or elaborate on this item by providing additional information not in the ms, or briefly explain why the item is not applicable/relevant for your study

Telemedicine Application for Non-Rigid Facial Rehabilitation Training Enhanced by EffiFCNN-ResNet to Improve Accessibility for Nasopharyngeal Carcinoma Cancer Patients：A Randomized Controlled Trial,"Nasopharyngeal Carcinoma Cancer Patients" like this "Nasopharyngeal Carcinoma Cancer Mention primary condition or target group"

1b) ABSTRACT: Structured summary of trial design, methods, results, and conclusions

NPT extension: Description of experimental treatment, comparator, care providers, centers, and blinding status.

1b-i) Key features/functionalities/components of the intervention and comparator in the METHODS section of the ABSTRACT

Mention key features/functionalities/components of the intervention and comparator in the abstract. If possible, also mention theories and principles used for designing the site. Keep in mind the needs of systematic reviewers and indexers by including important synonyms. (Note: Only report in the abstract what the main paper is reporting. If this information is missing from the main body of text, consider adding it)

	1	2	3	4	5	
subitem not at all important	○	○	○	○	◉	essential

清除所选内容

Does your paper address subitem 1b-i? *

Copy and paste relevant sections from the manuscript abstract (include quotes in quotation marks "like this" to indicate direct quotes from your manuscript), or elaborate on this item by providing additional information not in the ms, or briefly explain why the item is not applicable/relevant for your study

Like this"A parallel-group, two-arm randomized controlled trial was conducted with 108 patients, randomly assigned to either the intervention group (n = 54) or the control group (n = 54).The intervention group performed mouth-opening exercises under the supervision and guidance of the telemedicine application, while the control group followed traditional video-based instructions.Primary outcome measures included maximum mouth opening, mouth opening symmetry, exercise frequency, and rehabilitation-related health beliefs. Secondary outcomes included fatigue (Brief Fatigue Inventory), health-related quality of life (AQoL-6D), and system usability (SUS). Data were analyzed using T-tests, chi-square tests, and Mann-Whitney U tests."

1b-ii) Level of human involvement in the METHODS section of the ABSTRACT

Clarify the level of human involvement in the abstract, e.g., use phrases like “fully automated” vs. “therapist/nurse/care provider/physician-assisted” (mention number and expertise of providers involved, if any). (Note: Only report in the abstract what the main paper is reporting. If this information is missing from the main body of text, consider adding it)

	1	2	3	4	5	
subitem not at all important	○	○	○	○	◉	essential

清除所选内容

Does your paper address subitem 1b-ii?

Copy and paste relevant sections from the manuscript abstract (include quotes in quotation marks "like this" to indicate direct quotes from your manuscript), or elaborate on this item by providing additional information not in the ms, or briefly explain why the item is not applicable/relevant for your study

Like this"A parallel-group, two-arm randomized controlled trial was conducted with 108 patients, randomly assigned to either the intervention group (n = 54) or the control group (n = 54).The intervention group performed mouth-opening exercises under the supervision and guidance of the telemedicine application, while the control group followed traditional video-based instructions."

1b-iii) Open vs. closed, web-based (self-assessment) vs. face-to-face assessments in the METHODS section of the ABSTRACT

Mention how participants were recruited (online vs. offline), e.g., from an open access website or from a clinic or a closed online user group (closed usergroup trial), and clarify if this was a purely web-based trial, or there were face-to-face components (as part of the intervention or for assessment). Clearly say if outcomes were self-assessed through questionnaires (as common in web-based trials). Note: In traditional offline trials, an open trial (open-label trial) is a type of clinical trial in which both the researchers and participants know which treatment is being administered. To avoid confusion, use “blinded” or “unblinded” to indicated the level of blinding instead of “open”, as “open” in web-based trials usually refers to “open access” (i.e. participants can self-enrol). (Note: Only report in the abstract what the main paper is reporting. If this information is missing from the main body of text, consider adding it)

	1	2	3	4	5	
subitem not at all important	○	◉	○	○	○	essential

清除所选内容

Does your paper address subitem 1b-iii?

Copy and paste relevant sections from the manuscript abstract (include quotes in quotation marks "like this" to indicate direct quotes from your manuscript), or elaborate on this item by providing additional information not in the ms, or briefly explain why the item is not applicable/relevant for your study

您的回答

1b-iv) RESULTS section in abstract must contain use data

Report number of participants enrolled/assessed in each group, the use/uptake of the intervention (e.g., attrition/adherence metrics, use over time, number of logins etc.), in addition to primary/secondary outcomes. (Note: Only report in the abstract what the main paper is reporting. If this information is missing from the main body of text, consider adding it)

	1	2	3	4	5	
subitem not at all important	○	○	○	○	◉	essential

清除所选内容

Does your paper address subitem 1b-iv?

Copy and paste relevant sections from the manuscript abstract (include quotes in quotation marks "like this" to indicate direct quotes from your manuscript), or elaborate on this item by providing additional information not in the ms, or briefly explain why the item is not applicable/relevant for your study

Like this "A parallel-group, two-arm randomized controlled trial was conducted with 108 patients, randomly assigned to either the intervention group (n = 54) or the control group (n = 54).The intervention group performed mouth-opening exercises under the supervision and guidance of the telemedicine application, while the control group followed traditional video-based instructions.Primary outcome measures included maximum mouth opening, mouth opening symmetry, exercise frequency, and rehabilitation-related health beliefs. Secondary outcomes included fatigue (Brief Fatigue Inventory), health-related quality of life (AQoL-6D), and system usability (SUS). Data were analyzed using T-tests, chi-square tests, and Mann-Whitney U tests."

1b-v) CONCLUSIONS/DISCUSSION in abstract for negative trials

Conclusions/Discussions in abstract for negative trials: Discuss the primary outcome - if the trial is negative (primary outcome not changed), and the intervention was not used, discuss whether negative results are attributable to lack of uptake and discuss reasons. (Note: Only report in the abstract what the main paper is reporting. If this information is missing from the main body of text, consider adding it)

	1	2	3	4	5	
subitem not at all important	●	○	○	○	○	essential

清除所选内容

Does your paper address subitem 1b-v?

Copy and paste relevant sections from the manuscript abstract (include quotes in quotation marks "like this" to indicate direct quotes from your manuscript), or elaborate on this item by providing additional information not in the ms, or briefly explain why the item is not applicable/relevant for your study

您的回答

INTRODUCTION

2a) In INTRODUCTION: Scientific background and explanation of rationale

2a-i) Problem and the type of system/solution

Describe the problem and the type of system/solution that is object of the study: intended as stand-alone intervention vs. incorporated in broader health care program? Intended for a particular patient population? Goals of the intervention, e.g., being more cost-effective to other interventions, replace or complement other solutions? (Note: Details about the intervention are provided in “Methods” under 5)

	1	2	3	4	5	
subitem not at all important	○	○	○	○	◉	essential

清除所选内容

Does your paper address subitem 2a-i? *

Copy and paste relevant sections from the manuscript (include quotes in quotation marks "like this" to indicate direct quotes from your manuscript), or elaborate on this item by providing additional information not in the ms, or briefly explain why the item is not applicable/relevant for your study

Like this"Results
Results showed significant improvements in the intervention group in maximum mouth opening, exercise frequency, perceived benefits, self-efficacy, and action cues ($p < 0.05$). The system demonstrated 98.2% accuracy in assessing facial training exercises. Participants also reported favorable training experiences.
Conclusions
This telemedicine approach was more effective than traditional methods, improving patient engagement and rehabilitation outcomes, while providing a more objective and precise monitoring tool. Future applications may benefit NPC and other head and neck cancer patients."

2a-ii) Scientific background, rationale: What is known about the (type of) system

Scientific background, rationale: What is known about the (type of) system that is the object of the study (be sure to discuss the use of similar systems for other conditions/diagnoses, if appropiate), motivation for the study, i.e. what are the reasons for and what is the context for this specific study, from which stakeholder viewpoint is the study performed, potential impact of findings [2]. Briefly justify the choice of the comparator.

	1	2	3	4	5	
subitem not at all important	○	○	○	○	◉	essential

清除所选内容

Does your paper address subitem 2a-ii? *

Copy and paste relevant sections from the manuscript (include quotes in quotation marks "like this" to indicate direct quotes from your manuscript), or elaborate on this item by providing additional information not in the ms, or briefly explain why the item is not applicable/relevant for your study

Like this"Nasopharyngeal carcinoma (NPC) is a common malignant tumor of the head and neck, with a high prevalence in Eastern and Southeast Asian countries. Comprehensive treatment, primarily based on radiotherapy, is the preferred therapeutic approach for NPC [1]. Trismus is one of the common complications of radiotherapy, with an incidence ranging from 12.0% to 58.5%, significantly affecting patients' quality of life[2]. Current research on mouth-opening training and intervention for NPC patients primarily focuses on the content, timing, methods, and tools of such exercises. Traditional clinical interventions commonly employ cork resistance training; however, patients often experience significant pain perception[3]. To address this issue, Zhang et al.proposed a novel therapy combining hardware-assisted passive mouth-opening traction with transcutaneous neuromuscular electrical stimulation[4]. However, contact-based training may exacerbate symptoms in patients suffering from radiation-induced dermatitis or skin fibrosis[5]. Therefore, there is an urgent need to explore alternative approaches, particularly non-contact, visually interactive supportive interventions for NPC patients post-radiotherapy, to improve their prognosis.

Studies have demonstrated that early implementation of active mouth-opening exercises can effectively prevent the onset of trismus[6, 7]. Moreover, Samarah et al. confirmed that targeted mouth-opening training interventions play a significant role in reducing the incidence of trismus[8], improving patient adherence, and enhancing quality of life in NPC patients undergoing radiotherapy and chemotherapy[9]. Despite the proven efficacy of mouth-opening exercises, long-term treatment outcomes remain inconsistent[10], likely due to the high healthcare burden of training, movement correction, and supervision[11].

Digital health interventions can provide scalable health education and self-management support for NPC patients through applications or web-based platforms[12], offering high coverage, low cost, and easy accessibility to mitigate constraints related to time and resources [13-16]. However, the effectiveness of some digital interventions exhibits variability. For instance, while physiotherapy programs and online applications have demonstrated efficacy in improving physical function [17] ,their long-term effectiveness declines due to a lack of precise[18], individualized support. Additionally, a physical activity counseling program based on wearable devices reported no prognostic benefits, as it lacked accurate monitoring and feedback on training movements[19, 20].This limitation diminishes the effectiveness of telemedicine applications in facilitating patient rehabilitation behaviors. A meta-analysis further highlighted that precise movement monitoring and targeted feedback are critical factors in enhancing the effectiveness of digital health interventions for patient health management. Therefore, future digital health interventions should prioritize advancements in personalized, intelligent, and real-time monitoring and feedback technologies to enhance intervention efficacy[21, 22].

In clinical practice, the evaluation of jaw training for patients with limited mouth opening mainly relies on direct observation and manual measurements[23], which have limitations in data objectivity and real-time applicability. Recently, machine learning has shown promise in intelligent assessment and quantitative rehabilitation, providing objective monitoring for clinical care[23].However, NPC patients undergoing radiotherapy may experience skin fibrosis, inflammation, swelling, and mandibular deformities, complicating facial motion analysis. Additionally, factors such as head movement and lighting variations exacerbate non-rigid facial deformations, making accurate motion tracking difficult[24]. Existing methods relying on contact-based markers are prone to facial trauma, patient discomfort, and insufficient real-time tracking accuracy.Thus, developing a deep learning-based, non-contact, real-time monitoring system is crucial for enhancing NPC rehabilitation[25].Recent advancements in artificial intelligence have enabled machines to automatically analyze and interpret complex data, supporting more personalized treatment strategies. For instance,Ding et al. explored dense facial tracking sequences using CNN networks combined with non-rigid ICP algorithms to address frame-to-frame relationships in 3D facial analysis[26]. Honey et al., employed a hybrid deep learning algorithm, including MtCNN and

[illegible]
DeepFace, to overcome non-rigid facial changes, such as variations in size, shape, and color [27].Bandaru et al. proposed the Tiefes FCNN model, achieving high accuracy in microexpression recognition for non-rigid facial movements [28, 29], demonstrating the feasibility of CNN-based deep networks.
To address the challenges in rehabilitation training for nasopharyngeal carcinoma (NPC) patients, we present Open Care, a remote healthcare application designed specifically for NPC jaw rehabilitation(Figure 1). By utilizing an EffiFCNN-ResNet model, Open Care enables non-contact, real-time facial motion tracking, measurement, and automated feedback. This system offers a novel solution for personalized, efficient NPC rehabilitation. A randomized controlled trial will evaluate its effectiveness in improving patient adherence and rehabilitation outcomes. This work is innovative as no previous study has integrated deep learning-based non-rigid facial recognition with personalized monitoring and feedback for NPC mouth-opening rehabilitation.Our main contributions are as follows:
- A lightweight, deep learning-based non-rigid facial motion real-time tracking and feedback system, specifically designed for nasopharyngeal carcinoma (NPC) patients in remote healthcare applications.
- A reliable intervention method for mouth-opening training, whose effectiveness was validated through randomized controlled trials, with positive feedback from both patients and experts during interviews.
- A reliable and objective intelligent assessment method for mouth-opening rehabilitation, achieving a measurement accuracy of 98.2%, recognized by clinical experts.
- The introduction of an enhanced deep model based on EffiFCNN-ResNet and a patient rehabilitation training dataset, contributing valuable resources to the field of rehabilitation medical research."

2b) In INTRODUCTION: Specific objectives or hypotheses

Does your paper address CONSORT subitem 2b? *

Copy and paste relevant sections from the manuscript (include quotes in quotation marks "like this" to indicate direct quotes from your manuscript), or elaborate on this item by providing additional information not in the ms, or briefly explain why the item is not applicable/relevant for your study

Like this"To address the challenges in rehabilitation training for nasopharyngeal carcinoma (NPC) patients, we present Open Care, a remote healthcare application designed specifically for NPC jaw rehabilitation(Figure 1). By utilizing an EffiFCNN-ResNet model, Open Care enables non-contact, real-time facial motion tracking, measurement, and automated feedback. This system offers a novel solution for personalized, efficient NPC rehabilitation. A randomized controlled trial will evaluate its effectiveness in improving patient adherence and rehabilitation outcomes. This work is innovative as no previous study has integrated deep learning-based non-rigid facial recognition with personalized monitoring and feedback for NPC mouth-opening rehabilitation.Our main contributions are as follows:
A lightweight, deep learning-based non-rigid facial motion real-time tracking and feedback system, specifically designed for nasopharyngeal carcinoma (NPC) patients in remote healthcare applications.
A reliable intervention method for mouth-opening training, whose effectiveness was validated through randomized controlled trials, with positive feedback from both patients and experts during interviews.
A reliable and objective intelligent assessment method for mouth-opening rehabilitation, achieving a measurement accuracy of 98.2%, recognized by clinical experts.
The introduction of an enhanced deep model based on EffiFCNN-ResNet and a patient rehabilitation training dataset, contributing valuable resources to the field of rehabilitation medical research."

METHODS

3a) Description of trial design (such as parallel, factorial) including allocation ratio

Does your paper address CONSORT subitem 3a? *

Copy and paste relevant sections from the manuscript (include quotes in quotation marks "like this" to indicate direct quotes from your manuscript), or elaborate on this item by providing additional information not in the ms, or briefly explain why the item is not applicable/relevant for your study

Like this"A randomized, parallel-controlled, two-arm, four-week study was conducted at an oncology hospital. In a 1:1 ratio, a cohort of 108 eligible participants were assigned at random to either the usual care control group or the app intervention group. The primary and secondary outcomes were evaluated at the time of enrollment and four weeks later. Each and every data collector was not informed of the group assignment. Prior to their involvement, participants in this research study provided written informed consent, and the trial received approval from the human research ethics committee of the institution (H20240007I).SPSS version 26.0 was utilized to analyze the data. For quantitative measurements involving categorical variables, descriptive data are utilized in the form of means (M) (accompanied by standard deviations SD) or numbers (N) (with percentages). In order to compare the baseline characteristics of participants who contributed the main results with those who did not, a T-test or chi-square test was employed. In light of the variable distribution, the Mann-Whitney U test was chosen to assess the significance of differences in the data between and within groups prior to and following the intervention test. A comparison was made between the control group and the intervention group in terms of their differences. For all statistical analyses, inter-group and intra-group differences were expressed as the mean and 95% confidence interval (CI). At 0.05, the significance level (alpha) was established.A significance level of 0.05 or lower was employed to classify a difference as statistically significant."

3b) Important changes to methods after trial commencement (such as eligibility criteria), with reasons

Does your paper address CONSORT subitem 3b? *

Copy and paste relevant sections from the manuscript (include quotes in quotation marks "like this" to indicate direct quotes from your manuscript), or elaborate on this item by providing additional information not in the ms, or briefly explain why the item is not applicable/relevant for your study

Like this"3.1.2 Individuals Involved
The study participants comprised nasopharyngeal cancer patients who had finished radiotherapy and were prepared to be discharged from the oncology facility. They were recruited via posters and questionnaires.
The following were the criteria for inclusion:(1) Participants were those who had received a pathology or histological diagnosis of nasopharyngeal cancer and were prescribed radiotherapy. (2) Patients were between the ages of 18 and 65; (3) Patients were cognizant of their condition; (4) Patients gave informed consent and agreed to participate in this study; (5) Patients had access to smartphones and WeChat applications; and (6) Patients had Functional Status Scores (KPS) ranging from 80 to 100[35] and Eastern Cancer Cooperative Group (ECOG) scores ranging from 0 to 5[36].
The criteria for exclusion were as follows: (1) Individuals who have a history of radiotherapy; (2) Individuals who are unable to engage in physical activity due to underlying cardiac, neurological, muscular, or joint disease; and (3) Individuals who have undergone pertinent mouth-opening exercises (as part of their daily care, follow-up videos, etc.).
The criteria for withdrawal were as follows: (1) lack of desire to proceed with the trial; (2) onset of a severe illness that hindered trial continuation; (3) adverse event associated with open-mouth exercise or regular physical activity; and (4) Additional surgical interventions, such as mandibular resection."

3b-i) Bug fixes, Downtimes, Content Changes

Bug fixes, Downtimes, Content Changes: ehealth systems are often dynamic systems. A description of changes to methods therefore also includes important changes made on the intervention or comparator during the trial (e.g., major bug fixes or changes in the functionality or content) (5-iii) and other “unexpected events” that may have influenced study design such as staff changes, system failures/downtimes, etc. [2].

	1	2	3	4	5	
subitem not at all important	◉	○	○	○	○	essential

清除所选内容

Does your paper address subitem 3b-i?

Copy and paste relevant sections from the manuscript (include quotes in quotation marks "like this" to indicate direct quotes from your manuscript), or elaborate on this item by providing additional information not in the ms, or briefly explain why the item is not applicable/relevant for your study

您的回答

4a) Eligibility criteria for participants

Does your paper address CONSORT subitem 4a? *

Copy and paste relevant sections from the manuscript (include quotes in quotation marks "like this" to indicate direct quotes from your manuscript), or elaborate on this item by providing additional information not in the ms, or briefly explain why the item is not applicable/relevant for your study

like this"3.1.2 Individuals Involved
The study participants comprised nasopharyngeal cancer patients who had finished radiotherapy and were prepared to be discharged from the oncology facility. They were recruited via posters and questionnaires.
The following were the criteria for inclusion:(1) Participants were those who had received a pathology or histological diagnosis of nasopharyngeal cancer and were prescribed radiotherapy. (2) Patients were between the ages of 18 and 65; (3) Patients were cognizant of their condition; (4) Patients gave informed consent and agreed to participate in this study; (5) Patients had access to smartphones and WeChat applications; and (6) Patients had Functional Status Scores (KPS) ranging from 80 to 100[35] and Eastern Cancer Cooperative Group (ECOG) scores ranging from 0 to 5[36].
The criteria for exclusion were as follows: (1) Individuals who have a history of radiotherapy; (2) Individuals who are unable to engage in physical activity due to underlying cardiac, neurological, muscular, or joint disease; and (3) Individuals who have undergone pertinent mouth-opening exercises (as part of their daily care, follow-up videos, etc.).
The criteria for withdrawal were as follows: (1) lack of desire to proceed with the trial; (2) onset of a severe illness that hindered trial continuation; (3) adverse event associated with open-mouth exercise or regular physical activity; and (4) Additional surgical interventions, such as mandibular resection."

4a-i) Computer / Internet literacy

Computer / Internet literacy is often an implicit “de facto” eligibility criterion - this should be explicitly clarified.

	1	2	3	4	5	
subitem not at all important	○	◉	○	○	○	essential

清除所选内容

Does your paper address subitem 4a-i?

Copy and paste relevant sections from the manuscript (include quotes in quotation marks "like this" to indicate direct quotes from your manuscript), or elaborate on this item by providing additional information not in the ms, or briefly explain why the item is not applicable/relevant for your study

您的回答

4a-ii) Open vs. closed, web-based vs. face-to-face assessments:

Open vs. closed, web-based vs. face-to-face assessments: Mention how participants were recruited (online vs. offline), e.g., from an open access website or from a clinic, and clarify if this was a purely web-based trial, or there were face-to-face components (as part of the intervention or for assessment), i.e., to what degree got the study team to know the participant. In online-only trials, clarify if participants were quasi-anonymous and whether having multiple identities was possible or whether technical or logistical measures (e.g., cookies, email confirmation, phone calls) were used to detect/prevent these.

	1	2	3	4	5	
subitem not at all important	○	○	○	○	◉	essential

清除所选内容

Does your paper address subitem 4a-ii? *

Copy and paste relevant sections from the manuscript (include quotes in quotation marks "like this" to indicate direct quotes from your manuscript), or elaborate on this item by providing additional information not in the ms, or briefly explain why the item is not applicable/relevant for your study

Like this"Through the implementation of sealed opaque envelopes that were disseminated by other researchers, we effectively concealed the allocation information from the result evaluators. Participants were strongly encouraged to refrain from disclosing such details during the process of calculating the results."

4a-iii) Information giving during recruitment

Information given during recruitment. Specify how participants were briefed for recruitment and in the informed consent procedures (e.g., publish the informed consent documentation as appendix, see also item X26), as this information may have an effect on user self-selection, user expectation and may also bias results.

	1	2	3	4	5	
subitem not at all important	○	○	○	○	◉	essential

清除所选内容

Does your paper address subitem 4a-iii?

Copy and paste relevant sections from the manuscript (include quotes in quotation marks "like this" to indicate direct quotes from your manuscript), or elaborate on this item by providing additional information not in the ms, or briefly explain why the item is not applicable/relevant for your study

您的回答

4b) Settings and locations where the data were collected

Does your paper address CONSORT subitem 4b? *

Copy and paste relevant sections from the manuscript (include quotes in quotation marks "like this" to indicate direct quotes from your manuscript), or elaborate on this item by providing additional information not in the ms, or briefly explain why the item is not applicable/relevant for your study

Like this"Before beginning the intervention, gather disease-related information (cancer type, treatment method, course of disease, number of mouth opening recovery exercises per week, and household residence), education level, name, gender, age, and education level of all participants. Conduct baseline measurements on these individuals one week prior to the commencement of the intervention, focusing on their maximum mouth opening and other scores. In order to determine the individual's maximum opening range, the user must expand the aperture as gradually as feasible throughout the measurement. A three-second pause is reinstated, and five data points are collected during the middle second in order to compute the average and conclude the maximal opening measurement. Following that, they were separated into an experimental group and a control group based on age and gender, with 54 individuals in each group; thus, the age and gender distributions of the two groups were comparable. This is because, to achieve balance, other characteristics in the control group and the experimental group should remain constant throughout the duration of the study, in addition to the research factor of treatment receipt."

4b-i) Report if outcomes were (self-)assessed through online questionnaires

Clearly report if outcomes were (self-)assessed through online questionnaires (as common in web-based trials) or otherwise.

	1	2	3	4	5	
subitem not at all important	○	○	○	○	◉	essential

清除所选内容

Does your paper address subitem 4b-i? *

Copy and paste relevant sections from the manuscript (include quotes in quotation marks "like this" to indicate direct quotes from your manuscript), or elaborate on this item by providing additional information not in the ms, or briefly explain why the item is not applicable/relevant for your study

Like this"3.3 Outcomes Measures
A validated online or in-person questionnaire was administered to each participant at baseline and four weeks following the intervention. No monetary compensation or other form of incentive was provided to participants in exchange for their participation in the questionnaire or study. The baseline demographic data collection process was comprehensively detailed in the section devoted to results measurement.
3.3.1 Primary Outcomes
Maximum data on mouth aperture.Through the collection and comparison of the subjects' maximum mouth opening data prior to and subsequent to the intervention, one can assess and analyze the impact of the intervention application on the mouth opening function. Subsequently, e oduct's effectiveness in enhancing the mouth opening function can be finalized.
Evaluation of the symmetry of the mouth aperture.By documenting and contrasting the symmetry of the subjects' oral movements prior to and subsequent to the intervention, one can facilitate the assessment of the product's impact on oral function.
Number of exercises per week.The behavior's implementation was quantitatively assessed by comparing and recording the weekly training durations of the participants in the healthy behavior prior to and following the intervention.
HBMQ Inquiry Form. Extensively utilized for the assessment of health behaviors[37].Six aspects of HBM were addressed in the HBMQ devised by Kasmaei et al. (2014),: perceived susceptibility (three items), perceived severity (seven items), perceived benefits (three items), perceived barriers (seven items), self-efficacy (five items), action cues (three items), and behavior (three items)[38]. The responses for all subscale items, with the exception of the behavior subscale, spanned from completely disagree (0) to completely concur (4). In each of the six domains, participants were requested to rate sentences that reflected their beliefs. With optimal psychological measurement characteristics, the behavior subscale requests the frequency of behavior, such as the number of mouth-opening exercises, on a scale from never (0) to always (4).
4.3.2 Secondary Cognitive Outcomes
Borg Rating of Perceived Exertion Scale (BRPE)[39] .Self-rated physical exertion during exercise is quantified using the BRPE, which assigns values between 6 (indicating no exercise intensity) and 20 (representing maximal exercise intensity).
AQoL-6D.The health-related quality of life is quantified using the [40] version of the quality of life assessment tool. A score between -0.04 and 1.00 indicates a superior quality of life.
System Usability Scale (SUS) .Perceived availability is assessed utilizing the system availability scale. Upon successful completion of the designated content assignment, participants have the ability to promptly obtain their scores using SUS[41]. A larger score signifies enhanced system availability of the product."

4b-ii) Report how institutional affiliations are displayed

Report how institutional affiliations are displayed to potential participants [on ehealth media], as affiliations with prestigious hospitals or universities may affect volunteer rates, use, and reactions with regards to an intervention.(Not a required item – describe only if this may bias results)

	1	2	3	4	5	
subitem not at all important	○	○	○	◉	○	essential

清除所选内容

Does your paper address subitem 4b-ii?

Copy and paste relevant sections from the manuscript (include quotes in quotation marks "like this" to indicate direct quotes from your manuscript), or elaborate on this item by providing additional information not in the ms, or briefly explain why the item is not applicable/relevant for your study

您的回答

5) The interventions for each group with sufficient details to allow replication, including how and when they were actually administered

5-i) Mention names, credential, affiliations of the developers, sponsors, and owners

Mention names, credential, affiliations of the developers, sponsors, and owners [6] (if authors/evaluators are owners or developer of the software, this needs to be declared in a “Conflict of interest” section or mentioned elsewhere in the manuscript).

	1	2	3	4	5	
subitem not at all important	○	○	○	◉	○	essential

清除所选内容

Does your paper address subitem 5-i?

Copy and paste relevant sections from the manuscript (include quotes in quotation marks "like this" to indicate direct quotes from your manuscript), or elaborate on this item by providing additional information not in the ms, or briefly explain why the item is not applicable/relevant for your study

您的回答

5-ii) Describe the history/development process

Describe the history/development process of the application and previous formative evaluations (e.g., focus groups, usability testing), as these will have an impact on adoption/use rates and help with interpreting results.

	1	2	3	4	5	
subitem not at all important	○	○	○	○	◉	essential

清除所选内容

Does your paper address subitem 5-ii?

Copy and paste relevant sections from the manuscript (include quotes in quotation marks "like this" to indicate direct quotes from your manuscript), or elaborate on this item by providing additional information not in the ms, or briefly explain why the item is not applicable/relevant for your study

您的回答

5-iii) Revisions and updating

Revisions and updating. Clearly mention the date and/or version number of the application/intervention (and comparator, if applicable) evaluated, or describe whether the intervention underwent major changes during the evaluation process, or whether the development and/or content was “frozen” during the trial. Describe dynamic components such as news feeds or changing content which may have an impact on the replicability of the intervention (for unexpected events see item 3b).

	1	2	3	4	5	
subitem not at all important	◉	○	○	○	○	essential

清除所选内容

Does your paper address subitem 5-iii?

Copy and paste relevant sections from the manuscript (include quotes in quotation marks "like this" to indicate direct quotes from your manuscript), or elaborate on this item by providing additional information not in the ms, or briefly explain why the item is not applicable/relevant for your study

您的回答

5-iv) Quality assurance methods

Provide information on quality assurance methods to ensure accuracy and quality of information provided [1], if applicable.

	1	2	3	4	5	
subitem not at all important	○	○	○	○	◉	essential

清除所选内容

Does your paper address subitem 5-iv?

Copy and paste relevant sections from the manuscript (include quotes in quotation marks "like this" to indicate direct quotes from your manuscript), or elaborate on this item by providing additional information not in the ms, or briefly explain why the item is not applicable/relevant for your study

您的回答

5-v) Ensure replicability by publishing the source code, and/or providing screenshots/screen-capture video, and/or providing flowcharts of the algorithms used

Ensure replicability by publishing the source code, and/or providing screenshots/screen-capture video, and/or providing flowcharts of the algorithms used. Replicability (i.e., other researchers should in principle be able to replicate the study) is a hallmark of scientific reporting.

	1	2	3	4	5	
subitem not at all important	○	○	○	○	◉	essential

清除所选内容

Does your paper address subitem 5-v?

Copy and paste relevant sections from the manuscript (include quotes in quotation marks "like this" to indicate direct quotes from your manuscript), or elaborate on this item by providing additional information not in the ms, or briefly explain why the item is not applicable/relevant for your study

您的回答

5-vi) Digital preservation

Digital preservation: Provide the URL of the application, but as the intervention is likely to change or disappear over the course of the years; also make sure the intervention is archived (Internet Archive, webcitation.org, and/or publishing the source code or screenshots/videos alongside the article). As pages behind login screens cannot be archived, consider creating demo pages which are accessible without login.

	1	2	3	4	5	
subitem not at all important	○	○	○	○	◉	essential

清除所选内容

Does your paper address subitem 5-vi?

Copy and paste relevant sections from the manuscript (include quotes in quotation marks "like this" to indicate direct quotes from your manuscript), or elaborate on this item by providing additional information not in the ms, or briefly explain why the item is not applicable/relevant for your study

您的回答

5-vii) Access

Access: Describe how participants accessed the application, in what setting/context, if they had to pay (or were paid) or not, whether they had to be a member of specific group. If known, describe how participants obtained “access to the platform and Internet” [1]. To ensure access for editors/reviewers/readers, consider to provide a “backdoor” login account or demo mode for reviewers/readers to explore the application (also important for archiving purposes, see vi).

	1	2	3	4	5	
subitem not at all important	○	○	○	○	◉	essential

清除所选内容

Does your paper address subitem 5-vii? *

Copy and paste relevant sections from the manuscript (include quotes in quotation marks "like this" to indicate direct quotes from your manuscript), or elaborate on this item by providing additional information not in the ms, or briefly explain why the item is not applicable/relevant for your study

Like this"3.2.2 Involvement
The control group, consisting of participants receiving conventional care, was informed of the benefits of regular mouth-opening exercises and instructed to maintain their usual lifestyle. The duration and intensity of the exercises were carefully adjusted to avoid discomfort. Researchers provided participants with instructional video demonstrations for the mouth-opening exercises at no cost, along with an application designed to track daily maximum mouth opening, symmetry, and the frequency of weekly video-guided practice.
The video was meticulously designed and recorded by medical professionals from the oncology radiation department, following strict protocols. It was subsequently validated by a multidisciplinary team of experts, including oncologists specializing in head and neck cancer, oral and maxillofacial surgeons, head and neck tumor specialists, rehabilitation physicians, and physical therapists.
In contrast, the intervention group utilized a health application developed by our team, which included the aforementioned instructional videos and a monitoring feedback feature for tracking exercise performance. Throughout the study, all participants were instructed to refrain from engaging in any additional conventional exercises. Researchers conducted weekly phone follow-ups to assess participants' recovery progress and address any inquiries. Furthermore, another research assistant provided detailed instructions on using the application and answered questions in a dedicated chat group. After each training session, the app recorded users' maximum mouth opening and symmetry, while also collecting data on fatigue levels and user experience for subsequent qualitative analysis."

5-viii) Mode of delivery, features/functionalities/components of the intervention and comparator, and the theoretical framework

Describe mode of delivery, features/functionalities/components of the intervention and comparator, and the theoretical framework [6] used to design them (instructional strategy [1], behaviour change techniques, persuasive features, etc., see e.g., [7, 8] for terminology). This includes an in-depth description of the content (including where it is coming from and who developed it) [1]," whether [and how] it is tailored to individual circumstances and allows users to track their progress and receive feedback" [6]. This also includes a description of communication delivery channels and – if computer-mediated communication is a component – whether communication was synchronous or asynchronous [6]. It also includes information on presentation strategies [1], including page design principles, average amount of text on pages, presence of hyperlinks to other resources, etc. [1].

	1	2	3	4	5	
subitem not at all important	○	○	○	○	◉	essential

清除所选内容

Does your paper address subitem 5-viii? *

Copy and paste relevant sections from the manuscript (include quotes in quotation marks "like this" to indicate direct quotes from your manuscript), or elaborate on this item by providing additional information not in the ms, or briefly explain why the item is not applicable/relevant for your study

Like this"2 PROCEDURE
2.1 EffiFCNN-ResNet-based System for Precise Training Monitoring and Assessment
2.1.1 Non-rigid Facial Motion Recognition and Tracking model
We developed the EffiFCNN-ResNet architecture specifically designed to address the complex task of recognizing a large number of non-rigid facial landmarks. Typically, increasing network depth or input image tensors is the most common approach to tackling challenges posed by strong environmental conditions and significant facial structural changes during facial landmark monitoring. However, this often increases computational complexity and execution time. Our backbone network leverages the inherent advantages of FCNN in spatial data processing, incorporating a lightweight EffiResNet Backbone network designed for real-time applications requiring rapid and accurate facial detection and keypoint localization. The underlying principle is rooted in the concept of a single-stage object detection framework, integrating a cleverly designed detector with a lightweight backbone network to achieve fast and accurate facial recognition, as shown in Figure 2. The advantages and contributions of our model are summarized as follows: (1) Custom training on the large-scale public movie dataset AFEW (Acted Facial Expressions in the Wild) using FCNN to detect facial landmarks in complex scene settings[30]. (2) Replacing the FCNN backbone with a rescaled EffiResNet backbone, utilizing large kernel (LK) depthwise (DW) convolutions to expand the output feature map's receptive field (REF)[31]. (3) Nesting the backbone network with channel separation and multi-network landmark detection modules, followed by a linear spatial channel attention module (LAM) combined with Non-Maximum Suppression (NMS) to further optimize and filter features.
Specifically, our model consists of the following components:
(1)Backbone Network: A lightweight Backbone network based on ResNet is employed to extract significant features from input images. The core component is the residual block, which effectively addresses the vanishing gradient problem in deep networks by introducing skip connections, thereby improving training efficiency and model performance. The ResNet architecture exhibits strong hierarchical capability in feature extraction, enabling efficient capture of multi-scale features from low to high levels. Designed based on the Structural Re-parameterization Principle, the backbone network consists of multiple residual blocks, where each block incorporates convolutional layers, batch normalization (BN), and ReLU activation functions. Skip connections allow each layer to directly receive input features from the previous layer, enhancing the stability of the training process. The primary goal is to decouple the multi-branch topology during the training phase from the simplified design during the inference phase. This choice of backbone network strikes a balance between computational efficiency and feature expressiveness, with the architecture proposed to separate learning time multi-branch topology from inference time soft design.
(2)Neck Network: The model integrates the classic Spatial Pyramid Pooling (SPP) and the improved Path Aggregation Network (PAN), designs that enhance the model's feature fusion capabilities and multi-scale perception. The Spatial Pyramid Pooling (SPP) layer performs multi-scale pooling operations to integrate features from different scales, overcoming the limitation of fixed input image sizes, and enabling the capture of multi-scale contextual information to improve the model's ability to perceive target regions. After passing through the SPP, the feature map is transformed into a fixed-length output, providing richer contextual information for the subsequent Path Aggregation Network. The improved Path Aggregation Network (PAN) employs a bottom-up routing fusion strategy, effectively reducing information loss during the transfer of deep-layer features to shallow layers. The Adaptive Feature Pooling (AFP) dynamically integrates feature maps, ensuring that feature information from different levels is fused at all feature levels. This ensures that the features passed to the detection head contain comprehensive and deep semantic information, contributing to improved target detection accuracy.
(3)Anchor Generation Module: This module generates dense anchors (proposed bounding

[illegible] boxes) at different scales and aspect ratios to detect non-rigid facial structures of varying sizes and shapes in the image. It is responsible for merging semantic features from deep layers with texture features from shallow layers, relying on an efficient decoupled head with a loss function.

(4)Face Detection Head and Keypoint Localization Head: The detection head classifies and regresses candidate boxes to optimize their positions and sizes. Meanwhile, the keypoint localization head is responsible for accurately locating facial landmarks, enhancing the algorithm's applicability in tasks requiring detailed facial analysis, thus balancing inference time and accuracy. Dilated Convolution and Basic Block: These are used to enhance the receptive field of feature extraction while preserving spatial resolution.

(5)Multi-Scale Detection: A key feature of the model is its multi-scale detection strategy, which utilizes anchors generated on different feature maps to accommodate facial structures of varying sizes. Concatenate (C) and Upsampling (U): By combining low-level and high-level features through feature fusion and upsampling, the model connects features from different resolutions, ensuring robustness across a wide range of facial scales, which is crucial in real-world deployment scenarios.

(6)Joint Loss Function: FCOS Head: As the final detection head, it combines Focal Loss and Generalized Intersection over Union (GIoU) Loss to achieve more efficient object classification and bounding box regression. The objective of the joint loss function is to simultaneously minimize classification error (facial region classification task) and regression error (bounding box regression and keypoint localization tasks), optimizing the learning process of the network and improving the accuracy of both facial detection and keypoint localization. The deployment method involves inputting the predicted probability labels for each sample and computing the Focal Loss for each sample, then averaging across all samples as the classification loss. GIoU is then computed to evaluate the Intersection over Union (IoU) between the predicted and ground truth bounding boxes, followed by removing the predicted keypoint bounding box errors, calculating squared errors for the true coordinates, and averaging to obtain the keypoint localization loss. Self-monitoring of both losses represents a self-distillation technique, which adjusts knowledge transfer from the teacher model and labels it as the student model during the training phase. The overall loss can be explained as follows:

++ (1)

(7)Real-Time Capture: To enable rapid inference, the model employs multi-scale preprocessing and multi-scale detection, supplemented by optimization techniques such as Non-Maximum Suppression (NMS), making it more suitable for real-time scenarios.Through the architecture described above, FCNN maintains the spatial relationships across the entire network, allowing the generation of dense prediction maps. The lightweight ResNet extends the convolutional layers throughout the network, facilitating a direct correspondence between input pixel positions and their feature representations. Compared to traditional landmark detection, this approach enables the capture of a greater number of facial landmarks with higher accuracy, while also mitigating disturbances in texture and shape caused by non-rigid facial deformations to some extent, as illustrated in Figure 3.For rapid inference, the model adopts a lightweight ResNet-based object detection network enhanced with dilated convolutions. The core architecture employs multi-level dilated convolutions to expand receptive fields and capture contextual information, while integrating a stem module with lightweight pointwise convolutions and hybrid SiLU/ReLU activations to reduce computational complexity. Cross-level feature fusion is achieved through concatenation and upsampling operations to enhance detail retention capabilities. The FCOS-Head detection module addresses class imbalance via focal loss and optimizes bounding box regression using GIoU loss, making it suitable for dense scenarios and small object detection. The unified framework emphasizes three critical aspects: receptive field expansion through hierarchical dilation, multi-scale feature interaction, and lightweight deployment capabilities,

thereby balancing detection accuracy with computational efficiency.

2.1.2 Training Action Classification and Evaluation

Taking the mouth opening action as an example, a more precise evaluation of the motion is achieved through the introduction of a four-level grading system that incorporates both the degree of mouth opening and its symmetry. This grading system simultaneously considers two key aspects: the maximum mouth opening and the symmetry of the mouth shape. The maximum mouth opening is defined as the vertical distance between the highest point of the upper semicircle and the lowest point of the lower semicircle, derived from an elliptical fitting of the mouth region. Symmetry is assessed by the rotation angle of the ellipse.To compute the elliptical equation of the oral region, we employ a facial landmark detection and oral region keypoint elliptical fitting algorithm, based on the aforementioned model, that utilizes over 10,000 keypoints.the algorithm provides an intelligent, automated evaluation of the completion of the mouth opening action for each patient, as illustrated in Figure 4. Through these steps, the keypoints obtained using the deep learning-based facial landmark localization algorithm can be further analyzed to assess the morphological characteristics of the oral region, thereby quantifying the degree of mouth opening and symmetry. The elliptical equation of the oral region can be represented in the following general form:

$$+=1$$

(2)

($x0$, $y0$) is the coordinate of the center of the ellipse, and a and b are the length of the long axis and the short axis of the ellipse, respectively. Subsequently, the vertical distance between the highest point u of the upper semicircle and the lowest point d of the lower semicircle is calculated. The grading assessment criteria are defined by oncological medical experts. The F4 normal range is 35-50 mm, F3 represents mild restriction (25-35 mm), F2 represents moderate restriction (15-25 mm), and F1 represents severe restriction (less than 15 mm).

At the same time, the rotation angle of the ellipse is used to measure the symmetry of the mouth. The rotation angle of the ellipse can be calculated by the following formula.

$$\theta$$

(3)

Here, ϕ is the inclination angle of the ellipse.F4: Rotation angle < 5°: Good symmetry; F3: Rotation angle 5° - 10°: Mild asymmetry; F2: Rotation angle 10° - 15°: Moderate asymmetry; F1: Rotation angle > 15°: Severe asymmetry.

In order to correct the distance between the user and the camera, the two feature points of the inner corner of the eye are connected in a straight line.A standard value of 3.5 cm is set, and a real-time scaling function is derived by calculating the ratio between this reference distance and the actual measured value to perform distance correction, facilitating subsequent data analysis. The corresponding code is shown below,The full implementation is available in supplementary materials.

```
“# Input: Facial landmarks detected via EffiFCNN-ResNet
leftEye = (landmarks.part(39).x, landmarks.part(39).y)  # Left eye corner (point 39)
rightEye = (landmarks.part(42).x, landmarks.part(42).y)  # Right eye corner (point 42)
# Calculate Euclidean distance between eyes
eyeDistance = np.linalg.norm(np.array(leftEye) - np.array(rightEye))  # ||p_left - p_right||_2
# Compute scale factor using reference eye distance
scale = 3.5 / eyeDistance  # d_reference = 3.5 (anthropometric mean)”
```

Intelligent recognition and evaluation of other training actions follow a similar approach. The specific fitting functions are shown in Table 1.

2.1.3 Data set

Set of feature point capture data: The data set that was chosen comprises a wealth of facial expressions and actions, including 3D facial data and face images or videos captured under varying lighting conditions and angles. This information is utilised to assess and enhance the model's capability in handling non-rigid changes in facial action. Acted Facial

the model's capability in handling non-rigid changes in facial action. Acted Facial Expressions in the Wild (AFEW) are derived from films and authentic videos. Its extensive collection of natural-environment facial expressions makes it suitable for evaluating the model's performance in practical applications[32]. Consequently, this research is grounded in AFEW and employs it to train the model that determines the precision of feature points.
Nasopharyngeal cancer rehabilitation training evaluation dataset: Under the guidance of medical professionals, video data of nasopharyngeal cancer patients performing mouth-opening exercises were collected, followed by segmentation, pretraining, and augmentation processes. These were used to supplement the creation of a dataset for nasopharyngeal cancer mouth-opening training actions, facilitating additional training and testing. This dataset includes image data from N patients (800), where each image contains the patient's facial image and the corresponding manual evaluation result (i.e., the classification of mouth opening status). Due to the noticeable 'severe abnormal' category of difficulty in mouth opening among nasopharyngeal cancer patients, such instances constitute only a small portion of the entire dataset. This imbalance poses significant challenges for predictive modeling. To address the class imbalance issue, strategies were implemented, including oversampling the minority group, undersampling the majority group, or using the Synthetic Minority Over-sampling Technique (SMOTE) to create a more balanced distribution. Figure 5 provides a detailed description of the dataset distribution and action grading indicators, which will be used for further model training.

2.1.4 Validation of precision

To assess the effectiveness of the implemented model, we calculate the positive predictive value or the proportion of accurately identified positive instances among all classified positive instances. This metric assumes particular significance when false alarm expenses are substantial. The recall rate, which is alternatively referred to as sensitivity or true positive rate, measures the percentage of true positive cases that the algorithm accurately detects. In situations where the absence of positive examples (false negatives) can lead to substantial penalties, this is of the utmost importance. As an indicator, the F1 score balances the harmonic mean values of the dependencies 'accuracy' and'recall' through coordination. This indicator is exceptionally valuable as it enables a more comprehensive assessment of the algorithm's performance. Particularly when asymmetrical class distribution is present, the following calculation formulas are applicable:

Accuracy=

(4)

Where TP (True Positive) represents the number of true class samples correctly predicted, TN (True Negative) represents the number of true negative class samples correctly predicted, FP (False Positive) represents the number of false positive class samples incorrectly predicted as positive class, FN (False Negative) represents the number of false negative class samples incorrectly predicted as negative class.

Precision=

(5)

Recall=

(6)

F1=2×

(7)

To evaluate the performance of the proposed model in facial training movement grading and assessment, all collected patient training videos were manually annotated and cross-validated by a panel of 20 clinical oncologists. Following expert comparison, the model achieved an accuracy of 98.2% in the facial training movement grading task. These results underscore the high accuracy and reliability of the developed model, highlighting its potential for practical application in clinical settings.

After calculation, the evaluation was conducted using he AFEW public dataset and the collected dataset of patient mouth-opening training. To demonstrate the performance and

innovation of the proposed method in the field of non-rigid facial landmark detection, the results were compared with the widely used benchmark ResNet series, known for their efficiency and accuracy. The experiment employed four different techniques—encompas failure rates, cumulative error distributions, average error relative to inter-ocular distance, and the precision-recall curve—to compare the performance of ResNet-101, ResNet-152, ResNet-164, Wide ResNet, ResNet-Positional Encoding, and our proposed model.By employing the same hardware and dataset, all models were evaluated, and the performance of the proposed model is shown in Figure 6.

The performance comparison of EffiResNet with other mainstream ResNet variants in computational tasks demonstrates its ability to more effectively balance accuracy, robustness, and computational efficiency. From the perspective of failure rate, EffiResNet exhibits a significantly faster decrease in failure rate as the error threshold increases, compared to other models. This indicates that EffiResNet possesses stronger robustness and adaptability when handling tasks with larger errors. Such characteristics enable EffiResNet to maintain stable performance output in complex and dynamic computational environments.Furthermore, in terms of cumulative error distribution, the curve of EffiResNet is relatively smooth and approaches the CED performance of ResNet-152, indicating that the model maintains relatively stable performance when processing errors of varying sizes. Compared to other ResNet variants, EffiResNet better balances the impact of different error sizes on model performance during error accumulation, thereby ensuring the overall stability of performance.In terms of spatial awareness, the relationship between average error and interocular distance (IoD) shows that the average error curve of EffiResNet is relatively stable, reflecting the model's significant advantage in spatial perception. This enables EffiResNet to more accurately capture and analyze spatial information when handling computational tasks related to spatial positioning.In classification tasks, EffiResNet also demonstrates outstanding performance. The comparison of precision-recall curves reveals that, in contrast to ResNet-Positional Encoding, EffiResNet maintains a high recall rate while also achieving a relatively high precision. This means that EffiResNet is able to more accurately identify target categories in classification tasks while minimizing the likelihood of misclassification.

2.2 Personalized real-time feedback

In designing the virtual avatar for nasopharyngeal cancer patients, we took into account the deficiencies in the sensory integration system while avoiding the uncanny valley effect[33]. A series of brightly colored, simple-structured, soft, and rounded dinosaur virtual headgear avatars were created[34]. These avatars are linked to mouth-opening training movements through gamification by reshaping skeletal point capture, providing visual and auditory feedback, such as swallowing coins, when the required movement is achieved(figure 7). As training scores accumulate, users can unlock additional virtual training avatars as rewards.This design aims to stimulate patients' subjective initiative during training interventions, creating an immediate supportive and personalized training environment.The remote mouth-opening data tracking functionality is developed using the Unity3D engine and Python, with communication implemented through SocketTools.cs. Upon authorization, we utilize the user's mobile camera for facial landmark detection, allowing the system to collect mouth-opening data over a specified period, including, but not limited to, maximum mouth opening, opening frequency, and symmetry. This data is transmitted to the backend via TCP/IP sockets for processing, with serial communication facilitating data exchange between devices, enabling real-time monitoring of patient movements.Moreover, data transmission is secured using encryption protocols to ensure privacy. The collected data is used for long-term daily monitoring of mouth-opening training recovery progress, providing valuable feedback to patients and their healthcare professionals regarding training patterns and behavioral habits."

5-ix) Describe use parameters

Describe use parameters (e.g., intended “doses” and optimal timing for use). Clarify what instructions or recommendations were given to the user, e.g., regarding timing, frequency, heaviness of use, if any, or was the intervention used ad libitum.

	1	2	3	4	5	
subitem not at all important	○	○	○	○	◉	essential

清除所选内容

Does your paper address subitem 5-ix?

Copy and paste relevant sections from the manuscript (include quotes in quotation marks "like this" to indicate direct quotes from your manuscript), or elaborate on this item by providing additional information not in the ms, or briefly explain why the item is not applicable/relevant for your study

您的回答

5-x) Clarify the level of human involvement

Clarify the level of human involvement (care providers or health professionals, also technical assistance) in the e-intervention or as co-intervention (detail number and expertise of professionals involved, if any, as well as “type of assistance offered, the timing and frequency of the support, how it is initiated, and the medium by which the assistance is delivered”. It may be necessary to distinguish between the level of human involvement required for the trial, and the level of human involvement required for a routine application outside of a RCT setting (discuss under item 21 – generalizability).

	1	2	3	4	5	
subitem not at all important	○	○	○	○	◉	essential

清除所选内容

Does your paper address subitem 5-x?

Copy and paste relevant sections from the manuscript (include quotes in quotation marks "like this" to indicate direct quotes from your manuscript), or elaborate on this item by providing additional information not in the ms, or briefly explain why the item is not applicable/relevant for your study

您的回答

5-xi) Report any prompts/reminders used

Report any prompts/reminders used: Clarify if there were prompts (letters, emails, phone calls, SMS) to use the application, what triggered them, frequency etc. It may be necessary to distinguish between the level of prompts/reminders required for the trial, and the level of prompts/reminders for a routine application outside of a RCT setting (discuss under item 21 – generalizability).

	1	2	3	4	5	
subitem not at all important	○	○	○	○	◉	essential

清除所选内容

Does your paper address subitem 5-xi? *

Copy and paste relevant sections from the manuscript (include quotes in quotation marks "like this" to indicate direct quotes from your manuscript), or elaborate on this item by providing additional information not in the ms, or briefly explain why the item is not applicable/relevant for your study

Like this"3.2.2 Involvement
The control group, consisting of participants receiving conventional care, was informed of the benefits of regular mouth-opening exercises and instructed to maintain their usual lifestyle. The duration and intensity of the exercises were carefully adjusted to avoid discomfort. Researchers provided participants with instructional video demonstrations for the mouth-opening exercises at no cost, along with an application designed to track daily maximum mouth opening, symmetry, and the frequency of weekly video-guided practice. The video was meticulously designed and recorded by medical professionals from the oncology radiation department, following strict protocols. It was subsequently validated by a multidisciplinary team of experts, including oncologists specializing in head and neck cancer, oral and maxillofacial surgeons, head and neck tumor specialists, rehabilitation physicians, and physical therapists.
In contrast, the intervention group utilized a health application developed by our team, which included the aforementioned instructional videos and a monitoring feedback feature for tracking exercise performance. Throughout the study, all participants were instructed to refrain from engaging in any additional conventional exercises. Researchers conducted weekly phone follow-ups to assess participants' recovery progress and address any inquiries. Furthermore, another research assistant provided detailed instructions on using the application and answered questions in a dedicated chat group. After each training session, the app recorded users' maximum mouth opening and symmetry, while also collecting data on fatigue levels and user experience for subsequent qualitative analysis."

5-xii) Describe any co-interventions (incl. training/support)

Describe any co-interventions (incl. training/support): Clearly state any interventions that are provided in addition to the targeted eHealth intervention, as ehealth intervention may not be designed as stand-alone intervention. This includes training sessions and support [1]. It may be necessary to distinguish between the level of training required for the trial, and the level of training for a routine application outside of a RCT setting (discuss under item 21 – generalizability.

	1	2	3	4	5	
subitem not at all important	○	○	○	○	◉	essential

清除所选内容

Does your paper address subitem 5-xii? *

Copy and paste relevant sections from the manuscript (include quotes in quotation marks "like this" to indicate direct quotes from your manuscript), or elaborate on this item by providing additional information not in the ms, or briefly explain why the item is not applicable/relevant for your study

Like this"3.2.2 Involvement
The control group, consisting of participants receiving conventional care, was informed of the benefits of regular mouth-opening exercises and instructed to maintain their usual lifestyle. The duration and intensity of the exercises were carefully adjusted to avoid discomfort. Researchers provided participants with instructional video demonstrations for the mouth-opening exercises at no cost, along with an application designed to track daily maximum mouth opening, symmetry, and the frequency of weekly video-guided practice.
The video was meticulously designed and recorded by medical professionals from the oncology radiation department, following strict protocols. It was subsequently validated by a multidisciplinary team of experts, including oncologists specializing in head and neck cancer, oral and maxillofacial surgeons, head and neck tumor specialists, rehabilitation physicians, and physical therapists.
In contrast, the intervention group utilized a health application developed by our team, which included the aforementioned instructional videos and a monitoring feedback feature for tracking exercise performance. Throughout the study, all participants were instructed to refrain from engaging in any additional conventional exercises. Researchers conducted weekly phone follow-ups to assess participants' recovery progress and address any inquiries. Furthermore, another research assistant provided detailed instructions on using the application and answered questions in a dedicated chat group. After each training session, the app recorded users' maximum mouth opening and symmetry, while also collecting data on fatigue levels and user experience for subsequent qualitative analysis."

6a) Completely defined pre-specified primary and secondary outcome measures, including how and when they were assessed

Does your paper address CONSORT subitem 6a? *

Copy and paste relevant sections from the manuscript (include quotes in quotation marks "like this" to indicate direct quotes from your manuscript), or elaborate on this item by providing additional information not in the ms, or briefly explain why the item is not applicable/relevant for your study

Like this"3.3 Outcomes Measures
A validated online or in-person questionnaire was administered to each participant at baseline and four weeks following the intervention. No monetary compensation or other form of incentive was provided to participants in exchange for their participation in the questionnaire or study. The baseline demographic data collection process was comprehensively detailed in the section devoted to results measurement.
3.3.1 Primary Outcomes
Maximum data on mouth aperture.Through the collection and comparison of the subjects' maximum mouth opening data prior to and subsequent to the intervention, one can assess and analyze the impact of the intervention application on the mouth opening function. Subsequently, e oduct's effectiveness in enhancing the mouth opening function can be finalized.
Evaluation of the symmetry of the mouth aperture.By documenting and contrasting the symmetry of the subjects' oral movements prior to and subsequent to the intervention, one can facilitate the assessment of the product's impact on oral function.
Number of exercises per week.The behavior's implementation was quantitatively assessed by comparing and recording the weekly training durations of the participants in the healthy behavior prior to and following the intervention.
HBMQ Inquiry Form. Extensively utilized for the assessment of health behaviors[37].Six aspects of HBM were addressed in the HBMQ devised by Kasmaei et al. (2014),: perceived susceptibility (three items), perceived severity (seven items), perceived benefits (three items), perceived barriers (seven items), self-efficacy (five items), action cues (three items), and behavior (three items)[38]. The responses for all subscale items, with the exception of the behavior subscale, spanned from completely disagree (0) to completely concur (4). In each of the six domains, participants were requested to rate sentences that reflected their beliefs. With optimal psychological measurement characteristics, the behavior subscale requests the frequency of behavior, such as the number of mouth-opening exercises, on a scale from never (0) to always (4).
4.3.2 Secondary Cognitive Outcomes
Borg Rating of Perceived Exertion Scale (BRPE)[39] .Self-rated physical exertion during exercise is quantified using the BRPE, which assigns values between 6 (indicating no exercise intensity) and 20 (representing maximal exercise intensity).
AQoL-6D.The health-related quality of life is quantified using the [40] version of the quality of life assessment tool. A score between -0.04 and 1.00 indicates a superior quality of life.
System Usability Scale (SUS) .Perceived availability is assessed utilizing the system availability scale. Upon successful completion of the designated content assignment, participants have the ability to promptly obtain their scores using SUS[41]. A larger score signifies enhanced system availability of the product."

6a-i) Online questionnaires: describe if they were validated for online use and apply CHERRIES items to describe how the questionnaires were designed/deployed

If outcomes were obtained through online questionnaires, describe if they were validated for online use and apply CHERRIES items to describe how the questionnaires were designed/deployed [9].

	1	2	3	4	5	
subitem not at all important	○	○	○	○	◉	essential

清除所选内容

Does your paper address subitem 6a-i?

Copy and paste relevant sections from manuscript text

您的回答

6a-ii) Describe whether and how “use” (including intensity of use/dosage) was defined/measured/monitored

Describe whether and how “use” (including intensity of use/dosage) was defined/measured/monitored (logins, logfile analysis, etc.). Use/adoption metrics are important process outcomes that should be reported in any ehealth trial.

	1	2	3	4	5	
subitem not at all important	○	○	○	○	◉	essential

清除所选内容

Does your paper address subitem 6a-ii?

Copy and paste relevant sections from manuscript text

您的回答

6a-iii) Describe whether, how, and when qualitative feedback from participants was obtained

Describe whether, how, and when qualitative feedback from participants was obtained (e.g., through emails, feedback forms, interviews, focus groups).

	1	2	3	4	5	
subitem not at all important	○	○	○	○	◉	essential

清除所选内容

Does your paper address subitem 6a-iii?

Copy and paste relevant sections from manuscript text

您的回答

6b) Any changes to trial outcomes after the trial commenced, with reasons

Does your paper address CONSORT subitem 6b? *

Copy and paste relevant sections from the manuscript (include quotes in quotation marks "like this" to indicate direct quotes from your manuscript), or elaborate on this item by providing additional information not in the ms, or briefly explain why the item is not applicable/relevant for your study

Like this"4.4.2 Primary outcomes
Primary outcomes
Table 3 and figure 9 show the results of changes in the HBM subscales and clinical indicators of improved jaw clenching from the baseline to the four-week trial period after the intervention. In terms of perceptual susceptibility, although the changes in the intervention group and the control group did not reach statistical significance (P = 0.074), the intervention group increased from 6.0 at baseline to 7.5, indicating a certain degree of positive change. This was in contrast to the small changes in the control group (from 5.8 to 6.1). In terms of perceived severity, the average value of the intervention group increased from 23.3 to 27.8, the difference was 4.5 (95 % CI : 2.6-6.4), and the P value was 0.007.There was a significant difference, and the control group had little change in this index, only from 24.6 to 25.4. In terms of perceived benefits, the average score of the intervention group increased from 11.2 to 11.9 (the difference was 0.7,95 % CI : 0.2-1.2), which was statistically significant (P = 0.036).At the same time, the reduction of perceptual impairment was particularly significant (from 16.5 to 8.5, the difference was − 8.0, 95 % CI : − 11.0-4.9, P = 0.001).In addition, self-efficacy increased from 5.4 to 6.4 (the difference was 1.0, 95 % CI : 0.6-1.4, P = 0.038), and action cues increased from 7.5 to 11.2 (the difference was 3.7, 95 % CI : 2.2-4.2, P = 0.001), indicating that the intervention successfully improved the confidence and motivation of individuals to adopt healthy behaviors. The increase in the maximum oral opening value (from 36.98 to 39.05, the difference was 2.07,95 % CI : 1.5-2.6, P = 0.044) and the improvement of physical indicators related to oral health showed the potential benefits of intervention in improving specific health behaviors.The intervention group performed significantly more exercises per week (10.0 ± 3.5) compared to the control group (4.2 ± 2.6), with a p-value of 0.001.However, small changes in oral opening symmetry (− 0.014,95 % CI : − 0.053-0.025, P = 0.146) suggested that interventions were not equally effective in all aspects.
Secondary cognitive outcomes
The control group experienced a significant increase in perceived exertion, with a mean difference in BRPE of 1.78 (95% CI, 1.33-2.23, p=0.035*), indicating a notable rise in fatigue levels compared to the intervention group. On the other hand, changes in the quality of life, as measured by AQoL-6D, were not significant in either group.System availability was evaluated using the System Usability Scale (SUS), and the experimental group achieved an average system usability score of 74.28 out of 100. Scores exceeding 70 were classified as good, indicating a high system availability."

7a) How sample size was determined

NPT: When applicable, details of whether and how the clustering by care provides or centers was addressed

7a-i) Describe whether and how expected attrition was taken into account when calculating the sample size

Describe whether and how expected attrition was taken into account when calculating the sample size.

	1	2	3	4	5	
subitem not at all important	○	○	○	○	◉	essential

清除所选内容

Does your paper address subitem 7a-i?

Copy and paste relevant sections from manuscript title (include quotes in quotation marks "like this" to indicate direct quotes from your manuscript), or elaborate on this item by providing additional information not in the ms, or briefly explain why the item is not applicable/relevant for your study

Like this"In our previous comparative study, the sample size was determined by examining the change in perceived benefit assessment one week after the intervention. The study findings indicate that the functional status scores for the control group were 69.85 ± 16.45 and the mean was 72.16 ± 10.80 for the control group.By utilizing the GPower V.3.1.9.2 software, we ascertained that an effect size of 80% and a significance level of 5% (two-tailed) could be achieved with an equivalent number of participants in the sample (108)."

7b) When applicable, explanation of any interim analyses and stopping guidelines

Does your paper address CONSORT subitem 7b? *

Copy and paste relevant sections from the manuscript (include quotes in quotation marks "like this" to indicate direct quotes from your manuscript), or elaborate on this item by providing additional information not in the ms, or briefly explain why the item is not applicable/relevant for your study

Like this"The study participants comprised nasopharyngeal cancer patients who had finished radiotherapy and were prepared to be discharged from the oncology facility. They were recruited via posters and questionnaires.
The following were the criteria for inclusion:(1) Participants were those who had received a pathology or histological diagnosis of nasopharyngeal cancer and were prescribed radiotherapy. (2) Patients were between the ages of 18 and 65; (3) Patients were cognizant of their condition; (4) Patients gave informed consent and agreed to participate in this study; (5) Patients had access to smartphones and WeChat applications; and (6) Patients had Functional Status Scores (KPS) ranging from 80 to 100[35] and Eastern Cancer Cooperative Group (ECOG) scores ranging from 0 to 5[36].
The criteria for exclusion were as follows: (1) Individuals who have a history of radiotherapy; (2) Individuals who are unable to engage in physical activity due to underlying cardiac, neurological, muscular, or joint disease; and (3) Individuals who have undergone pertinent mouth-opening exercises (as part of their daily care, follow-up videos, etc.).
The criteria for withdrawal were as follows: (1) lack of desire to proceed with the trial; (2) onset of a severe illness that hindered trial continuation; (3) adverse event associated with open-mouth exercise or regular physical activity; and (4) Additional surgical interventions, such as mandibular resection."

8a) Method used to generate the random allocation sequence

NPT: When applicable, how care providers were allocated to each trial group

Does your paper address CONSORT subitem 8a? *

Copy and paste relevant sections from the manuscript (include quotes in quotation marks "like this" to indicate direct quotes from your manuscript), or elaborate on this item by providing additional information not in the ms, or briefly explain why the item is not applicable/relevant for your study

Like this"Through the implementation of sealed opaque envelopes that were disseminated by other researchers, we effectively concealed the allocation information from the result evaluators. Participants were strongly encouraged to refrain from disclosing such details during the process of calculating the results."

8b) Type of randomisation; details of any restriction (such as blocking and block size)

Does your paper address CONSORT subitem 8b? *

Copy and paste relevant sections from the manuscript (include quotes in quotation marks "like this" to indicate direct quotes from your manuscript), or elaborate on this item by providing additional information not in the ms, or briefly explain why the item is not applicable/relevant for your study

Like this"The study adopted a double-blind procedure, where both the participants and all data collectors were unaware of the group assignments. A non-blind independent statistician was responsible for data coding for analysis. Prior to recruitment, the statistician generated a random number sequence using STAS and employed block randomization to randomly assign numbers from 1 to 108 to the intervention and control groups, ensuring that the generated sequence was both random and unbiased. To ensure the blinding of the group assignment process, the randomization of researchers was concealed, and sealed opaque envelopes were used to maintain allocation concealment, with participants remaining unaware of the study groups and research hypotheses. Throughout the trial, all participants were instructed not to engage in any additional regular exercise. To avoid influencing exercise adherence behavior, participants were not informed that two independent but related trials were being conducted or that they were being re-randomized into the study. After allocation, participants in the intervention group received an email from the researchers containing information about accessing the digital health application product and arrangements for the on-site trial. Throughout the study, if participants had any questions about the application or the research, they could contact the researchers via phone or email. The statistician remained unaware of the group assignments to ensure the objectivity and accuracy of the data analysis."

9) Mechanism used to implement the random allocation sequence (such as sequentially numbered containers), describing any steps taken to conceal the sequence until interventions were assigned

Does your paper address CONSORT subitem 9? *

Copy and paste relevant sections from the manuscript (include quotes in quotation marks "like this" to indicate direct quotes from your manuscript), or elaborate on this item by providing additional information not in the ms, or briefly explain why the item is not applicable/relevant for your study

Like this"The study adopted a double-blind procedure, where both the participants and all data collectors were unaware of the group assignments. A non-blind independent statistician was responsible for data coding for analysis. Prior to recruitment, the statistician generated a random number sequence using STAS and employed block randomization to randomly assign numbers from 1 to 108 to the intervention and control groups, ensuring that the generated sequence was both random and unbiased. To ensure the blinding of the group assignment process, the randomization of researchers was concealed, and sealed opaque envelopes were used to maintain allocation concealment, with participants remaining unaware of the study groups and research hypotheses. Throughout the trial, all participants were instructed not to engage in any additional regular exercise. To avoid influencing exercise adherence behavior, participants were not informed that two independent but related trials were being conducted or that they were being re-randomized into the study. After allocation, participants in the intervention group received an email from the researchers containing information about accessing the digital health application product and arrangements for the on-site trial. Throughout the study, if participants had any questions about the application or the research, they could contact the researchers via phone or email. The statistician remained unaware of the group assignments to ensure the objectivity and accuracy of the data analysis."

10) Who generated the random allocation sequence, who enrolled participants, and who assigned participants to interventions

Does your paper address CONSORT subitem 10? *

Copy and paste relevant sections from the manuscript (include quotes in quotation marks "like this" to indicate direct quotes from your manuscript), or elaborate on this item by providing additional information not in the ms, or briefly explain why the item is not applicable/relevant for your study

Like this"The study adopted a double-blind procedure, where both the participants and all data collectors were unaware of the group assignments. A non-blind independent statistician was responsible for data coding for analysis. Prior to recruitment, the statistician generated a random number sequence using STAS and employed block randomization to randomly assign numbers from 1 to 108 to the intervention and control groups, ensuring that the generated sequence was both random and unbiased. To ensure the blinding of the group assignment process, the randomization of researchers was concealed, and sealed opaque envelopes were used to maintain allocation concealment, with participants remaining unaware of the study groups and research hypotheses. Throughout the trial, all participants were instructed not to engage in any additional regular exercise. To avoid influencing exercise adherence behavior, participants were not informed that two independent but related trials were being conducted or that they were being re-randomized into the study. After allocation, participants in the intervention group received an email from the researchers containing information about accessing the digital health application product and arrangements for the on-site trial. Throughout the study, if participants had any questions about the application or the research, they could contact the researchers via phone or email. The statistician remained unaware of the group assignments to ensure the objectivity and accuracy of the data analysis."

11a) If done, who was blinded after assignment to interventions (for example, participants, care providers, those assessing outcomes) and how

NPT: Whether or not administering co-interventions were blinded to group assignment

11a-i) Specify who was blinded, and who wasn't

Specify who was blinded, and who wasn't. Usually, in web-based trials it is not possible to blind the participants [1, 3] (this should be clearly acknowledged), but it may be possible to blind outcome assessors, those doing data analysis or those administering co-interventions (if any).

	1	2	3	4	5	
subitem not at all important	○	○	○	○	◉	essential

清除所选内容

Does your paper address subitem 11a-i? *

Copy and paste relevant sections from the manuscript (include quotes in quotation marks "like this" to indicate direct quotes from your manuscript), or elaborate on this item by providing additional information not in the ms, or briefly explain why the item is not applicable/relevant for your study

Like this"The study adopted a double-blind procedure, where both the participants and all data collectors were unaware of the group assignments. A non-blind independent statistician was responsible for data coding for analysis. Prior to recruitment, the statistician generated a random number sequence using STAS and employed block randomization to randomly assign numbers from 1 to 108 to the intervention and control groups, ensuring that the generated sequence was both random and unbiased. To ensure the blinding of the group assignment process, the randomization of researchers was concealed, and sealed opaque envelopes were used to maintain allocation concealment, with participants remaining unaware of the study groups and research hypotheses. Throughout the trial, all participants were instructed not to engage in any additional regular exercise. To avoid influencing exercise adherence behavior, participants were not informed that two independent but related trials were being conducted or that they were being re-randomized into the study. After allocation, participants in the intervention group received an email from the researchers containing information about accessing the digital health application product and arrangements for the on-site trial. Throughout the study, if participants had any questions about the application or the research, they could contact the researchers via phone or email. The statistician remained unaware of the group assignments to ensure the objectivity and accuracy of the data analysis."

11a-ii) Discuss e.g., whether participants knew which intervention was the “intervention of interest” and which one was the “comparator”

Informed consent procedures (4a-ii) can create biases and certain expectations - discuss e.g., whether participants knew which intervention was the “intervention of interest” and which one was the “comparator”.

	1	2	3	4	5	
subitem not at all important	○	○	○	○	◉	essential

清除所选内容

Does your paper address subitem 11a-ii?

Copy and paste relevant sections from the manuscript (include quotes in quotation marks "like this" to indicate direct quotes from your manuscript), or elaborate on this item by providing additional information not in the ms, or briefly explain why the item is not applicable/relevant for your study

您的回答

11b) If relevant, description of the similarity of interventions

(this item is usually not relevant for ehealth trials as it refers to similarity of a placebo or sham intervention to a active medication/intervention)

Does your paper address CONSORT subitem 11b? *

Copy and paste relevant sections from the manuscript (include quotes in quotation marks "like this" to indicate direct quotes from your manuscript), or elaborate on this item by providing additional information not in the ms, or briefly explain why the item is not applicable/relevant for your study

Like this"Digital health interventions can provide scalable health education and self-management support for NPC patients through applications or web-based platforms[12], offering high coverage, low cost, and easy accessibility to mitigate constraints related to time and resources [13-16]. However, the effectiveness of some digital interventions exhibits variability. For instance, while physiotherapy programs and online applications have demonstrated efficacy in improving physical function [17] ,their long-term effectiveness declines due to a lack of precise[18], individualized support. Additionally, a physical activity counseling program based on wearable devices reported no prognostic benefits, as it lacked accurate monitoring and feedback on training movements[19, 20].This limitation diminishes the effectiveness of telemedicine applications in facilitating patient rehabilitation behaviors. A meta-analysis further highlighted that precise movement monitoring and targeted feedback are critical factors in enhancing the effectiveness of digital health interventions for patient health management. Therefore, future digital health interventions should prioritize advancements in personalized, intelligent, and real-time monitoring and feedback technologies to enhance intervention efficacy[21, 22].

In clinical practice, the evaluation of jaw training for patients with limited mouth opening mainly relies on direct observation and manual measurements[23], which have limitations in data objectivity and real-time applicability. Recently, machine learning has shown promise in intelligent assessment and quantitative rehabilitation, providing objective monitoring for clinical care[23].However, NPC patients undergoing radiotherapy may experience skin fibrosis, inflammation, swelling, and mandibular deformities, complicating facial motion analysis. Additionally, factors such as head movement and lighting variations exacerbate non-rigid facial deformations, making accurate motion tracking difficult[24]. Existing methods relying on contact-based markers are prone to facial trauma, patient discomfort, and insufficient real-time tracking accuracy.Thus, developing a deep learning-based, non-contact, real-time monitoring system is crucial for enhancing NPC rehabilitation[25].Recent advancements in artificial intelligence have enabled machines to automatically analyze and interpret complex data, supporting more personalized treatment strategies. For instance,Ding et al. explored dense facial tracking sequences using CNN networks combined with non-rigid ICP algorithms to address frame-to-frame relationships in 3D facial analysis[26]. Honey et al. employed a hybrid deep learning algorithm, including MtCNN and DeepFace, to overcome non-rigid facial changes, such as variations in size, shape, and color [27].Bandaru et al. proposed the Tiefes FCNN model, achieving high accuracy in microexpression recognition for non-rigid facial movements [28, 29], demonstrating the feasibility of CNN-based deep networks.

To address the challenges in rehabilitation training for nasopharyngeal carcinoma (NPC) patients, we present Open Care, a remote healthcare application designed specifically for NPC jaw rehabilitation(Figure 1). By utilizing an EffiFCNN-ResNet model, Open Care enables non-contact, real-time facial motion tracking, measurement, and automated feedback. This system offers a novel solution for personalized, efficient NPC rehabilitation. A randomized controlled trial will evaluate its effectiveness in improving patient adherence and rehabilitation outcomes. This work is innovative as no previous study has integrated deep learning-based non-rigid facial recognition with personalized monitoring and feedback for NPC mouth-opening rehabilitation."

12a) Statistical methods used to compare groups for primary and secondary outcomes

NPT: When applicable, details of whether and how the clustering by care providers or centers was addressed

Does your paper address CONSORT subitem 12a? *

Copy and paste relevant sections from the manuscript (include quotes in quotation marks "like this" to indicate direct quotes from your manuscript), or elaborate on this item by providing additional information not in the ms, or briefly explain why the item is not applicable/relevant for your study

Like this"SPSS version 26.0 was utilized to analyze the data. For quantitative measurements involving categorical variables, descriptive data are utilized in the form of means (M) (accompanied by standard deviations SD) or numbers (N) (with percentages). In order to compare the baseline characteristics of participants who contributed the main results with those who did not, a T-test or chi-square test was employed. In light of the variable distribution, the Mann-Whitney U test was chosen to assess the significance of differences in the data between and within groups prior to and following the intervention test. A comparison was made between the control group and the intervention group in terms of their differences. For all statistical analyses, inter-group and intra-group differences were expressed as the mean and 95% confidence interval (CI). At 0.05, the significance level (alpha) was established.A significance level of 0.05 or lower was employed to classify a difference as statistically significant."

12a-i) Imputation techniques to deal with attrition / missing values

Imputation techniques to deal with attrition / missing values: Not all participants will use the intervention/comparator as intended and attrition is typically high in ehealth trials. Specify how participants who did not use the application or dropped out from the trial were treated in the statistical analysis (a complete case analysis is strongly discouraged, and simple imputation techniques such as LOCF may also be problematic [4]).

	1	2	3	4	5	
subitem not at all important	○	○	○	○	●	essential

清除所选内容

Does your paper address subitem 12a-i? *

Copy and paste relevant sections from the manuscript (include quotes in quotation marks "like this" to indicate direct quotes from your manuscript), or elaborate on this item by providing additional information not in the ms, or briefly explain why the item is not applicable/relevant for your study

Like this"A total of 210 patients were screened for eligibility; 68 patients did not meet the eligibility criteria and were excluded. 133 patients agreed to participate and were randomly assigned to either the app intervention group (n = 66) or the routine care control group (n = 67). All participants provided signed informed consent. During the study, 12 participants in the app intervention group were excluded due to the following reasons: removal of the upper central incisor during surgery (n = 9), use of facial orthodontic appliances due to dental conditions (n = 1), and failure to return to the care department for follow-up (n = 2). In the routine care control group, 13 participants were excluded for the following reasons: removal of the upper central incisor during follow-up treatment (n = 8), postoperative wound bleeding after discharge (n = 3), and failure to return for follow-up (n = 2). This resulted in 108 valid cases, with 54 cases in each group. For each group, the analysis included 54 participants, with analysis conducted based on the original assigned groups, as outlined in the CONSORT (Consolidated Standards of Reporting Trials)[42] flowchart (Figure 8).
Data analysis and baseline measurements were performed on a sample of 108 participants who satisfied the recruitment criteria. Males(68.75 %) comprised the majority of the sample (47.31 ± 8.47 years old), which was consistent with the prevalence of nasopharyngeal carcinoma . Table 2 presents the outcomes pertaining to various factors including nasopharyngeal carcinoma-related indicators, maximal mouth opening, mouth opening symmetry, weekly training frequency, gender, age, education level, and the Shapiro-Wilk test.There was no significant difference in the information of each group ($p > 0.05$), which could be used for subsequent experiments and analysis. At baseline, According to the evaluation of the subjects ' cancer-r"

12b) Methods for additional analyses, such as subgroup analyses and adjusted analyses

Does your paper address CONSORT subitem 12b? *

Copy and paste relevant sections from the manuscript (include quotes in quotation marks "like this" to indicate direct quotes from your manuscript), or elaborate on this item by providing additional information not in the ms, or briefly explain why the item is not applicable/relevant for your study

Like this"SPSS version 26.0 was utilized to analyze the data. For quantitative measurements involving categorical variables, descriptive data are utilized in the form of means (M) (accompanied by standard deviations SD) or numbers (N) (with percentages). In order to compare the baseline characteristics of participants who contributed the main results with those who did not, a T-test or chi-square test was employed. In light of the variable distribution, the Mann-Whitney U test was chosen to assess the significance of differences in the data between and within groups prior to and following the intervention test. A comparison was made between the control group and the intervention group in terms of their differences. For all statistical analyses, inter-group and intra-group differences were expressed as the mean and 95% confidence interval (CI). At 0.05, the significance level (alpha) was established.A significance level of 0.05 or lower was employed to classify a difference as statistically significant."

X26) REB/IRB Approval and Ethical Considerations [recommended as subheading under "Methods"] (not a CONSORT item)

X26-i) Comment on ethics committee approval

	1	2	3	4	5	
subitem not at all important	○	○	○	○	◉	essential

清除所选内容

Does your paper address subitem X26-i?

Copy and paste relevant sections from the manuscript (include quotes in quotation marks "like this" to indicate direct quotes from your manuscript), or elaborate on this item by providing additional information not in the ms, or briefly explain why the item is not applicable/relevant for your study

您的回答

x26-ii) Outline informed consent procedures

Outline informed consent procedures e.g., if consent was obtained offline or online (how? Checkbox, etc.?), and what information was provided (see 4a-ii). See [6] for some items to be included in informed consent documents.

	1	2	3	4	5	
subitem not at all important	○	○	○	○	◉	essential

清除所选内容

Does your paper address subitem X26-ii?

Copy and paste relevant sections from the manuscript (include quotes in quotation marks "like this" to indicate direct quotes from your manuscript), or elaborate on this item by providing additional information not in the ms, or briefly explain why the item is not applicable/relevant for your study

您的回答

X26-iii) Safety and security procedures

Safety and security procedures, incl. privacy considerations, and any steps taken to reduce the likelihood or detection of harm (e.g., education and training, availability of a hotline)

	1	2	3	4	5	
subitem not at all important	○	○	○	○	◉	essential

清除所选内容

Does your paper address subitem X26-iii?

Copy and paste relevant sections from the manuscript (include quotes in quotation marks "like this" to indicate direct quotes from your manuscript), or elaborate on this item by providing additional information not in the ms, or briefly explain why the item is not applicable/relevant for your study

您的回答

RESULTS

13a) For each group, the numbers of participants who were randomly assigned, received intended treatment, and were analysed for the primary outcome
NPT: The number of care providers or centers performing the intervention in each group and the number of patients treated by each care provider in each center

Does your paper address CONSORT subitem 13a? *

Copy and paste relevant sections from the manuscript (include quotes in quotation marks "like this" to indicate direct quotes from your manuscript), or elaborate on this item by providing additional information not in the ms, or briefly explain why the item is not applicable/relevant for your study

Like this"4.4.1 Characteristics at Baseline
A total of 210 patients were screened for eligibility; 68 patients did not meet the eligibility criteria and were excluded. 133 patients agreed to participate and were randomly assigned to either the app intervention group (n = 66) or the routine care control group (n = 67). All participants provided signed informed consent. During the study, 12 participants in the app intervention group were excluded due to the following reasons: removal of the upper central incisor during surgery (n = 9), use of facial orthodontic appliances due to dental conditions (n = 1), and failure to return to the care department for follow-up (n = 2). In the routine care control group, 13 participants were excluded for the following reasons: removal of the upper central incisor during follow-up treatment (n = 8), postoperative wound bleeding after discharge (n = 3), and failure to return for follow-up (n = 2). This resulted in 108 valid cases, with 54 cases in each group. For each group, the analysis included 54 participants, with analysis conducted based on the original assigned groups, as outlined in the CONSORT (Consolidated Standards of Reporting Trials)[42] flowchart (Figure 8).
Data analysis and baseline measurements were performed on a sample of 108 participants who satisfied the recruitment criteria. Males(68.75 %) comprised the majority of the sample (47.31 ± 8.47 years old), which was consistent with the prevalence of nasopharyngeal carcinoma . Table 2 presents the outcomes pertaining to various factors including nasopharyngeal carcinoma-related indicators, maximal mouth opening, mouth opening symmetry, weekly training frequency, gender, age, education level, and the Shapiro-Wilk test.There was no significant difference in the information of each group ($p > 0.05$), which could be used for subsequent experiments and analysis. At baseline, According to the evaluation of the subjects ' cancer-related function and the ability to affect the quality of life after radiotherapy, the mouth opening training can be supported."

13b) For each group, losses and exclusions after randomisation, together with reasons

Does your paper address CONSORT subitem 13b? (NOTE: Preferably, this is shown in a CONSORT flow diagram) *

Copy and paste relevant sections from the manuscript (include quotes in quotation marks "like this" to indicate direct quotes from your manuscript), or elaborate on this item by providing additional information not in the ms, or briefly explain why the item is not applicable/relevant for your study

Like this"4.4.1 Characteristics at Baseline
A total of 210 patients were screened for eligibility; 68 patients did not meet the eligibility criteria and were excluded. 133 patients agreed to participate and were randomly assigned to either the app intervention group (n = 66) or the routine care control group (n = 67). All participants provided signed informed consent. During the study, 12 participants in the app intervention group were excluded due to the following reasons: removal of the upper central incisor during surgery (n = 9), use of facial orthodontic appliances due to dental conditions (n = 1), and failure to return to the care department for follow-up (n = 2). In the routine care control group, 13 participants were excluded for the following reasons: removal of the upper central incisor during follow-up treatment (n = 8), postoperative wound bleeding after discharge (n = 3), and failure to return for follow-up (n = 2). This resulted in 108 valid cases, with 54 cases in each group. For each group, the analysis included 54 participants, with analysis conducted based on the original assigned groups, as outlined in the CONSORT (Consolidated Standards of Reporting Trials)[42] flowchart (Figure 8).
Data analysis and baseline measurements were performed on a sample of 108 participants who satisfied the recruitment criteria. Males(68.75 %) comprised the majority of the sample (47.31 ± 8.47 years old), which was consistent with the prevalence of nasopharyngeal carcinoma . Table 2 presents the outcomes pertaining to various factors including nasopharyngeal carcinoma-related indicators, maximal mouth opening, mouth opening symmetry, weekly training frequency, gender, age, education level, and the Shapiro-Wilk test.There was no significant difference in the information of each group (p > 0.05), which could be used for subsequent experiments and analysis. At baseline, According to the evaluation of the subjects ' cancer-related function and the ability to affect the quality of life after radiotherapy, the mouth opening training can be supported."

13b-i) Attrition diagram

Strongly recommended: An attrition diagram (e.g., proportion of participants still logging in or using the intervention/comparator in each group plotted over time, similar to a survival curve) or other figures or tables demonstrating usage/dose/engagement.

	1	2	3	4	5	
subitem not at all important	○	○	○	○	◉	essential

清除所选内容

Does your paper address subitem 13b-i?

Copy and paste relevant sections from the manuscript or cite the figure number if applicable (include quotes in quotation marks "like this" to indicate direct quotes from your manuscript), or elaborate on this item by providing additional information not in the ms, or briefly explain why the item is not applicable/relevant for your study

您的回答

14a) Dates defining the periods of recruitment and follow-up

Does your paper address CONSORT subitem 14a? *

Copy and paste relevant sections from the manuscript (include quotes in quotation marks "like this" to indicate direct quotes from your manuscript), or elaborate on this item by providing additional information not in the ms, or briefly explain why the item is not applicable/relevant for your study

Like this"During routine checks in the oncology nursing department of a public tertiary hospital, the principal investigator interacted with patients. They were provided with a summary of the study details and, after receiving written informed consent, eligible patients who were interested were screened. Subsequently, patients were randomly assigned to either the intervention group or the control group by selecting a number from an opaque envelope. The participants then completed an online demographic form and baseline questionnaire. At two time points (immediately after 1 month of intervention and immediately after 3 months of intervention), patients were sent information via email requesting them to come to the hospital for data collection and measurement. All data were collected using an online self-report questionnaire through a secure and ethically approved platform, with the questionnaire link shared by the principal investigator via email at the corresponding time points.

The risk of cross-contamination between the intervention and control groups was minimized as the principal investigator recruited each patient separately in the nursing department, ensuring that patients were unaware of other study participants. A separate password-protected account was created for each participant in the intervention group to access the Opencare digital health application, preventing control group participants from accessing the intervention. Additionally, during the recruitment phase, the principal investigator informed all participants not to discuss their participation in the study with others to minimize inter-group cross-contamination.”

14a-i) Indicate if critical “secular events” fell into the study period

Indicate if critical “secular events” fell into the study period, e.g., significant changes in Internet resources available or “changes in computer hardware or Internet delivery resources”

	1	2	3	4	5	
subitem not at all important	○	○	○	○	◉	essential

清除所选内容

Does your paper address subitem 14a-i?

Copy and paste relevant sections from the manuscript (include quotes in quotation marks "like this" to indicate direct quotes from your manuscript), or elaborate on this item by providing additional information not in the ms, or briefly explain why the item is not applicable/relevant for your study

您的回答

14b) Why the trial ended or was stopped (early)

Does your paper address CONSORT subitem 14b? *

Copy and paste relevant sections from the manuscript (include quotes in quotation marks "like this" to indicate direct quotes from your manuscript), or elaborate on this item by providing additional information not in the ms, or briefly explain why the item is not applicable/relevant for your study

Like this"During routine checks in the oncology nursing department of a public tertiary hospital, the principal investigator interacted with patients. They were provided with a summary of the study details and, after receiving written informed consent, eligible patients who were interested were screened. Subsequently, patients were randomly assigned to either the intervention group or the control group by selecting a number from an opaque envelope. The participants then completed an online demographic form and baseline questionnaire. At two time points (immediately after 1 month of intervention and immediately after 3 months of intervention), patients were sent information via email requesting them to come to the hospital for data collection and measurement. All data were collected using an online self-report questionnaire through a secure and ethically approved platform, with the questionnaire link shared by the principal investigator via email at the corresponding time points.

The risk of cross-contamination between the intervention and control groups was minimized as the principal investigator recruited each patient separately in the nursing department, ensuring that patients were unaware of other study participants. A separate password-protected account was created for each participant in the intervention group to access the Opencare digital health application, preventing control group participants from accessing the intervention. Additionally, during the recruitment phase, the principal investigator informed all participants not to discuss their participation in the study with others to minimize inter-group cross-contamination."

15) A table showing baseline demographic and clinical characteristics for each group

NPT: When applicable, a description of care providers (case volume, qualification, expertise, etc.) and centers (volume) in each group

Does your paper address CONSORT subitem 15? *

Copy and paste relevant sections from the manuscript (include quotes in quotation marks "like this" to indicate direct quotes from your manuscript), or elaborate on this item by providing additional information not in the ms, or briefly explain why the item is not applicable/relevant for your study

Like this"4.4.1 Characteristics at Baseline
A total of 210 patients were screened for eligibility; 68 patients did not meet the eligibility criteria and were excluded. 133 patients agreed to participate and were randomly assigned to either the app intervention group (n = 66) or the routine care control group (n = 67). All participants provided signed informed consent. During the study, 12 participants in the app intervention group were excluded due to the following reasons: removal of the upper central incisor during surgery (n = 9), use of facial orthodontic appliances due to dental conditions (n = 1), and failure to return to the care department for follow-up (n = 2). In the routine care control group, 13 participants were excluded for the following reasons: removal of the upper central incisor during follow-up treatment (n = 8), postoperative wound bleeding after discharge (n = 3), and failure to return for follow-up (n = 2). This resulted in 108 valid cases, with 54 cases in each group. For each group, the analysis included 54 participants, with analysis conducted based on the original assigned groups, as outlined in the CONSORT (Consolidated Standards of Reporting Trials)[42] flowchart (Figure 8).
Data analysis and baseline measurements were performed on a sample of 108 participants who satisfied the recruitment criteria. Males(68.75 %) comprised the majority of the sample (47.31 ± 8.47 years old), which was consistent with the prevalence of nasopharyngeal carcinoma . Table 2 presents the outcomes pertaining to various factors including nasopharyngeal carcinoma-related indicators, maximal mouth opening, mouth opening symmetry, weekly training frequency, gender, age, education level, and the Shapiro-Wilk test.There was no significant difference in the information of each group ($p > 0.05$), which could be used for subsequent experiments and analysis. At baseline, According to the evaluation of the subjects ' cancer-related function and the ability to affect the quality of life after radiotherapy, the mouth opening training can be supported."

15-i) Report demographics associated with digital divide issues

In ehealth trials it is particularly important to report demographics associated with digital divide issues, such as age, education, gender, social-economic status, computer/Internet/ehealth literacy of the participants, if known.

	1	2	3	4	5	
subitem not at all important	○	○	○	○	◉	essential

清除所选内容

Does your paper address subitem 15-i? *

Copy and paste relevant sections from the manuscript (include quotes in quotation marks "like this" to indicate direct quotes from your manuscript), or elaborate on this item by providing additional information not in the ms, or briefly explain why the item is not applicable/relevant for your study

Like this"4.4.1 Characteristics at Baseline
A total of 210 patients were screened for eligibility; 68 patients did not meet the eligibility criteria and were excluded. 133 patients agreed to participate and were randomly assigned to either the app intervention group (n = 66) or the routine care control group (n = 67). All participants provided signed informed consent. During the study, 12 participants in the app intervention group were excluded due to the following reasons: removal of the upper central incisor during surgery (n = 9), use of facial orthodontic appliances due to dental conditions (n = 1), and failure to return to the care department for follow-up (n = 2). In the routine care control group, 13 participants were excluded for the following reasons: removal of the upper central incisor during follow-up treatment (n = 8), postoperative wound bleeding after discharge (n = 3), and failure to return for follow-up (n = 2). This resulted in 108 valid cases, with 54 cases in each group. For each group, the analysis included 54 participants, with analysis conducted based on the original assigned groups, as outlined in the CONSORT (Consolidated Standards of Reporting Trials)[42] flowchart (Figure 8).
Data analysis and baseline measurements were performed on a sample of 108 participants who satisfied the recruitment criteria. Males(68.75 %) comprised the majority of the sample (47.31 ± 8.47 years old), which was consistent with the prevalence of nasopharyngeal carcinoma . Table 2 presents the outcomes pertaining to various factors including nasopharyngeal carcinoma-related indicators, maximal mouth opening, mouth opening symmetry, weekly training frequency, gender, age, education level, and the Shapiro-Wilk test.There was no significant difference in the information of each group (p > 0.05), which could be used for subsequent experiments and analysis. At baseline, According to the evaluation of the subjects ' cancer-related function and the ability to affect the quality of life after radiotherapy, the mouth opening training can be supported."

16) For each group, number of participants (denominator) included in each analysis and whether the analysis was by original assigned groups

16-i) Report multiple “denominators” and provide definitions

Report multiple “denominators” and provide definitions: Report N’s (and effect sizes) “across a range of study participation [and use] thresholds” [1], e.g., N exposed, N consented, N used more than x times, N used more than y weeks, N participants “used” the intervention/comparator at specific pre-defined time points of interest (in absolute and relative numbers per group). Always clearly define “use” of the intervention.

	1	2	3	4	5	
subitem not at all important	○	○	○	○	◉	essential

清除所选内容

Does your paper address subitem 16-i? *

Copy and paste relevant sections from the manuscript (include quotes in quotation marks "like this" to indicate direct quotes from your manuscript), or elaborate on this item by providing additional information not in the ms, or briefly explain why the item is not applicable/relevant for your study

Like this"3.1.3 Size of Sample
In our previous comparative study, the sample size was determined by examining the change in perceived benefit assessment one week after the intervention. The study findings indicate that the functional status scores for the control group were 69.85 ± 16.45 and the mean was 72.16 ± 10.80 for the control group.By utilizing the GPower V.3.1.9.2 software, we ascertained that an effect size of 80% and a significance level of 5% (two-tailed) could be achieved with an equivalent number of participants in the sample (108)."

16-ii) Primary analysis should be intent-to-treat

Primary analysis should be intent-to-treat, secondary analyses could include comparing only “users”, with the appropriate caveats that this is no longer a randomized sample (see 18-i).

	1	2	3	4	5	
subitem not at all important	○	○	○	○	◉	essential

清除所选内容

Does your paper address subitem 16-ii?

Copy and paste relevant sections from the manuscript (include quotes in quotation marks "like this" to indicate direct quotes from your manuscript), or elaborate on this item by providing additional information not in the ms, or briefly explain why the item is not applicable/relevant for your study

您的回答

17a) For each primary and secondary outcome, results for each group, and the estimated effect size and its precision (such as 95% confidence interval)

Does your paper address CONSORT subitem 17a? *

Copy and paste relevant sections from the manuscript (include quotes in quotation marks "like this" to indicate direct quotes from your manuscript), or elaborate on this item by providing additional information not in the ms, or briefly explain why the item is not applicable/relevant for your study

Like this"4.4.2 Primary outcomes
Primary outcomes
Table 3 and figure 9 show the results of changes in the HBM subscales and clinical indicators of improved jaw clenching from the baseline to the four-week trial period after the intervention. In terms of perceptual susceptibility, although the changes in the intervention group and the control group did not reach statistical significance (P = 0.074), the intervention group increased from 6.0 at baseline to 7.5, indicating a certain degree of positive change. This was in contrast to the small changes in the control group (from 5.8 to 6.1). In terms of perceived severity, the average value of the intervention group increased from 23.3 to 27.8, the difference was 4.5 (95 % CI : 2.6-6.4), and the P value was 0.007.There was a significant difference, and the control group had little change in this index, only from 24.6 to 25.4. In terms of perceived benefits, the average score of the intervention group increased from 11.2 to 11.9 (the difference was 0.7,95 % CI : 0.2-1.2), which was statistically significant (P = 0.036).At the same time, the reduction of perceptual impairment was particularly significant (from 16.5 to 8.5, the difference was − 8.0, 95 % CI : − 11.0-4.9, P = 0.001).In addition, self-efficacy increased from 5.4 to 6.4 (the difference was 1.0, 95 % CI : 0.6-1.4, P = 0.038), and action cues increased from 7.5 to 11.2 (the difference was 3.7, 95 % CI : 2.2-4.2, P = 0.001), indicating that the intervention successfully improved the confidence and motivation of individuals to adopt healthy behaviors. The increase in the maximum oral opening value (from 36.98 to 39.05, the difference was 2.07,95 % CI : 1.5-2.6, P = 0.044) and the improvement of physical indicators related to oral health showed the potential benefits of intervention in improving specific health behaviors.The intervention group performed significantly more exercises per week (10.0 ± 3.5) compared to the control group (4.2 ± 2.6), with a p-value of 0.001.However, small changes in oral opening symmetry (− 0.014,95 % CI : − 0.053-0.025, P = 0.146) suggested that interventions were not equally effective in all aspects.
Secondary cognitive outcomes
The control group experienced a significant increase in perceived exertion, with a mean difference in BRPE of 1.78 (95% CI, 1.33-2.23, p=0.035*), indicating a notable rise in fatigue levels compared to the intervention group. On the other hand, changes in the quality of life, as measured by AQoL-6D, were not significant in either group.System availability was evaluated using the System Usability Scale (SUS), and the experimental group achieved an average system usability score of 74.28 out of 100. Scores exceeding 70 were classified as good, indicating a high system availability."

17a-i) Presentation of process outcomes such as metrics of use and intensity of use

In addition to primary/secondary (clinical) outcomes, the presentation of process outcomes such as metrics of use and intensity of use (dose, exposure) and their operational definitions is critical. This does not only refer to metrics of attrition (13-b) (often a binary variable), but also to more continuous exposure metrics such as “average session length”. These must be accompanied by a technical description how a metric like a “session” is defined (e.g., timeout after idle time) [1] (report under item 6a).

	1	2	3	4	5	
subitem not at all important	○	○	○	○	◉	essential

清除所选内容

Does your paper address subitem 17a-i?

Copy and paste relevant sections from the manuscript (include quotes in quotation marks "like this" to indicate direct quotes from your manuscript), or elaborate on this item by providing additional information not in the ms, or briefly explain why the item is not applicable/relevant for your study

您的回答

17b) For binary outcomes, presentation of both absolute and relative effect sizes is recommended

Does your paper address CONSORT subitem 17b? *

Copy and paste relevant sections from the manuscript (include quotes in quotation marks "like this" to indicate direct quotes from your manuscript), or elaborate on this item by providing additional information not in the ms, or briefly explain why the item is not applicable/relevant for your study

Like this"4.4.2 Primary outcomes
Primary outcomes
Table 3 and figure 9 show the results of changes in the HBM subscales and clinical indicators of improved jaw clenching from the baseline to the four-week trial period after the intervention. In terms of perceptual susceptibility, although the changes in the intervention group and the control group did not reach statistical significance (P = 0.074), the intervention group increased from 6.0 at baseline to 7.5, indicating a certain degree of positive change. This was in contrast to the small changes in the control group (from 5.8 to 6.1). In terms of perceived severity, the average value of the intervention group increased from 23.3 to 27.8, the difference was 4.5 (95 % CI : 2.6-6.4), and the P value was 0.007.There was a significant difference, and the control group had little change in this index, only from 24.6 to 25.4. In terms of perceived benefits, the average score of the intervention group increased from 11.2 to 11.9 (the difference was 0.7,95 % CI : 0.2-1.2), which was statistically significant (P = 0.036).At the same time, the reduction of perceptual impairment was particularly significant (from 16.5 to 8.5, the difference was − 8.0, 95 % CI : − 11.0-4.9, P = 0.001).In addition, self-efficacy increased from 5.4 to 6.4 (the difference was 1.0, 95 % CI : 0.6-1.4, P = 0.038), and action cues increased from 7.5 to 11.2 (the difference was 3.7, 95 % CI : 2.2-4.2, P = 0.001), indicating that the intervention successfully improved the confidence and motivation of individuals to adopt healthy behaviors. The increase in the maximum oral opening value (from 36.98 to 39.05, the difference was 2.07,95 % CI : 1.5-2.6, P = 0.044) and the improvement of physical indicators related to oral health showed the potential benefits of intervention in improving specific health behaviors.The intervention group performed significantly more exercises per week (10.0 ± 3.5) compared to the control group (4.2 ± 2.6), with a p-value of 0.001.However, small changes in oral opening symmetry (− 0.014,95 % CI : − 0.053-0.025, P = 0.146) suggested that interventions were not equally effective in all aspects.
Secondary cognitive outcomes
The control group experienced a significant increase in perceived exertion, with a mean difference in BRPE of 1.78 (95% CI, 1.33-2.23, p=0.035*), indicating a notable rise in fatigue levels compared to the intervention group. On the other hand, changes in the quality of life, as measured by AQoL-6D, were not significant in either group.System availability was evaluated using the System Usability Scale (SUS), and the experimental group achieved an average system usability score of 74.28 out of 100. Scores exceeding 70 were classified as good, indicating a high system availability."

18) Results of any other analyses performed, including subgroup analyses and adjusted analyses, distinguishing pre-specified from exploratory

Does your paper address CONSORT subitem 18? *

Copy and paste relevant sections from the manuscript (include quotes in quotation marks "like this" to indicate direct quotes from your manuscript), or elaborate on this item by providing additional information not in the ms, or briefly explain why the item is not applicable/relevant for your study

Like this"4.4.2 Primary outcomes
Primary outcomes
Table 3 and figure 9 show the results of changes in the HBM subscales and clinical indicators of improved jaw clenching from the baseline to the four-week trial period after the intervention. In terms of perceptual susceptibility, although the changes in the intervention group and the control group did not reach statistical significance (P = 0.074), the intervention group increased from 6.0 at baseline to 7.5, indicating a certain degree of positive change. This was in contrast to the small changes in the control group (from 5.8 to 6.1). In terms of perceived severity, the average value of the intervention group increased from 23.3 to 27.8, the difference was 4.5 (95 % CI : 2.6-6.4), and the P value was 0.007.There was a significant difference, and the control group had little change in this index, only from 24.6 to 25.4. In terms of perceived benefits, the average score of the intervention group increased from 11.2 to 11.9 (the difference was 0.7,95 % CI : 0.2-1.2), which was statistically significant (P = 0.036).At the same time, the reduction of perceptual impairment was particularly significant (from 16.5 to 8.5, the difference was − 8.0, 95 % CI : − 11.0-4.9, P = 0.001).In addition, self-efficacy increased from 5.4 to 6.4 (the difference was 1.0, 95 % CI : 0.6-1.4, P = 0.038), and action cues increased from 7.5 to 11.2 (the difference was 3.7, 95 % CI : 2.2-4.2, P = 0.001), indicating that the intervention successfully improved the confidence and motivation of individuals to adopt healthy behaviors. The increase in the maximum oral opening value (from 36.98 to 39.05, the difference was 2.07,95 % CI : 1.5-2.6, P = 0.044) and the improvement of physical indicators related to oral health showed the potential benefits of intervention in improving specific health behaviors.The intervention group performed significantly more exercises per week (10.0 ± 3.5) compared to the control group (4.2 ± 2.6), with a p-value of 0.001.However, small changes in oral opening symmetry (− 0.014,95 % CI : − 0.053-0.025, P = 0.146) suggested that interventions were not equally effective in all aspects.
Secondary cognitive outcomes
The control group experienced a significant increase in perceived exertion, with a mean difference in BRPE of 1.78 (95% CI, 1.33-2.23, p=0.035*), indicating a notable rise in fatigue levels compared to the intervention group. On the other hand, changes in the quality of life, as measured by AQoL-6D, were not significant in either group.System availability was evaluated using the System Usability Scale (SUS), and the experimental group achieved an average system usability score of 74.28 out of 100. Scores exceeding 70 were classified as good, indicating a high system availability."

18-i) Subgroup analysis of comparing only users

A subgroup analysis of comparing only users is not uncommon in ehealth trials, but if done, it must be stressed that this is a self-selected sample and no longer an unbiased sample from a randomized trial (see 16-iii).

	1	2	3	4	5	
subitem not at all important	○	○	○	○	◉	essential

清除所选内容

Does your paper address subitem 18-i?

Copy and paste relevant sections from the manuscript (include quotes in quotation marks "like this" to indicate direct quotes from your manuscript), or elaborate on this item by providing additional information not in the ms, or briefly explain why the item is not applicable/relevant for your study

您的回答

19) All important harms or unintended effects in each group

(for specific guidance see CONSORT for harms)

Does your paper address CONSORT subitem 19? *

Copy and paste relevant sections from the manuscript (include quotes in quotation marks "like this" to indicate direct quotes from your manuscript), or elaborate on this item by providing additional information not in the ms, or briefly explain why the item is not applicable/relevant for your study

Like this"In terms of objective clinical indicators, our clinical trial reported significant differences in the maximum mouth opening and frequency of mouth-opening exercises, a finding that is promising and demonstrates better clinical outcomes compared to previous remote support studies[44, 47, 48]. After the intervention, the maximum mouth opening value increased from 36.98 to 39.05, which corresponds to improvements in physical indicators related to oral health. In the intervention group, the average increase in maximum mouth opening was 2.07 mm, with a median increase ranging from 1.5 mm to 2.6 mm. Additionally, within four weeks after discharge, the number of subjects with clenched jaws decreased, and the magnitude of this change was comparable to findings in previous studies involving head and neck cancer patients[49, 50], indicating the potential benefits of the intervention in improving specific health behaviors.Furthermore, treatment adherence is a critical determinant of the success of any preventive exercise program[11, 51]. We observed that the intervention group performed significantly more exercises per week (10.0 ± 3.5) compared to the control group (4.2 ± 2.6), with a p-value of 0.001, demonstrating the significant impact of our developed application on improving patient adherence to home rehabilitation exercises. However, despite the significant improvement in maximum mouth opening, 10% of participants in the experimental group still exhibited clenched jaws during follow-up, suggesting that a longer intervention period may be required,and our study did not find that the application was related to the improvement of mouth opening symmetry, which is contrary to previous studies. Part of the reason may be due to the different duration of the study and the difference in the type and level of functional support provided.[52]A comprehensive strengthening of the facial nerve structure and musculature is required for symmetry to improve; short-term interventions are ineffective.[53]

In terms of improvements in rehabilitation training behaviors, the remote healthcare intervention group in this study, compared to the standard care control group, demonstrated a significant enhancement in patients' perceived benefits and self-efficacy ($p < 0.05$). These findings are consistent with previous studies, such as the research by Jeihooni et al., which examined the impact of remote healthcare-based educational programs on improving oral health training behaviors in pregnant women. The results of this randomized controlled trial showed significant improvements in oral health beliefs and clinical training indicators, similar to the present study, suggesting that remote healthcare interventions can effectively improve individuals' health cognition and behavior change. This supports the rational use of such models to improve health behaviors in a wide range of environments and populations[54, 55].The remote healthcare tool developed in this study provides patients with daily training reminders, real-time supervision of training movements, corrective prompts, and periodic visual training score reports. This semi-supervised, freely accessible intervention appears to be an effective choice for enhancing perceived benefits and self-efficacy[56]. Compared to traditional educational methods, it is more cost-effective and offers greater penetration[57]. The significant improvements in the Perceived Severity and Perceived Benefits indicators ($p < 0.05$) further validate the effectiveness of the intervention. Our application, utilizing the EffiFCNN-ResNet deep learning model and computer vision, tracks participants' mouth-opening movements to facilitate appropriate exercise and training plans while providing personalized feedback for patients at different levels. This may also effectively promote the growth of self-efficacy among patients, thereby contributing to clinical evidence for personalized healthcare design indicators in remote healthcare, as reported in a meta-analysis[58]."

19-i) Include privacy breaches, technical problems

Include privacy breaches, technical problems. This does not only include physical “harm” to participants, but also incidents such as perceived or real privacy breaches [1], technical problems, and other unexpected/unintended incidents. “Unintended effects” also includes unintended positive effects [2].

	1	2	3	4	5	
subitem not at all important	◉	○	○	○	○	essential

清除所选内容

Does your paper address subitem 19-i?

Copy and paste relevant sections from the manuscript (include quotes in quotation marks "like this" to indicate direct quotes from your manuscript), or elaborate on this item by providing additional information not in the ms, or briefly explain why the item is not applicable/relevant for your study

您的回答

19-ii) Include qualitative feedback from participants or observations from staff/researchers

Include qualitative feedback from participants or observations from staff/researchers, if available, on strengths and shortcomings of the application, especially if they point to unintended/unexpected effects or uses. This includes (if available) reasons for why people did or did not use the application as intended by the developers.

	1	2	3	4	5	
subitem not at all important	○	○	○	○	◉	essential

清除所选内容

Does your paper address subitem 19-ii?

Copy and paste relevant sections from the manuscript (include quotes in quotation marks "like this" to indicate direct quotes from your manuscript), or elaborate on this item by providing additional information not in the ms, or briefly explain why the item is not applicable/relevant for your study

您的回答

DISCUSSION

22) Interpretation consistent with results, balancing benefits and harms, and considering other relevant evidence

NPT: In addition, take into account the choice of the comparator, lack of or partial blinding, and unequal expertise of care providers or centers in each group

22-i) Restate study questions and summarize the answers suggested by the data, starting with primary outcomes and process outcomes (use)

Restate study questions and summarize the answers suggested by the data, starting with primary outcomes and process outcomes (use).

	1	2	3	4	5	
subitem not at all important	○	○	○	○	◉	essential

清除所选内容

Does your paper address subitem 22-i? *

Copy and paste relevant sections from the manuscript (include quotes in quotation marks "like this" to indicate direct quotes from your manuscript), or elaborate on this item by providing additional information not in the ms, or briefly explain why the item is not applicable/relevant for your study

Like this"In terms of objective clinical indicators, our clinical trial reported significant differences in the maximum mouth opening and frequency of mouth-opening exercises, a finding that is promising and demonstrates better clinical outcomes compared to previous remote support studies[44, 47, 48]. After the intervention, the maximum mouth opening value increased from 36.98 to 39.05, which corresponds to improvements in physical indicators related to oral health. In the intervention group, the average increase in maximum mouth opening was 2.07 mm, with a median increase ranging from 1.5 mm to 2.6 mm. Additionally, within four weeks after discharge, the number of subjects with clenched jaws decreased, and the magnitude of this change was comparable to findings in previous studies involving head and neck cancer patients[49, 50], indicating the potential benefits of the intervention in improving specific health behaviors.Furthermore, treatment adherence is a critical determinant of the success of any preventive exercise program[11, 51]. We observed that the intervention group performed significantly more exercises per week (10.0 ± 3.5) compared to the control group (4.2 ± 2.6), with a p-value of 0.001, demonstrating the significant impact of our developed application on improving patient adherence to home rehabilitation exercises. However, despite the significant improvement in maximum mouth opening, 10% of participants in the experimental group still exhibited clenched jaws during follow-up, suggesting that a longer intervention period may be required,and our study did not find that the application was related to the improvement of mouth opening symmetry, which is contrary to previous studies. Part of the reason may be due to the different duration of the study and the difference in the type and level of functional support provided.[52]A comprehensive strengthening of the facial nerve structure and musculature is required for symmetry to improve; short-term interventions are ineffective.[53]
In terms of improvements in rehabilitation training behaviors, the remote healthcare intervention group in this study, compared to the standard care control group, demonstrated a significant enhancement in patients' perceived benefits and self-efficacy ($p < 0.05$). These findings are consistent with previous studies, such as the research by Jeihooni et al., which examined the impact of remote healthcare-based educational programs on improving oral health training behaviors in pregnant women. The results of this randomized controlled trial showed significant improvements in oral health beliefs and clinical training indicators, similar to the present study, suggesting that remote healthcare interventions can effectively improve individuals' health cognition and behavior change. This supports the rational use of such models to improve health behaviors in a wide range of environments and populations[54, 55].The remote healthcare tool developed in this study provides patients with daily training reminders, real-time supervision of training movements, corrective prompts, and periodic visual training score reports. This semi-supervised, freely accessible intervention appears to be an effective choice for enhancing perceived benefits and self-efficacy[56]. Compared to traditional educational methods, it is more cost-effective and offers greater penetration[57]. The significant improvements in the Perceived Severity and Perceived Benefits indicators ($p < 0.05$) further validate the effectiveness of the intervention. Our application, utilizing the EffiFCNN-ResNet deep learning model and computer vision, tracks participants' mouth-opening movements to facilitate appropriate exercise and training plans while providing personalized feedback for patients at different levels. This may also effectively promote the growth of self-efficacy among patients, thereby contributing to clinical evidence for personalized healthcare design indicators in remote healthcare, as reported in a meta-analysis[58]."

22-ii) Highlight unanswered new questions, suggest future research

Highlight unanswered new questions, suggest future research.

	1	2	3	4	5	
subitem not at all important	○	○	○	○	◉	essential

清除所选内容

Does your paper address subitem 22-ii?

Copy and paste relevant sections from the manuscript (include quotes in quotation marks "like this" to indicate direct quotes from your manuscript), or elaborate on this item by providing additional information not in the ms, or briefly explain why the item is not applicable/relevant for your study

您的回答

20) Trial limitations, addressing sources of potential bias, imprecision, and, if relevant, multiplicity of analyses

20-i) Typical limitations in ehealth trials

Typical limitations in ehealth trials: Participants in ehealth trials are rarely blinded. Ehealth trials often look at a multiplicity of outcomes, increasing risk for a Type I error. Discuss biases due to non-use of the intervention/usability issues, biases through informed consent procedures, unexpected events.

	1	2	3	4	5	
subitem not at all important	○	○	○	○	◉	essential

清除所选内容

Does your paper address subitem 20-i? *

Copy and paste relevant sections from the manuscript (include quotes in quotation marks "like this" to indicate direct quotes from your manuscript), or elaborate on this item by providing additional information not in the ms, or briefly explain why the item is not applicable/relevant for your study

Like this"However, our investigation is not without its limitations. To begin with, it should be noted that the sample size is limited and comprises individuals from several nearby tumor departments. This may result in a preponderance of population distribution, which is not ideal for deriving generalizable research conclusions. Furthermore, it was observed during the pre-experiment phase that certain patients lacking proficiency in digital health were unable to independently debug the apparatus utilized in the experimental procedure, and as an intervention application based on the augmented reality environment, it is unsuitable for patients who are sensitive to the sensory stimulation on a design level. Moreover, while experiments may impose constraints on substantial user engagement, the long-term sustainability of user engagement and activity remains uncertain. The advancement of tools for capturing, monitoring, and evaluating non-rigid facial models has been accompanied by a wide range of human facial expressions and movements, such as intricate mouth movements, subtle eye movements, and eyebrow movements. The recognition accuracy of the model we developed still has room for improvement.It is particularly difficult to capture and trace these subtle variations precisely, particularly under low-resolution or real-time conditions.Resource limitations may have an impact on the response time and user experience of the system[69]. The experiment ultimately only lasted four weeks,moving forward, it will be imperative to increase the duration of the experiment and enhance the design of the application with a specific , in order to further optimize the outcomes. In conclusion, future research should investigate the applicability of Opencare to patients with NPC residing in diverse and complex living environments and at various phases of rehabilitation. Furthermore, in light of technological progress and user input integration, the ongoing refinement and expansion of application functionalities will be critical factors in guaranteeing their sustained efficacy and broad applicability."

21) Generalisability (external validity, applicability) of the trial findings

NPT: External validity of the trial findings according to the intervention, comparators, patients, and care providers or centers involved in the trial

21-i) Generalizability to other populations

Generalizability to other populations: In particular, discuss generalizability to a general Internet population, outside of a RCT setting, and general patient population, including applicability of the study results for other organizations

	1	2	3	4	5	
subitem not at all important	○	◉	○	○	○	essential

清除所选内容

Does your paper address subitem 21-i?

Copy and paste relevant sections from the manuscript (include quotes in quotation marks "like this" to indicate direct quotes from your manuscript), or elaborate on this item by providing additional information not in the ms, or briefly explain why the item is not applicable/relevant for your study

您的回答

21-ii) Discuss if there were elements in the RCT that would be different in a routine application setting

Discuss if there were elements in the RCT that would be different in a routine application setting (e.g., prompts/reminders, more human involvement, training sessions or other co-interventions) and what impact the omission of these elements could have on use, adoption, or outcomes if the intervention is applied outside of a RCT setting.

	1	2	3	4	5	
subitem not at all important	○	◉	○	○	○	essential

清除所选内容

Does your paper address subitem 21-ii?

Copy and paste relevant sections from the manuscript (include quotes in quotation marks "like this" to indicate direct quotes from your manuscript), or elaborate on this item by providing additional information not in the ms, or briefly explain why the item is not applicable/relevant for your study

您的回答

OTHER INFORMATION

23) Registration number and name of trial registry

Does your paper address CONSORT subitem 23? *

Copy and paste relevant sections from the manuscript (include quotes in quotation marks "like this" to indicate direct quotes from your manuscript), or elaborate on this item by providing additional information not in the ms, or briefly explain why the item is not applicable/relevant for your study

Clinical trial registration:ChiCTR2400090305

24) Where the full trial protocol can be accessed, if available

Does your paper address CONSORT subitem 24? *

Cite a Multimedia Appendix, other reference, or copy and paste relevant sections from the manuscript (include quotes in quotation marks "like this" to indicate direct quotes from your manuscript), or elaborate on this item by providing additional information not in the ms, or briefly explain why the item is not applicable/relevant for your study

like this "3.2.2 Involvement
The control group, consisting of participants receiving conventional care, was informed of the benefits of regular mouth-opening exercises and instructed to maintain their usual lifestyle. The duration and intensity of the exercises were carefully adjusted to avoid discomfort. Researchers provided participants with instructional video demonstrations for the mouth-opening exercises at no cost, along with an application designed to track daily maximum mouth opening, symmetry, and the frequency of weekly video-guided practice. The video was meticulously designed and recorded by medical professionals from the oncology radiation department, following strict protocols. It was subsequently validated by a multidisciplinary team of experts, including oncologists specializing in head and neck cancer, oral and maxillofacial surgeons, head and neck tumor specialists, rehabilitation physicians, and physical therapists.
In contrast, the intervention group utilized a health application developed by our team, which included the aforementioned instructional videos and a monitoring feedback feature for tracking exercise performance. Throughout the study, all participants were instructed to refrain from engaging in any additional conventional exercises. Researchers conducted weekly phone follow-ups to assess participants' recovery progress and address any inquiries. Furthermore, another research assistant provided detailed instructions on using the application and answered questions in a dedicated chat group. After each training session, the app recorded users' maximum mouth opening and symmetry, while also collecting data on fatigue levels and user experience for subsequent qualitative analysis.
3.2.3 Following testing
Following a four-week intervention period, all participants underwent an assessment to evaluate their maximal mouth opening, along with other outcome measures and relevant indicators. The data were collected using the same measurement method as the preliminary examination, ensuring consistency for a quantitative analysis of both intra-group and inter-group comparisons.
3.2.4 Documents of Safety
No adverse event report is included with the application. In the event that participants encountered significant respiratory distress, palpitations, or any other form of discomfort during the course of the experiment, they were mandated to promptly cease their training and resume resting position. Those affected were advised to seek medical attention immediately, and the symptoms were notably severe. Serious adverse events are notified to the ethics committee, while any unexpected adverse events are reported to the researchers.
"

25) Sources of funding and other support (such as supply of drugs), role of funders

Does your paper address CONSORT subitem 25? *

Copy and paste relevant sections from the manuscript (include quotes in quotation marks "like this" to indicate direct quotes from your manuscript), or elaborate on this item by providing additional information not in the ms, or briefly explain why the item is not applicable/relevant for your study

Like this"Funding Information
This study was supported by the National Natural Science Foundation of China （72471140）,National Key R&D Program (Grant No. 2022YFB3303303,First label) and the Fundamental Research Funds for the Central Universities (Grant No. YG2023ZD10).."

X27) Conflicts of Interest (not a CONSORT item)

X27-i) State the relation of the study team towards the system being evaluated

In addition to the usual declaration of interests (financial or otherwise), also state the relation of the study team towards the system being evaluated, i.e., state if the authors/evaluators are distinct from or identical with the developers/sponsors of the intervention.

	1	2	3	4	5	
subitem not at all important	○	○	○	○	◉	essential

Clear the selection

Does your paper address subitem X27-i?

Copy and paste relevant sections from the manuscript (include quotes in quotation marks "like this" to indicate direct quotes from your manuscript), or elaborate on this item by providing additional information not in the ms, or briefly explain why the item is not applicable/relevant for your study

Your answer

About the CONSORT EHEALTH checklist

As a result of using this checklist, did you make changes in your manuscript? *

- ( ) yes, major changes
- ( ) yes, minor changes
- (•) no

What were the most important changes you made as a result of using this checklist?

Your answer

How much time did you spend on going through the checklist INCLUDING making changes in your manuscript *

I spent a full workday carefully reviewing the content of each form.

As a result of using this checklist, do you think your manuscript has improved? *

- (•) yes
- ( ) no
- ( ) Other:

Would you like to become involved in the CONSORT EHEALTH group?

This would involve for example becoming involved in participating in a workshop and writing an "Explanation and Elaboration" document

- ( ) yes
- ( ) no
- ( ) Other:

Any other comments or questions on CONSORT EHEALTH

Your answer

STOP - Save this form as PDF before you click submit

To generate a record that you filled in this form, we recommend to generate a PDF of this page (on a Mac, simply select "print" and then select "print as PDF") before you submit it.

When you submit your (revised) paper to JMIR, please upload the PDF as supplementary file.

Don't worry if some text in the textboxes is cut off, as we still have the complete information in our database. Thank you!

Final step: Click submit !

Click submit so we have your answers in our database!

提交 清除表单内容

切勿通过 Google 表单提交密码。

此内容不是由 Google 所创建，Google 不对其作任何担保。 - 服务条款 - 隐私权政策

Does this form look suspicious? 报告

Google 表单
